# Supplementary material for: Caregivers’ experience of seeking care for adolescents with sickle cell disease in a tertiary care hospital in Bahrain
Source: PLoS One. 2022 Apr 7;17(4):e0266501. doi: 10.1371/journal.pone.0266501 (PMC8989311; doi:10.1371/journal.pone.0266501)
Supplement: S2 Data — (PDF) [file pone.0266501.s002.pdf]

```
GET
  FILE='C:\Users\khadi\Desktop\SPSS.sav'.
DATASET NAME DataSet1 WINDOW=FRONT.
FREQUENCIES VARIABLES=caregiver_age
  /STATISTICS=STDDEV MEAN
  /ORDER=ANALYSIS.
```

# Frequencies

**Notes**

| | | |
|---|---|---|
| Output Created | | 31-OCT-2020 18:41:26 |
| Comments | | |
| Input | Data | C:\Users\khadi\Desktop\SPSS.sav |
| | Active Dataset | DataSet1 |
| | Filter | <none> |
| | Weight | <none> |
| | Split File | <none> |
| | N of Rows in Working Data File | 101 |
| Missing Value Handling | Definition of Missing | User-defined missing values are treated as missing. |
| | Cases Used | Statistics are based on all cases with valid data. |
| Syntax | | FREQUENCIES VARIABLES=caregiver_age /STATISTICS=STDDEV MEAN /ORDER=ANALYSIS. |
| Resources | Processor Time | 00:00:00.00 |
| | Elapsed Time | 00:00:00.00 |

```
[DataSet1] C:\Users\khadi\Desktop\SPSS.sav
```

## Statistics

Caregiver age

| | | |
|---|---|---|
| N | Valid | 100 |
| | Missing | 1 |
| Mean | | 44.24 |
| Std. Deviation | | 6.254 |

## Caregiver age

| | | Frequency | Percent | Valid Percent | Cumulative Percent |
|---|---|---|---|---|---|
| Valid | 18 | 1 | 1.0 | 1.0 | 1.0 |
| | 30 | 1 | 1.0 | 1.0 | 2.0 |

**Caregiver age**

| | | Frequency | Percent | Valid Percent | Cumulative Percent |
|---|---|---|---|---|---|
| | 32 | 1 | 1.0 | 1.0 | 3.0 |
| | 34 | 2 | 2.0 | 2.0 | 5.0 |
| | 35 | 3 | 3.0 | 3.0 | 8.0 |
| | 36 | 2 | 2.0 | 2.0 | 10.0 |
| | 37 | 2 | 2.0 | 2.0 | 12.0 |
| | 38 | 4 | 4.0 | 4.0 | 16.0 |
| | 39 | 2 | 2.0 | 2.0 | 18.0 |
| | 40 | 5 | 5.0 | 5.0 | 23.0 |
| | 41 | 7 | 6.9 | 7.0 | 30.0 |
| | 42 | 9 | 8.9 | 9.0 | 39.0 |
| | 43 | 4 | 4.0 | 4.0 | 43.0 |
| | 44 | 2 | 2.0 | 2.0 | 45.0 |
| | 45 | 15 | 14.9 | 15.0 | 60.0 |
| | 46 | 6 | 5.9 | 6.0 | 66.0 |
| | 47 | 8 | 7.9 | 8.0 | 74.0 |
| | 48 | 3 | 3.0 | 3.0 | 77.0 |
| | 49 | 5 | 5.0 | 5.0 | 82.0 |
| | 50 | 3 | 3.0 | 3.0 | 85.0 |
| | 51 | 2 | 2.0 | 2.0 | 87.0 |
| | 52 | 1 | 1.0 | 1.0 | 88.0 |
| | 53 | 6 | 5.9 | 6.0 | 94.0 |
| | 54 | 1 | 1.0 | 1.0 | 95.0 |
| | 55 | 3 | 3.0 | 3.0 | 98.0 |
| | 56 | 1 | 1.0 | 1.0 | 99.0 |
| | 57 | 1 | 1.0 | 1.0 | 100.0 |
| | Total | 100 | 99.0 | 100.0 | |
| Missing | 9999 | 1 | 1.0 | | |
| Total | | 101 | 100.0 | | |

```
FREQUENCIES VARIABLES=caregivers_ages_C caregiver_gender marital_status patien
t_relationship
    occupation educational_level
  /ORDER=ANALYSIS.
```

# Frequencies

## Notes

| | | |
|---|---|---|
| Output Created | | 31-OCT-2020 18:42:57 |
| Comments | | |
| Input | Data | C:<br>\Users\khadi\Desktop\SPSS.sav |
| | Active Dataset | DataSet1 |
| | Filter | <none> |
| | Weight | <none> |
| | Split File | <none> |
| | N of Rows in Working Data File | 101 |
| Missing Value Handling | Definition of Missing | User-defined missing values are treated as missing. |
| | Cases Used | Statistics are based on all cases with valid data. |
| Syntax | | FREQUENCIES VARIABLES=caregivers_ages_C caregiver_gender marital_status patient_relationship occupation educational_level /ORDER=ANALYSIS. |
| Resources | Processor Time | 00:00:00.00 |
| | Elapsed Time | 00:00:00.00 |

## Statistics

| | | caregivers_ages_C | caregiver gender | Marital status | relationship to the patient | your current occupation |
|---|---|---|---|---|---|---|
| N | Valid | 100 | 101 | 101 | 101 | 101 |
| | Missing | 1 | 0 | 0 | 0 | 0 |

## Statistics

| | | educational level |
|---|---|---|
| N | Valid | 101 |
| | Missing | 0 |

# Frequency Table

**caregivers_ages_C**

| | | Frequency | Percent | Valid Percent | Cumulative Percent |
|---|---|---|---|---|---|
| Valid | less than 40 | 18 | 17.8 | 18.0 | 18.0 |
| | 40-50 | 67 | 66.3 | 67.0 | 85.0 |
| | more than 50 | 15 | 14.9 | 15.0 | 100.0 |
| | Total | 100 | 99.0 | 100.0 | |
| Missing | 9999.00 | 1 | 1.0 | | |
| Total | | 101 | 100.0 | | |

**caregiver gender**

| | | Frequency | Percent | Valid Percent | Cumulative Percent |
|---|---|---|---|---|---|
| Valid | male | 23 | 22.8 | 22.8 | 22.8 |
| | female | 78 | 77.2 | 77.2 | 100.0 |
| | Total | 101 | 100.0 | 100.0 | |

**Marital status**

| | | Frequency | Percent | Valid Percent | Cumulative Percent |
|---|---|---|---|---|---|
| Valid | married | 93 | 92.1 | 92.1 | 92.1 |
| | single | 2 | 2.0 | 2.0 | 94.1 |
| | divorced | 2 | 2.0 | 2.0 | 96.0 |
| | widowed | 4 | 4.0 | 4.0 | 100.0 |
| | Total | 101 | 100.0 | 100.0 | |

**relationship to the patient**

| | | Frequency | Percent | Valid Percent | Cumulative Percent |
|---|---|---|---|---|---|
| Valid | mother | 74 | 73.3 | 73.3 | 73.3 |
| | father | 23 | 22.8 | 22.8 | 96.0 |
| | sister | 2 | 2.0 | 2.0 | 98.0 |
| | brother | 1 | 1.0 | 1.0 | 99.0 |
| | other | 1 | 1.0 | 1.0 | 100.0 |
| | Total | 101 | 100.0 | 100.0 | |

### your current occupation

| | | Frequency | Percent | Valid Percent | Cumulative Percent |
|---|---|---|---|---|---|
| Valid | employed: (Free work/Public or Private employee/Retired) | 52 | 51.5 | 51.5 | 51.5 |
| | unemployed: (No job/Student/Looking for Job) | 49 | 48.5 | 48.5 | 100.0 |
| | Total | 101 | 100.0 | 100.0 | |

### educational level

| | | Frequency | Percent | Valid Percent | Cumulative Percent |
|---|---|---|---|---|---|
| Valid | illiterate | 1 | 1.0 | 1.0 | 1.0 |
| | primary | 3 | 3.0 | 3.0 | 4.0 |
| | intermediate | 15 | 14.9 | 14.9 | 18.8 |
| | High school | 42 | 41.6 | 41.6 | 60.4 |
| | diploma | 13 | 12.9 | 12.9 | 73.3 |
| | B.S.C | 20 | 19.8 | 19.8 | 93.1 |
| | master | 7 | 6.9 | 6.9 | 100.0 |
| | Total | 101 | 100.0 | 100.0 | |

```
FREQUENCIES VARIABLES=patient_age_C
  /ORDER=ANALYSIS.
```

## Frequencies

## Notes

| | | |
|---|---|---|
| Output Created | | 31-OCT-2020 18:47:39 |
| Comments | | |
| Input | Data | C: \Users\khadi\Desktop\SPSS.sav |
| | Active Dataset | DataSet1 |
| | Filter | <none> |
| | Weight | <none> |
| | Split File | <none> |
| | N of Rows in Working Data File | 101 |
| Missing Value Handling | Definition of Missing | User-defined missing values are treated as missing. |
| | Cases Used | Statistics are based on all cases with valid data. |
| Syntax | | FREQUENCIES VARIABLES=patient_age_C /ORDER=ANALYSIS. |
| Resources | Processor Time | 00:00:00.02 |
| | Elapsed Time | 00:00:00.00 |

## Statistics

patient_age_C

| | | |
|---|---|---|
| N | Valid | 101 |
| | Missing | 0 |

## patient_age_C

| | | Frequency | Percent | Valid Percent | Cumulative Percent |
|---|---|---|---|---|---|
| Valid | 10-12 Yr | 33 | 32.7 | 32.7 | 32.7 |
| | 13-15 Yr | 42 | 41.6 | 41.6 | 74.3 |
| | 16-18 Yr | 26 | 25.7 | 25.7 | 100.0 |
| | Total | 101 | 100.0 | 100.0 | |

```
FREQUENCIES VARIABLES=patient_age
 /STATISTICS=STDDEV MEAN
```

```
/ORDER=ANALYSIS.
```

# Frequencies

## Notes

| | | |
|---|---|---|
| Output Created | | 31-OCT-2020 18:48:28 |
| Comments | | |
| Input | Data | C:\Users\khadi\Desktop\SPSS.sav |
| | Active Dataset | DataSet1 |
| | Filter | <none> |
| | Weight | <none> |
| | Split File | <none> |
| | N of Rows in Working Data File | 101 |
| Missing Value Handling | Definition of Missing | User-defined missing values are treated as missing. |
| | Cases Used | Statistics are based on all cases with valid data. |
| Syntax | | FREQUENCIES VARIABLES=patient_age /STATISTICS=STDDEV MEAN /ORDER=ANALYSIS. |
| Resources | Processor Time | 00:00:00.00 |
| | Elapsed Time | 00:00:00.00 |

## Statistics

Patient age

| | | |
|---|---|---|
| N | Valid | 101 |
| | Missing | 0 |
| Mean | | 13.73 |
| Std. Deviation | | 2.370 |

**Patient age**

| | | Frequency | Percent | Valid Percent | Cumulative Percent |
|---|---|---|---|---|---|
| Valid | 10 | 10 | 9.9 | 9.9 | 9.9 |
| | 11 | 11 | 10.9 | 10.9 | 20.8 |
| | 12 | 12 | 11.9 | 11.9 | 32.7 |
| | 13 | 18 | 17.8 | 17.8 | 50.5 |
| | 14 | 11 | 10.9 | 10.9 | 61.4 |
| | 15 | 13 | 12.9 | 12.9 | 74.3 |
| | 16 | 9 | 8.9 | 8.9 | 83.2 |
| | 17 | 11 | 10.9 | 10.9 | 94.1 |
| | 18 | 6 | 5.9 | 5.9 | 100.0 |
| | Total | 101 | 100.0 | 100.0 | |

```
FREQUENCIES VARIABLES=challenges_on_way2 challenges_on_way3
  /ORDER=ANALYSIS.
```

# Frequencies

### Notes

| | | |
|---|---|---|
| Output Created | | 31-OCT-2020 18:49:31 |
| Comments | | |
| Input | Data | C:\Users\khadi\Desktop\SPSS.sav |
| | Active Dataset | DataSet1 |
| | Filter | <none> |
| | Weight | <none> |
| | Split File | <none> |
| | N of Rows in Working Data File | 101 |
| Missing Value Handling | Definition of Missing | User-defined missing values are treated as missing. |
| | Cases Used | Statistics are based on all cases with valid data. |
| Syntax | | FREQUENCIES VARIABLES=challenges_on_way2 challenges_on_way3 /ORDER=ANALYSIS. |
| Resources | Processor Time | 00:00:00.00 |
| | Elapsed Time | 00:00:00.00 |

### Statistics

| | | Road Traffic | Insufficient Parking |
|---|---|---|---|
| N | Valid | 101 | 101 |
| | Missing | 0 | 0 |

## Frequency Table

### Road Traffic

| | | Frequency | Percent | Valid Percent | Cumulative Percent |
|---|---|---|---|---|---|
| Valid | no | 74 | 73.3 | 73.3 | 73.3 |
| | yes | 27 | 26.7 | 26.7 | 100.0 |
| | Total | 101 | 100.0 | 100.0 | |

**Insufficient Parking**

| | | Frequency | Percent | Valid Percent | Cumulative Percent |
|---|---|---|---|---|---|
| Valid | no | 48 | 47.5 | 47.5 | 47.5 |
| | yes | 53 | 52.5 | 52.5 | 100.0 |
| | Total | 101 | 100.0 | 100.0 | |

```
FREQUENCIES VARIABLES=first_destination1 first_destination2 first_destination3
 first_destination4
  /ORDER=ANALYSIS.
```

## Frequencies

**Notes**

| | | |
|---|---|---|
| Output Created | | 31-OCT-2020 18:51:46 |
| Comments | | |
| Input | Data | C:\Users\khadi\Desktop\SPSS.sav |
| | Active Dataset | DataSet1 |
| | Filter | <none> |
| | Weight | <none> |
| | Split File | <none> |
| | N of Rows in Working Data File | 101 |
| Missing Value Handling | Definition of Missing | User-defined missing values are treated as missing. |
| | Cases Used | Statistics are based on all cases with valid data. |
| Syntax | | FREQUENCIES VARIABLES=first_destination1 first_destination2 first_destination3 first_destination4 /ORDER=ANALYSIS. |
| Resources | Processor Time | 00:00:00.02 |
| | Elapsed Time | 00:00:00.00 |

## Statistics

| | | Health Centre | SMC | Private Clinic | Traditional Medicine |
|---|---|---|---|---|---|
| N | Valid | 101 | 101 | 101 | 101 |
| | Missing | 0 | 0 | 0 | 0 |

# Frequency Table

## Health Centre

| | | Frequency | Percent | Valid Percent | Cumulative Percent |
|---|---|---|---|---|---|
| Valid | no | 44 | 43.6 | 43.6 | 43.6 |
| | yes | 57 | 56.4 | 56.4 | 100.0 |
| | Total | 101 | 100.0 | 100.0 | |

## SMC

| | | Frequency | Percent | Valid Percent | Cumulative Percent |
|---|---|---|---|---|---|
| Valid | no | 75 | 74.3 | 74.3 | 74.3 |
| | yes | 26 | 25.7 | 25.7 | 100.0 |
| | Total | 101 | 100.0 | 100.0 | |

## Private Clinic

| | | Frequency | Percent | Valid Percent | Cumulative Percent |
|---|---|---|---|---|---|
| Valid | no | 94 | 93.1 | 93.1 | 93.1 |
| | yes | 7 | 6.9 | 6.9 | 100.0 |
| | Total | 101 | 100.0 | 100.0 | |

## Traditional Medicine

| | | Frequency | Percent | Valid Percent | Cumulative Percent |
|---|---|---|---|---|---|
| Valid | no | 85 | 84.2 | 84.2 | 84.2 |
| | yes | 16 | 15.8 | 15.8 | 100.0 |
| | Total | 101 | 100.0 | 100.0 | |

```
CROSSTABS
```

```
  /TABLES=first_destination1 BY why_destination_Distance why_destination_Quali
ty
  /FORMAT=AVALUE TABLES
  /CELLS=COUNT
  /COUNT ROUND CELL.
```

## Crosstabs

**Notes**

| | | |
|---|---|---|
| Output Created | | 31-OCT-2020 18:55:29 |
| Comments | | |
| Input | Data | C:\Users\khadi\Desktop\SPSS.sav |
| | Active Dataset | DataSet1 |
| | Filter | <none> |
| | Weight | <none> |
| | Split File | <none> |
| | N of Rows in Working Data File | 101 |
| Missing Value Handling | Definition of Missing | User-defined missing values are treated as missing. |
| | Cases Used | Statistics for each table are based on all the cases with valid data in the specified range(s) for all variables in each table. |
| Syntax | | CROSSTABS<br><br>/TABLES=first_destination1 BY why_destination_Distance why_destination_Quality<br>/FORMAT=AVALUE TABLES<br>/CELLS=COUNT<br>/COUNT ROUND CELL. |
| Resources | Processor Time | 00:00:00.00 |
| | Elapsed Time | 00:00:00.01 |
| | Dimensions Requested | 2 |
| | Cells Available | 524245 |

### Case Processing Summary

| | Cases | | | | | |
|---|---|---|---|---|---|---|
| | Valid | | Missing | | Total | |
| | N | Percent | N | Percent | N | Percent |
| Health Centre * Distance | 101 | 100.0% | 0 | 0.0% | 101 | 100.0% |
| Health Centre * Quality of Service | 101 | 100.0% | 0 | 0.0% | 101 | 100.0% |

### Health Centre * Distance Crosstabulation

Count

| | | Distance | | |
|---|---|---|---|---|
| | | no | yes | Total |
| Health Centre | no | 43 | 1 | 44 |
| | yes | 16 | 41 | 57 |
| Total | | 59 | 42 | 101 |

### Health Centre * Quality of Service Crosstabulation

Count

| | | Quality of Service | | |
|---|---|---|---|---|
| | | no | yes | Total |
| Health Centre | no | 23 | 21 | 44 |
| | yes | 43 | 14 | 57 |
| Total | | 66 | 35 | 101 |

```
CROSSTABS
 /TABLES=first_destination2 BY why_destination_Quality why_destination_Faster
 /FORMAT=AVALUE TABLES
 /CELLS=COUNT
 /COUNT ROUND CELL.
```

## Crosstabs

## Notes

| | | |
|---|---|---|
| Output Created | | 31-OCT-2020 18:56:11 |
| Comments | | |
| Input | Data | C:<br>\Users\khadi\Desktop\SPSS.sav |
| | Active Dataset | DataSet1 |
| | Filter | <none> |
| | Weight | <none> |
| | Split File | <none> |
| | N of Rows in Working Data File | 101 |
| Missing Value Handling | Definition of Missing | User-defined missing values are treated as missing. |
| | Cases Used | Statistics for each table are based on all the cases with valid data in the specified range(s) for all variables in each table. |
| Syntax | | CROSSTABS<br><br>/TABLES=first_destination 2 BY why_destination_Quality why_destination_Faster<br>/FORMAT=AVALUE TABLES<br>/CELLS=COUNT<br>/COUNT ROUND CELL. |
| Resources | Processor Time | 00:00:00.00 |
| | Elapsed Time | 00:00:00.01 |
| | Dimensions Requested | 2 |
| | Cells Available | 524245 |

## Case Processing Summary

| | Cases | | | | | |
|---|---|---|---|---|---|---|
| | Valid | | Missing | | Total | |
| | N | Percent | N | Percent | N | Percent |
| SMC * Quality of Service | 101 | 100.0% | 0 | 0.0% | 101 | 100.0% |
| SMC * Faster Response | 101 | 100.0% | 0 | 0.0% | 101 | 100.0% |

**SMC * Quality of Service Crosstabulation**

Count

| | | Quality of Service | | |
|---|---|---|---|---|
| | | no | yes | Total |
| SMC | no | 57 | 18 | 75 |
| | yes | 9 | 17 | 26 |
| Total | | 66 | 35 | 101 |

**SMC * Faster Response Crosstabulation**

Count

| | | Faster Response | | |
|---|---|---|---|---|
| | | no | yes | Total |
| SMC | no | 67 | 8 | 75 |
| | yes | 26 | 0 | 26 |
| Total | | 93 | 8 | 101 |

```
 * Custom Tables.
CTABLES
  /VLABELS VARIABLES=why_destination_Distance why_destination_Quality
    why_destination_doctors_availability why_destination_Faster why_destinatio
n_medication
    why_destination_avoid_admission first_destination1 first_destination2 firs
t_destination3
    first_destination4
    DISPLAY=LABEL
  /TABLE why_destination_Distance + why_destination_Quality + why_destination_
doctors_availability
    + why_destination_Faster + why_destination_medication + why_destination_av
oid_admission BY
    first_destination1 [COUNT F40.0] + first_destination2 [COUNT F40.0] + firs
t_destination3 [COUNT
    F40.0] + first_destination4 [COUNT F40.0]
  /CATEGORIES VARIABLES=why_destination_Distance why_destination_Quality
    why_destination_doctors_availability why_destination_Faster why_destinatio
n_medication
    why_destination_avoid_admission [1.00] EMPTY=INCLUDE
```

```
/CATEGORIES VARIABLES=first_destination1[1.00] EMPTY=INCLUDE
/CATEGORIES VARIABLES=first_destination2[1.00] EMPTY=INCLUDE
/CATEGORIES VARIABLES=first_destination3[1.00] EMPTY=INCLUDE
/CATEGORIES VARIABLES=first_destination4[1.00] EMPTY=INCLUDE
/CRITERIA CILEVEL=95.
```

# Custom Tables

**Notes**

| | | |
|---|---|---|
| Output Created | | 31-OCT-2020 18:58:20 |
| Comments | | |
| Input | Data | C:\Users\khadi\Desktop\SPSS.sav |
| | Active Dataset | DataSet1 |
| | Filter | <none> |
| | Weight | <none> |
| | Split File | <none> |
| | N of Rows in Working Data File | 101 |

## Notes

| | |
|---|---|
| Syntax | CTABLES<br>/VLABELS VARIABLES=why_destination_Distance why_destination_Quality<br><br>why_destination_doctors_availability why_destination_Faster why_destination_medication<br><br>why_destination_avoid_admission first_destination1 first_destination2 first_destination3<br>first_destination4<br>DISPLAY=LABEL<br>/TABLE why_destination_Distance + why_destination_Quality +<br>why_destination_doctors_availability<br>+<br>why_destination_Faster + why_destination_medication + why_destination_avoid_admission BY<br>first_destination1 [COUNT F40.0] + first_destination2 [COUNT F40.0] + first_destination3 [COUNT<br>F40.0] + first_destination4 [COUNT F40.0]<br>/CATEGORIES VARIABLES=why_destination_Distance why_destination_Quality<br><br>why_destination_doctors_availability why_destination_Faster why_destination_medication<br><br>why_destination_avoid_admission [1.00] EMPTY=INCLUDE<br>/CATEGORIES VARIABLES=first_destination1 [1.00] EMPTY=INCLUDE<br>/CATEGORIES VARIABLES=first_destination2 [1.00] EMPTY=INCLUDE<br>/CATEGORIES [illegible] |

## Notes

| | | |
|---|---|---|
| Resources | Processor Time | 00:00:00.00 |
| | Elapsed Time | 00:00:00.01 |

## Warnings

Some subtables in table 1 are empty. They may not display properly if EMPTY = EXCLUDE.

| | | Health Centre | SMC | Private Clinic | Traditional Medicine |
|---|---|---|---|---|---|
| | | yes | yes | yes | yes |
| | | Count | Count | Count | Count |
| Distance | yes | 41 | 1 | 0 | 1 |
| Quality of Service | yes | 14 | 17 | 2 | 4 |
| Availability of Doctors | yes | 5 | 8 | 3 | 1 |
| Faster Response | yes | 6 | 0 | 2 | 2 |
| Medication | yes | 0 | 1 | 1 | 7 |
| Avoid admission | yes | 1 | 0 | 0 | 0 |

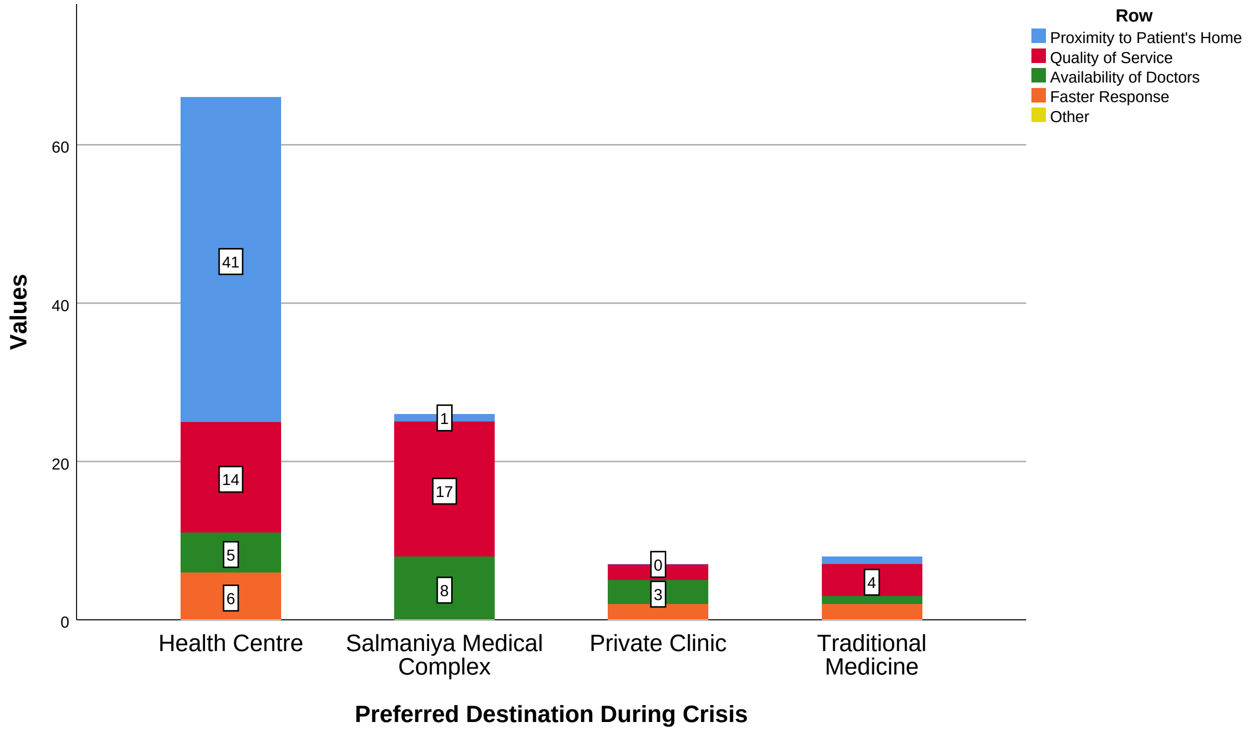

```
FREQUENCIES VARIABLES=family_support type_of_support1 type_of_support2 type_of_support3
    type_of_support_visits type_of_support_transport relation_affected_C
  /ORDER=ANALYSIS.
```

# Frequencies

**Notes**

| | | |
|---|---|---|
| Output Created | | 31-OCT-2020 19:17:40 |
| Comments | | |
| Input | Data | C:\Users\khadi\Desktop\SPSS.sav |
| | Active Dataset | DataSet1 |
| | Filter | <none> |
| | Weight | <none> |
| | Split File | <none> |
| | N of Rows in Working Data File | 101 |
| Missing Value Handling | Definition of Missing | User-defined missing values are treated as missing. |
| | Cases Used | Statistics are based on all cases with valid data. |
| Syntax | | FREQUENCIES VARIABLES=family_support type_of_support1 type_of_support2 type_of_support3 type_of_support_visits type_of_support_transport relation_affected_C /ORDER=ANALYSIS. |
| Resources | Processor Time | 00:00:00.00 |
| | Elapsed Time | 00:00:00.00 |

**Statistics**

| | | family support | Emotional Support | Financial Support | Informational Support | Visits |
|---|---|---|---|---|---|---|
| N | Valid | 101 | 101 | 101 | 101 | 101 |
| | Missing | 0 | 0 | 0 | 0 | 0 |

## Statistics

| | | Transport | relation_affected_C |
|---|---|---|---|
| N | Valid | 101 | 101 |
| | Missing | 0 | 0 |

# Frequency Table

## family support

| | | Frequency | Percent | Valid Percent | Cumulative Percent |
|---|---|---|---|---|---|
| Valid | 1 | 13 | 12.9 | 12.9 | 12.9 |
| | 2 | 4 | 4.0 | 4.0 | 16.8 |
| | 3 | 10 | 9.9 | 9.9 | 26.7 |
| | 4 | 19 | 18.8 | 18.8 | 45.5 |
| | 5 | 55 | 54.5 | 54.5 | 100.0 |
| | Total | 101 | 100.0 | 100.0 | |

## Emotional Support

| | | Frequency | Percent | Valid Percent | Cumulative Percent |
|---|---|---|---|---|---|
| Valid | no | 19 | 18.8 | 18.8 | 18.8 |
| | yes | 82 | 81.2 | 81.2 | 100.0 |
| | Total | 101 | 100.0 | 100.0 | |

## Financial Support

| | | Frequency | Percent | Valid Percent | Cumulative Percent |
|---|---|---|---|---|---|
| Valid | no | 82 | 81.2 | 81.2 | 81.2 |
| | yes | 19 | 18.8 | 18.8 | 100.0 |
| | Total | 101 | 100.0 | 100.0 | |

## Informational Support

| | | Frequency | Percent | Valid Percent | Cumulative Percent |
|---|---|---|---|---|---|
| Valid | no | 77 | 76.2 | 76.2 | 76.2 |
| | yes | 24 | 23.8 | 23.8 | 100.0 |
| | Total | 101 | 100.0 | 100.0 | |

**Visits**

| | | Frequency | Percent | Valid Percent | Cumulative Percent |
|---|---|---|---|---|---|
| Valid | no | 99 | 98.0 | 98.0 | 98.0 |
| | yes | 2 | 2.0 | 2.0 | 100.0 |
| | Total | 101 | 100.0 | 100.0 | |

**Transport**

| | | Frequency | Percent | Valid Percent | Cumulative Percent |
|---|---|---|---|---|---|
| Valid | no | 99 | 98.0 | 98.0 | 98.0 |
| | yes | 2 | 2.0 | 2.0 | 100.0 |
| | Total | 101 | 100.0 | 100.0 | |

**relation_affected_C**

| | | Frequency | Percent | Valid Percent | Cumulative Percent |
|---|---|---|---|---|---|
| Valid | relations with categories<=2 | 74 | 73.3 | 73.3 | 73.3 |
| | relations with categories>2 | 27 | 26.7 | 26.7 | 100.0 |
| | Total | 101 | 100.0 | 100.0 | |

```
FREQUENCIES VARIABLES=self_time_affected
  /ORDER=ANALYSIS.
```

# Frequencies

## Notes

| | | |
|---|---|---|
| Output Created | | 31-OCT-2020 19:18:47 |
| Comments | | |
| Input | Data | C: \Users\khadi\Desktop\SPSS.sav |
| | Active Dataset | DataSet1 |
| | Filter | <none> |
| | Weight | <none> |
| | Split File | <none> |
| | N of Rows in Working Data File | 101 |
| Missing Value Handling | Definition of Missing | User-defined missing values are treated as missing. |
| | Cases Used | Statistics are based on all cases with valid data. |
| Syntax | | FREQUENCIES VARIABLES=self_time_affected /ORDER=ANALYSIS. |
| Resources | Processor Time | 00:00:00.00 |
| | Elapsed Time | 00:00:00.00 |

## Statistics

self time affected?

| | | |
|---|---|---|
| N | Valid | 101 |
| | Missing | 0 |

## self time affected?

| | | Frequency | Percent | Valid Percent | Cumulative Percent |
|---|---|---|---|---|---|
| Valid | doesn't affect | 12 | 11.9 | 11.9 | 11.9 |
| | somtimes affect | 49 | 48.5 | 48.5 | 60.4 |
| | barely have time | 40 | 39.6 | 39.6 | 100.0 |
| | Total | 101 | 100.0 | 100.0 | |

```
USE ALL.
COMPUTE filter_$=(monthly_spend < 9999 + monthly_income < 9999).
```

```
VARIABLE LABELS filter_$ 'monthly_spend < 9999 + monthly_income < 9999 (FILTER
)'.
VALUE LABELS filter_$ 0 'Not Selected' 1 'Selected'.
FORMATS filter_$ (f1.0).
FILTER BY filter_$.
EXECUTE.
COMPUTE CHE_y=(monthly_spend / monthly_income) * 100.
EXECUTE.
RECODE CHE_equation (Lowest thru 9.99=1) (10 thru Highest=2) INTO CHE_result.
VARIABLE LABELS  CHE_result 'CHE_result'.
EXECUTE.
FILTER OFF.
USE ALL.
EXECUTE.
FREQUENCIES VARIABLES=CHE_result
  /ORDER=ANALYSIS.
```

## Frequencies

**Notes**

| | | |
|---|---|---|
| Output Created | | 31-OCT-2020 19:24:59 |
| Comments | | |
| Input | Data | C:\Users\khadi\Desktop\SPSS.sav |
| | Active Dataset | DataSet1 |
| | Filter | <none> |
| | Weight | <none> |
| | Split File | <none> |
| | N of Rows in Working Data File | 101 |
| Missing Value Handling | Definition of Missing | User-defined missing values are treated as missing. |
| | Cases Used | Statistics are based on all cases with valid data. |
| Syntax | | FREQUENCIES VARIABLES=CHE_result /ORDER=ANALYSIS. |
| Resources | Processor Time | 00:00:00.00 |
| | Elapsed Time | 00:00:00.00 |

### Statistics

CHE_result

| | | |
|---|---|---|
| N | Valid | 88 |
| | Missing | 13 |

### CHE_result

| | | Frequency | Percent | Valid Percent | Cumulative Percent |
|---|---|---|---|---|---|
| Valid | Less than 10 Percent of income (CHE_no) | 75 | 74.3 | 85.2 | 85.2 |
| | More than 10 Percent of income (CHE_yes) | 13 | 12.9 | 14.8 | 100.0 |
| | Total | 88 | 87.1 | 100.0 | |
| Missing | System | 13 | 12.9 | | |
| Total | | 101 | 100.0 | | |

### Notes

| | | |
|---|---|---|
| Output Created | | 31-OCT-2020 19:26:03 |
| Comments | | |
| Input | Data | C:\Users\khadi\Desktop\SPSS.sav |
| | Active Dataset | DataSet1 |
| | Filter | <none> |
| | Weight | <none> |
| | Split File | <none> |
| | N of Rows in Working Data File | 101 |
| Missing Value Handling | Definition of Missing | User-defined missing values are treated as missing. |
| | Cases Used | Statistics are based on all cases with valid data. |
| Syntax | | FREQUENCIES VARIABLES=monthly_spend /STATISTICS=RANGE MEAN /ORDER=ANALYSIS. |
| Resources | Processor Time | 00:00:00.00 |
| | Elapsed Time | 00:00:00.00 |

**Notes**

| | | |
|---|---|---|
| Output Created | | 31-OCT-2020 19:26:46 |
| Comments | | |
| Input | Data | C:<br>\Users\khadi\Desktop\SPSS.sav |
| | Active Dataset | DataSet1 |
| | Filter | monthly_spend < 9999 + monthly_income < 9999 (FILTER) |
| | Weight | <none> |
| | Split File | <none> |
| | N of Rows in Working Data File | 88 |
| Missing Value Handling | Definition of Missing | User-defined missing values are treated as missing. |
| | Cases Used | Statistics are based on all cases with valid data. |
| Syntax | | FREQUENCIES VARIABLES=monthly_spend<br>/STATISTICS=RANGE MEAN<br>/ORDER=ANALYSIS. |
| Resources | Processor Time | 00:00:00.00 |
| | Elapsed Time | 00:00:00.00 |

**Notes**

| | | |
|---|---|---|
| Output Created | | 31-OCT-2020 19:27:10 |
| Comments | | |
| Input | Data | C: \Users\khadi\Desktop\SPSS.sav |
| | Active Dataset | DataSet1 |
| | Filter | monthly_spend < 9999 + monthly_income < 9999 (FILTER) |
| | Weight | <none> |
| | Split File | <none> |
| | N of Rows in Working Data File | 88 |
| Missing Value Handling | Definition of Missing | User-defined missing values are treated as missing. |
| | Cases Used | Statistics are based on all cases with valid data. |
| Syntax | | FREQUENCIES VARIABLES=monthly_spend /STATISTICS=RANGE MINIMUM MAXIMUM MEAN... |
| Resources | Processor Time | 00:00:00.00 |
| | Elapsed Time | 00:00:00.00 |

```
 USE ALL.
COMPUTE filter_$=(monthly_spend < 9999 + monthly_income < 9999).
VARIABLE LABELS filter_$ 'monthly_spend < 9999 + monthly_income < 9999 (FILTER
)'.
VALUE LABELS filter_$ 0 'Not Selected' 1 'Selected'.
FORMATS filter_$ (f1.0).
FILTER BY filter_$.
EXECUTE.
FREQUENCIES VARIABLES=monthly_spend
  /STATISTICS=RANGE MINIMUM MAXIMUM MEAN
  /ORDER=ANALYSIS.
```

## Frequencies

## Notes

| | | |
|---|---|---|
| Output Created | | 31-OCT-2020 19:29:29 |
| Comments | | |
| Input | Data | C:<br>\Users\khadi\Desktop\SPSS.sav |
| | Active Dataset | DataSet1 |
| | Filter | monthly_spend < 9999 + monthly_income < 9999 (FILTER) |
| | Weight | <none> |
| | Split File | <none> |
| | N of Rows in Working Data File | 88 |
| Missing Value Handling | Definition of Missing | User-defined missing values are treated as missing. |
| | Cases Used | Statistics are based on all cases with valid data. |
| Syntax | | FREQUENCIES VARIABLES=monthly_spend<br>/STATISTICS=RANGE MINIMUM MAXIMUM MEAN... |
| Resources | Processor Time | 00:00:00.00 |
| | Elapsed Time | 00:00:00.00 |

## Statistics

monthly spent on treatment

| | | |
|---|---|---|
| N | Valid | 88 |
| | Missing | 0 |
| Mean | | 16.72 |
| Range | | 150 |
| Minimum | | 0 |
| Maximum | | 150 |

**monthly spent on treatment**

| | | Frequency | Percent | Valid Percent | Cumulative Percent |
|---|---|---|---|---|---|
| Valid | 0 | 47 | 53.4 | 53.4 | 53.4 |
| | 2 | 2 | 2.3 | 2.3 | 55.7 |
| | 3 | 1 | 1.1 | 1.1 | 56.8 |
| | 5 | 7 | 8.0 | 8.0 | 64.8 |
| | 7 | 1 | 1.1 | 1.1 | 65.9 |
| | 10 | 3 | 3.4 | 3.4 | 69.3 |
| | 12 | 1 | 1.1 | 1.1 | 70.5 |
| | 15 | 2 | 2.3 | 2.3 | 72.7 |
| | 20 | 4 | 4.5 | 4.5 | 77.3 |
| | 25 | 1 | 1.1 | 1.1 | 78.4 |
| | 30 | 5 | 5.7 | 5.7 | 84.1 |
| | 35 | 1 | 1.1 | 1.1 | 85.2 |
| | 40 | 2 | 2.3 | 2.3 | 87.5 |
| | 55 | 2 | 2.3 | 2.3 | 89.8 |
| | 70 | 1 | 1.1 | 1.1 | 90.9 |
| | 80 | 2 | 2.3 | 2.3 | 93.2 |
| | 90 | 1 | 1.1 | 1.1 | 94.3 |
| | 100 | 4 | 4.5 | 4.5 | 98.9 |
| | 150 | 1 | 1.1 | 1.1 | 100.0 |
| | Total | 88 | 100.0 | 100.0 | |

```
FREQUENCIES VARIABLES=monthly_income
  /STATISTICS=MEAN MEDIAN
  /ORDER=ANALYSIS.
```

# Frequencies

## Notes

| | | |
|---|---|---|
| Output Created | | 31-OCT-2020 19:29:55 |
| Comments | | |
| Input | Data | C: \Users\khadi\Desktop\SPSS.sav |
| | Active Dataset | DataSet1 |
| | Filter | monthly_spend < 9999 + monthly_income < 9999 (FILTER) |
| | Weight | <none> |
| | Split File | <none> |
| | N of Rows in Working Data File | 88 |
| Missing Value Handling | Definition of Missing | User-defined missing values are treated as missing. |
| | Cases Used | Statistics are based on all cases with valid data. |
| Syntax | | FREQUENCIES VARIABLES=monthly_income /STATISTICS=MEAN MEDIAN /ORDER=ANALYSIS. |
| Resources | Processor Time | 00:00:00.00 |
| | Elapsed Time | 00:00:00.00 |

## Statistics

monthly income?

| | | |
|---|---|---|
| N | Valid | 88 |
| | Missing | 0 |
| Mean | | 776.08 |
| Median | | 500.00 |

**monthly income?**

| | | Frequency | Percent | Valid Percent | Cumulative Percent |
|---|---|---|---|---|---|
| Valid | 15 | 1 | 1.1 | 1.1 | 1.1 |
| | 60 | 1 | 1.1 | 1.1 | 2.3 |
| | 200 | 4 | 4.5 | 4.5 | 6.8 |
| | 250 | 1 | 1.1 | 1.1 | 8.0 |
| | 260 | 1 | 1.1 | 1.1 | 9.1 |
| | 270 | 1 | 1.1 | 1.1 | 10.2 |
| | 300 | 13 | 14.8 | 14.8 | 25.0 |
| | 320 | 1 | 1.1 | 1.1 | 26.1 |
| | 350 | 2 | 2.3 | 2.3 | 28.4 |
| | 360 | 2 | 2.3 | 2.3 | 30.7 |
| | 370 | 3 | 3.4 | 3.4 | 34.1 |
| | 400 | 6 | 6.8 | 6.8 | 40.9 |
| | 450 | 2 | 2.3 | 2.3 | 43.2 |
| | 460 | 1 | 1.1 | 1.1 | 44.3 |
| | 470 | 1 | 1.1 | 1.1 | 45.5 |
| | 500 | 7 | 8.0 | 8.0 | 53.4 |
| | 550 | 1 | 1.1 | 1.1 | 54.5 |
| | 600 | 6 | 6.8 | 6.8 | 61.4 |
| | 610 | 1 | 1.1 | 1.1 | 62.5 |
| | 650 | 1 | 1.1 | 1.1 | 63.6 |
| | 700 | 7 | 8.0 | 8.0 | 71.6 |
| | 750 | 1 | 1.1 | 1.1 | 72.7 |
| | 800 | 3 | 3.4 | 3.4 | 76.1 |
| | 900 | 2 | 2.3 | 2.3 | 78.4 |
| | 1000 | 5 | 5.7 | 5.7 | 84.1 |
| | 1200 | 2 | 2.3 | 2.3 | 86.4 |
| | 1500 | 3 | 3.4 | 3.4 | 89.8 |
| | 1700 | 1 | 1.1 | 1.1 | 90.9 |
| | 1800 | 1 | 1.1 | 1.1 | 92.0 |
| | 2000 | 2 | 2.3 | 2.3 | 94.3 |
| | 2500 | 1 | 1.1 | 1.1 | 95.5 |
| | 3000 | 2 | 2.3 | 2.3 | 97.7 |
| | 4000 | 1 | 1.1 | 1.1 | 98.9 |
| | 5000 | 1 | 1.1 | 1.1 | 100.0 |
| | Total | 88 | 100.0 | 100.0 | |
