# Supplementary material for: Caregivers’ experience of seeking care for adolescents with sickle cell disease in a tertiary care hospital in Bahrain
Source: PLoS One. 2022 Apr 7;17(4):e0266501. doi: 10.1371/journal.pone.0266501 (PMC8989311; doi:10.1371/journal.pone.0266501)
Supplement: S3 Data — (DOCX) [file pone.0266501.s003.docx]

# **Part 1: complete list of study codes**

**Each code has the number of the IDI of the participants.**

**Objectives:**

### 1-To understand the healthcare access problems faced by caregivers while seeking care for their SCD patients.

**Code: No ursodcoxycholic acid in hospital (only in KSA)**

**Number: 1**

**Code: no entertainment facilities in child wards**

**Number:1, 2**

**Code: discharge patient despite new symptoms noted by mother**

**Number:1**

**Code: Health Center is better than hospital**

**Number: 18, 12, 17**

**Code: caregiver cleans wards bathrooms**

**Number:1**

**Code: child cannot tolerate spicy hospital food**

**Number:1**

**Code: delay reaching ER to find someone taking care of siblings**

**Number:1, 7**

**Code: husband leave work to drive to hospital**

**Number: 1,4 ,7**

**Code: Health center respond faster than hospital**

**Number:12**

**Code: Ambulance refusal to deliver to the preferred hospital**

**Number:12**

**Code: Caregiver cannot drive during crisis due to panic**

**Number:12, 4 ,7**

**Code: Dirty bathrooms in wards**

**Number: 2, 12,8, 1,10 ,19, 4 ,7**

**Code: Caregiver has private car**

**Number: 15, 10, 11,5, 9, 16**

**Code: car sometimes not available**

**Number:1 ,4**

**Code: Hospital staff are respectful and have good behavior**

**Number:15,1, 3, 14, 16 ,7**

**Code: ER beds are uncomfortable**

**Number:12, 8**

**Code: Visiting hours not restricted**

**Number: 1**

**Code: ER doctors are less experienced with sickle cell patients**

**Number: 9**

**Code: Patient admitted fast to ER**

**Number: 9, 16**

**Code: no ward for SCD/ need wards or building for SCD patients**

**Number: 9, 16 ,4, 19**

**Code: Doctors show up late in ER**

**Number: 9,4**

**Code: E department is cleaner, less crowded, and quiet in ER**

**Number: 9**

**Code: Caregivers sleep on the floor in ward**

**Number: 16, 12, 7, 17**

**Code: Atmosphere of ER is not comfortable**

**Number: 11,1,4**

**Code: Waiting in ER (hours -days) until referring to ward**

**Number: 11, 18, 8, 13,14,1, 12, 15, 9,19**

**Code: Patients waiting at ER while wards are empty**

**Number: 11**

**Code: Waiting in ER till medication intervention**

**Number: 11, 2, 18, 10, 17, 18,**

**Code: No chairs for accompanying person in ER**

**Number: 11**

**Code: Chair uncomfortable for caregiver in ER**

**Number: 16**

**Code: Nurses in ward ignorant**

**Number: 11**

**Code: Nurses in ER ignorant**

**Number: 8**

**Code: pediatrics doctors not available at adult ward**

**Number: 11, 19**

**Code: no bed for caregiver in wards (causes back pain)**

**Number: 16, 15**

**Code: Child does not like hospital food**

**Number: 11, 8, 15,1,4 ,19, 7**

**Code: No Voltaren in hospital**

**Number: 11, 10, 1**

**Code: shortage in doctors and nurses in ER**

**Number: 11, 12, 9(nurses), 19 (nurses)**

**Code: Child refuses admission to adult ward**

**Number: 11, 18, 4**

**Code: First choice for caregiver is private sector for simple cases**

**Number: 16**

**Code: beds at ward sometimes broken**

**Number:1**

**Code: no separate wad for SCD children**

**Number:1**

**Code: crowded ward rooms**

**Number:1 ,7**

**Code: bathrooms are for everyone**

**Number: 1 ,10 ,19**

**Code: Doctor cannot come to ER**

**Number:8**

**Code: Nurses less experienced in finding veins in child**

**Number:9, 8**

**Code: Crowded ER rooms**

**Number: 8**

**Code: Fewer bathrooms compared to many patients**

**Number: 17, 12, 13, 19, 2**

**Code: Bathrooms need 24hrs cleaning**

**Number:8, 17**

**Code: Uncomfortable ward beds**

**Number: 8, 1**

**Code: SCD patients need remote control beds**

**Number: 8**

**Code: tiny place for SCD patients in ER**

**Number: 8**

**Code: SCD females need separate building**

**Number: 8,18**

**Code: Long waiting hours for private room**

**Number: 16**

**Code: Patient cannot sleep quietly in public room**

**Number: 16**

**Code: Old medical facilities in ward**

**Number: 16**

**Code: Laboratory test results take longer in SMC**

**Number:16 ,19**

**Code: Laboratory test results are faster in Private hospitals**

**Number:16**

**Code: Process to go to wards is easy & fast**

**Number: 16**

**Code: Private clinic is expensive and not different in quality than public hospitals**

**Number: 9**

**Code: Caregiver chose private clinic first because of lack of trust in SMC**

**Number: 9**

**Code: SMC were less conservative when it comes to treatment than in private clinic**

**Number:9**

**Code: Caregiver prefer private room because more comfortable**

**Number: 17, 18, 16 ,7**

**Code: child dislike having shower in ward bathrooms**

**Number: 10**

**Code: Fatigue due to no beds to accompanying person(ward)**

**Number: 11, 2, 5, 13, 10, 12,1, 14, 16, 4,19**

**Code: Drugs not always available**

**Number: 13,1 ,7**

**Code: More chairs should be added to wards**

**Number: 13, 7**

**Code: no special care for SCD cases in ER**

**Number: 10**

**Code: wards need maintenance**

**Number: 10**

**Code: bathrooms only cleaned in morning**

**Number: 10 ,19**

**Code: insufficient parking lots in SMC**

**Numb: 18**

**Code: Easier admission to ward before**

**Number: 15,**

### 2- To assess the influence of being a caregiver on their own social, emotional, and financial lives.

**Code: caregivers cannot give more**

**Number:4 ,19 ,7**

**Code: caregiver health is affected by the disease**

**Number:4, 19 ,7**

**Code: caregiver’s social life is affected**

**Number: 4 ,1 ,7**

**Code: caregiver work is affected because of the disease**

**Number: 4,19, 5**

**Code: caregiver is mentally distracted**

**Number: 1**

**Code: Adolescent do not want the hospital’s Ibuprofen, so caregiver buy it**

**Number: 18**

**Code: Loaning to cover private room expenses**

**Number: 18**

**Code: Dispersion of caregiver life due to SCD**

**Number:5, 8**

**Code: Buying medicines affects budget**

**Number:12,1**

**Code: Husband is not helping financially**

**Number:12**

**Code: Husband not helping in caring of child**

**Number: 11, 8,1, 4**

**Code: Husband helping in caring of child**

**Number: 10, 17, 16**

**Code: Caregiver shocked after the child diagnosed with SCD**

**Number: 11, 14,10,9, 19**

**Code: Child relationship with caregiver is stronger compared to his sibling due to the disease and more time**

**Number: 15, 9, 19 ,1**

**Code: Siblings jealous of sick child**

**Number: 11, 15, 10, 4, 7**

**Code: Siblings not jealous of sick child**

**Number:12**

**Code: Encouraging siblings to be independent to help them if mother absent to take care of patient**

**Number: 8**

**Code: siblings adapt to sister situation**

**Number:8**

**Code: no financial problems**

**Number: 15, 13, 9, 16 ,4**

**Code: Disease has no effect on work life for caregivers**

**Number: 15**

**Code: Mother afraid of having a child with SCD**

**Number: 18 ,19, 4 ,7**

**Code: Petroleum affected budget during child admissions**

**Number: 8**

**Code: Refusal of Bahrain society of SCD to give financial support**

**Number: 8**

**Code: Pray and read Quraan a lot during crisis**

**Number: 17,4**

**Code: Work is tolerant with caregiver**

**Number:12, 1, 16**

**Code: Caregiver shocked after the child diagnosed with SCD**

**Number: 11, 14,10,9, 19**

**Code: No regrets marrying carrier husband**

**Number: 11, 10, 18**

**Code: feeling regrets marrying carrier husband**

**Number: 8 ,19, 18**

**Code: Child admission caused mother to absent**

**Number: 11, 18, 5,**

**Code: psychological stress due to child stubbornness**

**Number: 11,**

**Code: Caregiver have health insurance**

**Number:14**

**Code: Moving from full time job to part time job**

**Number: 17**

**Code: Caregiver felt sorry for child.**

**Number: 9**

**Code: Caregiver faced psychological stress from being in hospital**

**Number: 18,2, 16 ,19 ,7, 4**

**Code: Caregivers feels their condition is better than others**

**Number: 12, 15, 18,17,11, 16 ,4**

**Code: loaning money to seek medical care**

**Number: 18 ,1**

**Code: Husband provide emotional support to caregiver**

**Number: 18, 7**

**Code: Fatigue due to lack of sleep**

**Number: 18, 1, 10, 5, 13, 14 ,4 ,19, 7**

**Code: Caregiver apologize to child for having SCD**

**Number: 8, 14**

**Code: After SCD diagnosis, father is more affected**

**Number:8, 14**

**Code: Caregiver has health insurance (private hospital)**

**Number: 16**

**Code: relationship with husband affected due to child disease**

**Number:8,5, 4**

**Code: recurrent child admissions affected relationship with husband**

**Number: 8**

**Code: husband married 2nd wife to have healthy children**

**Number:8**

**Code: husband do not visit child to offend mother**

**Number:8**

**Code: Ahlam Charity gave gifts**

**Number: 16**

**Code: Caregiver is acting angry during crisis toward child**

**Number: 9**

**Code: Child’s disease affects negatively on caregiver’s relationship with other family members**

**Number:9, 4**

**Code: Work was not tolerant with caregiver’s absence**

**Number:9, 7**

**Code: Caregiver do not talk openly about her issues to people.**

**Number: 9, 16, 7**

**Code: Caregiver do not show her struggle to child**

**Number: 9**

**Code: financial crises sometimes during admissions**

**Number: 10**

**Code: caregiver try to balance finance to prevent loaning**

**Number:10**

**Code: refusal of charity to give financial support**

**Number:10, 3**

**Code: Child admission caused mother to be absent**

**Number: 11, 5 ,4**

**Code: psychological stress due to child stubbornness**

**Number: 11**

**Code: Psychological stress affected caregiver’s health**

**Number:12, 2, 8 ,4**

**Code: positive impact of child disease on caregiver personality**

**Number: 1,5 ,7**

**Code: demanding husband**

**Number: 1**

**Code: unappreciative husband**

**Number:1**

**Code: caregiver scared to lose child**

**Number: 1, 2, 4**

**Code: caregiver adapted to child disease**

**Number: 10, 2**

**Code: caregiver became angry with children during child crisis**

**Number:10**

**Code: government not buying for marrow transplant**

**Number:10**

**Code: caregiver is fatigue during child admissions**

**Number: 1, 9, 16**

**Code: caregiver takes care despite her health condition**

**Number:1**

**Code: Disease has no effect on caregiver nor child social life**

**Number: 15, 16,**

**Code: Relationship with husband not affected due to child disease**

**Number: 15, 10, 16, 7**

**Code: caregiver cannot afford buying clothes**

**Number:1**

**Code: Caregiver attending less to family and friends’ gatherings**

**Number: 5,2, 10,12,1, 9**

**Code: poor financial state of caregiver**

**Number: 8, 1, 10 ,19**

**Code: financial aid from charities**

**Number: 8**

**Code: husband not supporting emotionally**

**Number:1**

**Code: patient is supporting the caregiver emotionally**

**Number:1**

**Code: Buying healthy food affects budget**

**Number: 12**

**Code: Buying food during admission affecting budget**

**Number: 11, 8, 1**

**Code: leaving work to take care of patient**

**Number: 9, 18, 19,7**

**Code: Caregiver receives financial aids from relatives**

**Number:2, 8, 12, 18**

**Code: caregiver always cry due feeling regrets**

**Number: 1 ,4**

**Code: Caregiver psychologically stressed during crisis**

**Number: 15, 1**

**Code: Weather control their outgoings with family & friends due to SCD**

**Number: 15, 7**

**Code: feeling regret being the reason of child disease**

**Number: 1, 13, 16**

**Code: Vacations cancelled due to SCD crisis**

**Number: 15, 5, 2, 19, 7**

**Code: Child’s experience with drug encouraged his affected caregiver to take it**

**Number: 15**

### 3- To understand caregivers’ perspectives regarding the challenges and problems of caregiving.

**Code: Caregiver refuse to give consent to strong opioid to child**

**Number: 9**

**Code: Caregiver overreacts in caring to child (strict, controlling & obsessive)**

**Number:9**

**Code: Caregiver do not think of having another child in future because of risk of SCD**

**Number: 13,**

**Code: Caregiver do not know that she is carrier otherwise she have chose the normal gene in IVF**

**Number: 14**

**Code: caregiver not aware of SCD**

**Number: 18,10**

**Code: Caregiver maintain healthy lifestyle to child**

**Number:12**

**Code: Caregiver maintain healthy diet to child**

**Number:12**

**Code: Lack of knowledge about side effects of SCD drugs**

**Number: 8**

**Code: caregiver well educated about SCD**

**Number:1**

**Code: Caregiver felt secure after hearing others' experiences with hydroxyurea**

**Number:15,7**

**Code: Caregiver was afraid of hydroxyurea**

**Number: 15, 7, 1 ,13**

**Code: Caregiver is worrisome about blood transfusions**

**Number:12**

**Code: Caregiver refuse to administer drugs to child**

**Number:12**

**Code: trust issues due to rumors about The hospital Salmaniya**

**Number: 18**

**Code: Patient not compliant to medication**

**Number: 18,**

**Code: patient not complaint due to hydroxyurea side effects**

**Number:8**

**----------------------------------------------------------------------------------------------------**

**Codes: Husband aware of SCD before marriage**

**Numbers: 11, 7, 15 (have SCD)**

**Code: Premarital test was normal**

**Number: 11, 5, 7, 4**

**Code: premarital test was not done (not mandatory)**

**Number: 1, 12, 3,8, 15,1, 14, 17,18 ,19**

**Code: Beta- thalassemia was not tested in premarital**

**Number: 12,9**

**Code: Crisis was due to trauma**

**Number: 11**

**Code: Adolescent not caring about his health**

**Number: 11, 18, 9,**

**Code: Child wishes death during crisis**

**Number: 11, 14, 2,1**

**Code: Hydroxyurea lessen the crisis frequency**

**Number: 15**

**Code: Caregiver suggest for ministry to provide radical solution for SCD**

**Number: 8**

**Code: no discrimination noted in health services**

**Number:1, 13,**

**Code: Initial care at home during crisis**

**Number: 11, 4, 12, 8, 10, 7, 17, 18, 13, 1,19**

**Code: First choice for caregiver is SMC for complicated cases**

**Number: 16**

**Code: Crisis during childhood was less in intensity and frequency**

**Number: 18**

**Code: nature of disease well understood by ministry of education**

**Number: 18**

**Code: caregiver not following up with doctors**

**Number:12**

**Code: Child gets sick when caregiver absent**

**Number: 12**

**Code: Pain during crisis make child angry**

**Number: 2, 12, 8**

**Code: child’s academic performance was affected.**

**Number:12,2, 8,10, 9, 19**

**Code: child’s academic performance was not affected.**

**Number:1, 18, 15, 4 ,7**

**Code: personality of child helps caregiver adaptation**

**Number: 8,5**

**Code: School staff in public school aware of SCD**

**Number: 15, 16**

**Code: Cooperative teachers in public school**

**Number: 15, 13**

**Code: Cooperative teachers in private school**

**Number: 14**

**Code: Strong personality of child**

**Number: 15,**

**Code: Child questioning mother if he has disability**

**Number: 15**

**Code: SCD affects child’s future ambitions**

**Number: 15, 5, 1, 19**

**Code: Caregiver does not prevent child from football**

**Number:15**

**Code: students and teachers need awareness about SCD**

**Number:1, 19, 7**

**Code: child play sport despite his disease**

**Number:1**

**Code: cvs sibling**

**Number:1**

**Code: after affected child mother’s tests relived beta-thalassemia carrier**

**Number: 10, 5, 9**

**Code: most crisis during exams**

**Number:10 ,17 ,4**

**Code: child accepted having the disease**

**Number: 10, 9, 16,7**

**Code: female leadership in public schools are more caring than male leadership.**

**Number: 1**

**Code: Teacher ask the patient to write a lot.**

**Number:1, 2**

**Code: bag is too heavy**

**Number:1**

**Code: Teachers threaten the patient if not doing his loaded homeworks will not pass .**

**Number:1**

**Code: Sports class is obligated although medical report was submitted**

**Number:1**

**Code: Students at school abuse the patient**

**Number:1**

**Code: Patient accuses the mother for his disease.**

**Number:1 ,4**

**Code: Caregiver save the stem cell of her child from the placenta**

**Number: 14**

**Code: Blood test was not clear & missed diagnosis (delayed diagnose).**

**Number: 9**

**Code: Crisis effect mentality of the patient**

**Number: 9**

**Code: Child rebellious because of control in diet & lifestyle**

**Number:9, 4**

**Code: Child affected mentally because of control**

**Number: 16**

**Code: Crisis happen during critical time in child**

**Number: 9**

**Code: Married despite positive premarital test for SCD**

**Number: 16, 15**

**Code: Caregiver controls child in diet & lifestyle**

**Number: 9, 16**

**Code: Private school has minimal knowledge about disease**

**Number: 16**

**Code: Private school was intolerant and required medical reports**

**Number: 16**

**Code: Private school teachers unnecessarily prevent normal activities to child**

**Number: 16**

**Code: No special care for the child’s circumstances (private school)**

**Number: 16**

**Code: premarital test was not accurate**

**Number: 7**

**Code: discrimination was noted in health services**

**Number: 4**

**Code: No chairs for accompanying person in wards**

**Number: 4,7, 17**

**Code: child not accepted having the disease**

**Number: 4**

**Code: child does not take the drug**

**Number: 4, 7**

**Code: school staff are ignorant**

**Number: 19**

**Code: caregiver blame the partner for the disease**

**Number: 4, 19,5**

**Code: caregiver not satisfied with staff attitude**

**Number: 19, 7**

**Code: Caregiver did not blame the partner for the disease**

**Number :7**

**Code: caregiver is ready to take off at anytime**

**Number :7**

# **Part 2: Sorting codes into categories**

### 1-To understand the healthcare access problems faced by caregivers while seeking care for their SCD patients.

**Hospital wards problems**

**Code: wards need maintenance**

**Number: 10**

**Code: no entertainment facilities in child wards**

**Number:1, 2**

**Code: no ward for SCD/ need wards or building for SCD patients**

**Number: 9, 16 ,4, 19**

**Code: Caregivers sleep on the floor in ward**

**Number: 16, 12, 7, 17**

**Code: no bed for caregiver in wards (causes back pain)**

**Number: 16, 15**

**Code: beds at ward sometimes broken**

**Number:1**

**Code: no separate wad for SCD children**

**Number:1**

**Code: crowded ward rooms**

**Number:1, 7**

**Code: Uncomfortable ward beds**

**Number: 8,1**

**Code: SCD patients need remote control beds**

**Number: 8**

**Code: SCD females need separate building**

**Number: 8,18**

**Code: Process to go to wards is easy & fast**

**Number: 16**

**Code: Long waiting hours for private room**

**Number: 16**

**Code: Patient cannot sleep quietly in public room**

**Number: 16**

**Code: Old medical facilities in ward**

**Number: 16**

**Code: Caregiver prefer private room because more comfortable**

**Number: 17, 18, 16, 7**

**Code: Fatigue due to no beds to accompanying person(ward)**

**Number: 11, 2, 5, 13, 10, 12,1, 14, 16, 4,19**

**Code: More chairs should be added to wards**

**Number: 13, 7**

**Code: No chairs for accompanying person in ER**

**Number: 11**

**Code: Easier admission to ward before**

**Number: 15,**

**Code: Child refuses admission to adult ward**

**Number: 11, 18**

**Code: discharge patient despite new symptoms noted by mother**

**Number:1**

**Code: visiting hours not restricted**

**Number: 1**

**Code: SMC were less conservative when it comes to treatment than in private clinic**

**Number:9**

**Dirty bathrooms**

**Code: bathrooms only cleaned in morning**

**Number: 10**

**Code: paper toilets and soap not always available**

**Number:1,4,19**

**Code: caregiver cleans wards bathrooms**

**Number:1**

**Code: Dirty bathrooms in wards**

**Number: 2, 12,8, 1,10 ,19, 4**

**Code: bathrooms are for everyone**

**Number: 1 ,10**

**Code: Fewer bathrooms compared to many patients**

**Number: 17, 12, 13, 19,2**

**Code: Bathrooms need 24hrs cleaning**

**Number:8, 17**

**Code: child dislike having shower in ward bathrooms**

**Number: 10**

**Shortage of medications**

**Code: No ursodcoxycholic acid in hospital (only in KSA)**

**Number: 1**

**Code: No Voltaren in hospital**

**Number: 11, 10, 1**

**Code: Drugs not always available**

**Number: 13, 1, 7**

**Health centres better/preference**

**Code: Health Center is better than hospital**

**Number: 18, 12, 17**

**Code: Health center respond faster than hospital**

**Number:12**

**Code: child cannot tolerate spicy hospital food**

**Number:1**

**Code: Child does not like hospital food**

**Number: 11, 8, 15,1,4 ,19, 7**

**Reaching hospital**

**Code: delay reaching ER to find someone taking care of siblings**

**Number:1, 7**

**Code: husband leave work to drive to hospital**

**Number: 1,4**

**Code: Ambulance refusal to deliver to the preferred hospital**

**Number:12**

**Code: Caregiver cannot drive during crisis due to panic**

**Number:12, 4**

**Code: Caregiver has private car**

**Number: 15, 10, 11,5, 9, 16**

**Code: car sometimes not available**

**Number:1**

**Code: Patient admitted fast to ER**

**Number: 9, 16**

**Code: insufficient parking lots in SMC**

**Numb: 18**

**Hospital staff**

**Code: Hospital staff are respectful and have good behavior**

**Number:15,1, 3, 14, 16, 7**

**Code: ER doctors are less experienced with sickle cell patients**

**Number: 9**

**Code: Doctors show up late in ER**

**Number: 9**

**Code: Nurses in ward ignorant**

**Number: 11**

**Code: Nurses in ER ignorant**

**Number: 8**

**Code: pediatrics doctors not available at adult ward**

**Number: 11, 19**

**Code: shortage in doctors and nurses in ER**

**Number: 11, 12, 9(nurses), 19 (nurses)**

**Code: Doctor cannot come to ER**

**Number:8**

**Code: Nurses less experienced in finding veins in child**

**Number:9, 8**

### 2- To assess the influence of being a caregiver on their own social, emotional, and financial lives.

**SOCIAL**

**Relationship with Husband**

**Code: relationship with husband affected due to child disease**

**Number:8,5 , 4**

**Code: recurrent child admissions affected relationship with husband**

**Number: 8**

**Code: husband married 2nd wife to have healthy children**

**Number:8**

**Code: husband do not visit child to offend mother**

**Number:8**

**Code: Relationship with husband not affected due to child disease**

**Number: 15, 10, 16, 7**

**Code: demanding husband**

**Number: 1**

**Code: unappreciative husband**

**Number:1**

**Code: Husband not helping in caring of child**

**Number: 11, 8,1, 4**

**Code: Husband nervous during crisis**

**Number: 18**

**Code: Husband helping in caring of child**

**Number: 10, 17, 16**

**Code: Husband provide emotional support to caregiver**

**Number: 18**

**Code: husband not supporting emotionally**

**Number:1**

**The effect of caring of SCD on other sibling**

**Code: Siblings jealous of sick child**

**Number: 11, 4, 15, 10, 4**

**Code: Siblings not jealous of sick child**

**CcNumber:12**

**Code: Child relationship with caregiver is stronger compared to his sibling due to the disease and more time**

**Number: 15, 9, 19**

**Code: Encouraging siblings to be independent to help them if mother absent to take care of patient**

**Number: 8**

**Code: siblings adapt to sister situation**

**Number:8**

**Family and friends - or - Effects on social life**

**Code: Child’s disease affects negatively on caregiver’s relationship with other family members**

**Number:9, 4**

**Code: Caregiver attending less to family and friends’ gatherings**

**Number: 5,2, 10,12,1, 9**

**Other**

**Code: Disease has no effect on caregiver nor child social life**

**Number: 15, 16,**

**Code: Dispersion of caregiver life due to SCD**

**Number:5, 8**

**Code: caregiver’s social life is affected**

**Number: 4**

**EMOTIONAL**

**Feeling Afraid**

**Code: Mother afraid of having a child with SCD**

**Number: 18 ,19, 4, 7**

**Code: caregiver scared to loss child**

**Number: 1, 2, 4**

**Regret feeling**

**Code: No regrets marrying carrier husband**

**Number: 11, 10**

**Code: feeling regrets marrying carrier husband**

**Number: 8, 19, 18**

**Code: caregiver always cry due feeling regrets**

**Number: 1, 4**

**Code: feeling regret being the reason of child disease**

**Number:1, 13, 16**

**Shocked - or - feeling after diagnosis**

**Code: Caregiver shocked after the child diagnosed with SCD**

**Number: 11, 14,10,9, 19**

**Code: After SCD diagnosis, father is more affected**

**Number:8, 14**

**Feeling Angry**

**Code: Caregiver is acting angry during crisis toward child**

**Number: 9**

**Code: caregiver became angry with children during child crisis**

**Number:10**

**Feeling Sorry**

**Code: Caregiver felt sorry for child.**

**Number: 9**

**Code: Caregiver apologize to child for having SCD**

**Number: 8, 14**

**Psychological stress**

**Code: Caregiver faced psychological stress from being in hospital**

**Number: 18,2, 16, 4, 19, 7**

**Code: psychological stress due to child stubbornness**

**Number: 11,**

**Code: Psychological stress affected caregiver’s health**

**Number:12, 2, 8 ,4**

**Code: caregiver is mentally distracted**

**Number: 1**

**Code: caregivers cannot give more**

**Number:4**

**Feeling Satisfied**

**Code: Caregivers feels their condition is better than others**

**Number: 12, 15, 18,17,11, 16, 4**

**Emotional support**

**Code: Husband provide emotional support to caregiver**

**Number: 18**

**Code: husband not supporting emotionally**

**Number:1**

**Code: patient is supporting the caregiver emotionally**

**Number:1**

**Code: Pray and read Quraan a lot during crisis**

**Number: 17,4**

**Express feelings**

**Code: Caregiver do not talk openly about her issues to people.**

**Number: 9, 16, 7**

**Code: Caregiver do not show her struggle to child**

**Number: 9**

**Positive impact on caregiver**

**Code: positive impact of child disease on caregiver personality**

**Number: 1,5**

**Code: caregiver adapted to child disease**

**Number: 10, 2**

**Code: Child’s experience with drug encouraged his affected caregiver to take it**

**Number: 15**

**Physical stress**

**Code: caregiver is fatigue during child admissions**

**Number: 1, 9, 16**

**Code: caregiver takes care despite her health condition**

**Number:1**

**Code: Fatigue due to lack of sleep**

**Number: 18, 1, 10, 5, 13, 14 ,4, 19, 7**

**Code: caregiver health is affected by the disease**

**Number:4**

**FININCIAL**

**No financial problem**

**Code: no financial problems**

**Number: 15, 13, 9, 16, 4**

**Have financial problem**

**Code: financial crises sometimes during admissions**

**Number: 10**

**Code: government not buying for marrow transplant**

**Number:10**

**Code: caregiver cannot afford buying clothes**

**Number:1**

**Code: poor financial state of caregiver**

**Number: 8, 1, 10 ,19**

**No effect on work**

**Code: Disease has no effect on work life for caregivers**

**Number: 15**

**Code: Work is tolerant with caregiver**

**Number:12, 1, 16**

**Effect on work**

**Code: caregiver work is affected because of the disease**

**Number: 4, 19, 5**

**Code: leaving work to take care of patient**

**Number: 9, 18, 19, 7**

**Code: Child admission caused mother to be absent**

**Number: 11, 5, 4**

**Code: Moving from full time job to part time job**

**Number: 17**

**Code: Work was not tolerant with caregiver’s absence**

**Number:9, 7**

**Expenses**

**Code: Petroleum affected budget during child admissions**

**Number: 8**

**Code: Buying food during admission affecting budget**

**Number: 11, 8, 1**

**Code: Buying medicines affects budget**

**Number:12,1**

**Code: Adolescent do not want the hospital’s Ibuprofen so caregiver buy it**

**Number: 18**

**Code: Buying healthy food affects budget**

**Number:12**

**Code: Vacations cancelled due to SCD crisis**

**Number: 15, 5,2, 19**

**Insurance**

**Code: Caregiver have health insurance**

**Number:14**

**Code: Caregiver has health insurance (private hospital)**

**Number: 16**

**Role of charities**

**Code: Refusal of Bahrain society of SCD to give financial support**

**Number: 8**

**Code: Ahlam Charity gave gifts**

**Number: 16**

**Code: refusal of charity to give financial support**

**Number:10, 3**

**Code: Child admission caused mother to be absent**

**Number: 11, 4, 5**

**Code: financial aid from charities**

**Number: 8**

**Financial Adaptation**

**Code: caregiver try to balance finance to prevent loaning**

**Number:10**

**Code: Caregiver receives financial aids from relatives**

**Number:2, 8, 12, 18**

**Code: loaning money to seek medical care**

**Number: 18**

**Code: Loaning to cover private room expenses**

**Number: 18**

**Code: Husband is not helping financially**

**Number:12**

### 3- To understand caregivers’ perspectives regarding the challenges and problems of caregiving.

**Caregiver facing disease with strength**

**Code: Caregiver do not show her struggle to child**

**Number: 9**

**Code: Caregiver do not talk openly about her issues to people.**

**Number: 9, 16, 7**

**Code: positive impact of child disease on caregiver personality**

**Number: 1,5**

**Code: caregiver adapted to child disease**

**Number: 10, 2**

**Code: caregiver takes care despite her health condition**

**Number:1**

**Code: Disease has no effect on caregiver nor child social life**

**Number: 15, 16,**

**Code: Relationship with husband not affected due to child disease**

**Number: 15, 10, 16, 7**

**Caregiver is aware of healthy lifestyle**

**Code: Caregiver maintain healthy lifestyle to child**

**Number:12, 4**

**Code: Caregiver maintain healthy diet to child**

**Number:12**

**Code: Caregiver well educated about SCD**

**Number:1**

**Code: Initial care at home during crisis**

**Number: 11, 4, 12, 8, 10, 7, 17, 18, 13, 1,19**

**Worrying about medication**

**Code: Caregiver refuse to give consent to strong opioid to child**

**Number: 9**

**Code: Caregiver felt secure after hearing others experience with hydroxyurea**

**Number:15,7**

**Code: Caregiver was afraid of hydroxyurea**

**Number: 15, 7, 1 ,13**

**Code: Caregiver is worrisome about blood transfusions**

**Number:12**

**Code: Caregiver refuse to administer drugs to child**

**Number:12**

**Code: trust issues due to rumors about the hospital Salmaniya**

**Number: 18**

**Code: Patient not compliant to medication**

**Number: 18**

**Code: patient not complaint due to hydroxyurea side effects**

**Number:8**

**Caregiver showing over restrictions**

**Code: Caregiver overreacts in caring to child (strict, controlling & obsessive)**

**Number:9**

**Lack of knowledge**

**Code: Caregiver do not know that she is carrier otherwise she has chosen the normal gene in IVF**

**Number: 14**

**Code: caregiver not aware of SCD**

**Number:  18,10**

**Code: Lack of knowledge about side effects of SCD drugs**

**Number: 8**

**Code: premarital test was not done (not mandatory)**

**Number: 1, 12, 3,8, 15,1, 14, 17,18**

**Code: Caregiver refuse to give consent to strong opioid to child**

**Number: 9**

**Caregiver fails to cope socially and emotionally**

**Code: caregivers cannot give more**

**Number:4**

**Code: caregiver health is affected by the disease**

**Number:4**

**Code: caregiver’s social life is affected**

**Number: 4**

**Code: caregiver work is affected because of the disease**

**Number: 4,19, 5**

**Code: caregiver is mentally distracted**

**Number: 1**

**Code: Dispersion of caregiver life due to SCD**

**Number:5, 8**

**Code: psychological stress due to child stubbornness**

**Number: 11**

# **Part 3: Themes write up**

### 1-To understand the healthcare access problems faced by caregivers while seeking care for their SCD patients.

| **Codes** | **Transcript Excerpt in Arabic** | **Categories** | **Themes** |
| --- | --- | --- | --- |
| **Code: paper toilets and soap not always available**  **Number:1,4,19** | ***Quote_IDI_4:***  *“مافي صابون ولا كلنكس محد يحب يدخله”*  ***Quote_IDI_1:***  *“ لان ما اقدر ادخل حتى الكلينس و الصابون مو دائما موجود "*  ***Quote_IDI_19:***  *"ما تحصلين الكلينكس التشو الصابون ما تحصلينه تغسلين ايدش هاي ما تحصلينه "* | **Lack of clean areas** | **Perceptions of poor facilities** |
| **Code: Dirty bathrooms in wards**  **Number:12, 1,10 ,19, 4, 8, 2, 7** | ***Quote_IDI_19:***  *"يبى ليه تركيز بالنظافة حتى الحمامات إذا تكلمينهم اللحين ما في حق تظيف الكلينر راح في الليل والتنظيف ينظفونه الصبح بس الصبح الحمامات تتنظف ما تتنظف على طول"*  ***Quote_IDI_10:***  *"* ***احنا نستكره ندخل الحمام يعني لين بندخل التسبوح الصبح بعدين ما نطيق اني صراحة ساعات اروح الحجرة الخاصة مفيها احد ادخل اتسبح فيها او يعني حتى لو شافوني مينطاق المكان وصخ حالته حالة"***  ***Quote_IDI_2:***  ***احس لو كل واحد له حمام احسن***  ***Quote_IDI_4:***  ***"الحمامات ماساة وهي ترفض تدخل حمام "***  ***Quote_IDI_1:***  ***"الحمام ماساه مثلا المرضى يحتاجون الى مكن معقم يعني شلون الحمامات عامه للمرضى و الزوار و المرافقين "***  ***Quote_IDI_12:***  *", بالنسبة لنظافة الجناح حتى لو ينظفون المحل اللي فيها, الداخل و الطالع لازم يتوصخ, دورات المياه الله يعزكم لازم تتوصخ, لأن دورات المياه قليلة و المرضة واجد"*  ***Quote_IDI_8:***  *الحمامات يعني من ناحية الكلينر الي يجون ينظفون هذيلا ما احسهم يهتمون وايد لأن على طول الحمام المفروض 24 ساعة يتنظف لأن يستخدم بالمرضى*  ***Quote_IDI_7:***  *و أحيانا لدرجه انج تشمين ريحه خاصا وقت الزيارات* |  |  |
| **Code: Fewer bathrooms compared to many patients**  **Number:2, 17, 12, 13 , 19** | ***Quote_IDI_17:***  ***“حمامات السلمانية يبغه لها زيادة وصيانة”***  ***لانه مثلا لما أترقد.. أني ما اترقد واجد.. بس اذا اترقد***  ***حمام واحد للجناح***  ***ساعات تلاحظين 12 مريضة على حمام واحد***  ***حتى في الطوارئ حمامين على ثلاث غرف غير الفيزيتور الي ويه المريض"***  ***Quote_IDI_13:***  ***"و صعب انه حمامين 2 بس موجودين في الجناح المرضى"***  ***Quote_IDI_2:***  ***“احس لو كل واحد له حمام احسن"***  ***Quote_IDI_19:***  ***"طالع واحد داخل يعني عشرين شخص على حمامين حمامين بس في الممر اهني ما تقدرين وعشرين شخص طالع واحد من غير الامهات عشرين شخص مرضى من غير الامهات موجودات من غير الزيارة إذا وحدة اجيه الحمام اني ما اخذ راحتي كلش ارد ارجع البيت اسبح وارد ارجع إلا إذا اني مضطرة ما اروح "***  ***Quote_IDI_12:***  *“بالنسبة لنظافة الجناح حتى لو ينظفون المحل اللي فيها, الداخل و الطالع لازم يتوصخ, دورات المياه الله يعزكم لازم تتوصخ, لأن دورات المياه قليلة و المرضة واجد"* |  |  |
| **Code: Bathrooms need 24hrs cleaning**  **Number:8, 17** | ***Quote_IDI_17:***  *“****حتى لو ينظفونهم على طول ما يغطون على عدد المرضى”***  ***Quote_IDI_8:***  ***“من ناحية الكلينر الي يجون ينظفون هذيلا ما احسهم يهتمون وايد لأن على طول الحمام المفروض 24 ساعة يتنظف لأن يستخدم بالمرضى"*** |  |  |
| **Code: no ward for SCD/ need wards or building for SCD patients**  **Number: 9, 16 ,4 ,19** | ***Quote_IDI_4:***  ***”عنهم قالوليي روحي الكبار هي 14 سنة وكانت تروح اطفال وهي 13 وانتي تعرفي عناية الاطفال غير عن عناية الكبار "***  ***Quote_IDI_4:***  ***"مرة رقدت في قسم الكسور هي لاعت جبدها***  ***Quote_IDI_4:***  ***"اللي وياها كله كبار ما وياها احد تاخذ وتعطي وياه فلكلام لكن لو اطفال تروح تكلمهم وتزاورهم ويطلبون لهم اكل ف انا ارتاح نفسيا بس اخر مرة لا كلهم كبار واجانب***  ***"***  ***Quote_IDI_9:***  ***يعني لازم يخلون ليهم قسم خاص يعني الحين العيادة أهناك هاي زينة ليهم لأن بسرعة يعالجونهم يعرفون ليهم مثلا .. واحد يجي مثلا أبسط الأمور ولدي أخر مرة رقدوه في قسم العيون الجناح ..***  ***Quote_IDI_16:***  ***"نحتاج جناح جديد , نحتاج مبنى بكبره جديد للسكلر***  ***"***  ***Quote_IDI_19:***  ***": المستشفى حاليا ما قاعد يتعدل المستشفى المرضى وايد السكلر يبى ليهمقسم جناح بروحهم"*** | **Needing of separate building for SCD patients** |  |
| **Code: no separate wad for SCD children**  **Number:1** | ***Quote_IDI_1:***  *”ااااه بعد ما في جناح خاص لمرضى السكلر لأنهم يحتجون أشياء خاصه يعني مثلا تخيلي ان واحد عنده نوبة قويه "* |  |  |
| **Code: SCD females need separate building**  **Number: 8,18** | ***Quote_IDI_18:***  *"أي لوزين يفتحون للبنات مكان خاص بدل ما يوزعونهم في الاجنحة لانه الغرفة الخاصة واجد عبء مالي"*  ***Quote_IDI_8:***  ***“مرضى مال السكلر عندهم الشباب و الاولاد يعني فئة الرجال و هاي عندهم مركز بروحهم يعني البنات إلى متى يضلون يعانون من هاي السالفة"*** |  |  |
| **Code: Child refuses admission to adult ward**  **Number: 11,4** | ***Quote_IDI_11:***  ***“هي لان موتها الجهال الصغار تعودت ان تترقد وي جهال تحب يعني الاطفال تحب تلاعبهم فصايرة متضايقة في قسم الكبار ما تبي يعني تترقد في قسم الكبار حتى تقول ليي ماما باروح اترقد"***  ***Quote_IDI_4:***  ***"ف دخلت يعني غصبا عنهم قالوليي روحي الكبار هي 14 سنة وكانت تروح اطفال وهي 13 وانتي تعرفي عناية الاطفال غير عن عناية الكبار فلما اكتشفت انها بتروح غرفة الاطفال تمت تصيح تقول ماما شلون انا بدخل معاهم"*** |  |  |
| **Code: no bed for caregiver in wards (causes back pain)**  **Number: 16, 15** | ***Quote_IDI_15:***  *“الأم ما عندها شي أتنام عليه جدي و أني أقعد على كرشي غير أتعب ظهري يتعب* *يعني و فهني أمبا أنام ما أحس النوم الكافي يعني"*  ***Quote_IDI_16:***  *" يعني أتخيلي الأم يا تنام على الكرسي يا تنام على الأرض****,*** *و مو بتقعد يوم يومين بتقعد مدة طويلة لا, خمس ثلاثة أربع أيام, و واجد صعب, يعني خصبا عليش أصيدش أصلا إرهاق بدني****."*** | **lack of beds for caregiver in wards during child admissions leads to physical problems** |  |
| **Code: Fatigue due to no beds to accompanying person(ward)**  **Number: 11, 2, 5, 13, 10,1, 14, 16, 4,19, 15** | ***Quote_IDI_13:***  *"أنام وياها على نفس السرير .. مو مريح بس بعد شنسوي "*  ***Quote_IDI_15:***  *"الأم ما عندها شي أتنام عليه جدي و أني أقعد على كرشي غير أتعب ظهري يتعب* *يعني و فهني أمبا أنام ما أحس النوم الكافي يعني"*  ***Quote_IDI_16:***  *"يصيدني ألم في ظهري, يعني أني* ***already*** *عندي من قبل شوي ألم غزنة في الظهر فلما أجي أهني يزيد****"***  ***Quote_IDI_11:***  ***"كان قبل اقعد و هي صغيرة اتعب واجد نفسياً واجد واجد اتعب حتى جسدياً لان تعرفين هني بس هي بتنام فوق السرير اني ما اقدر انام فوق السرير انام على الارض"***  ***Quote_IDI_14:***  *"ما في يعني موفرين ليش شي عشان يعني يرتاح وينام المفروض يخلون على الاقل صوفا كحل"*  ***Quote_IDI_10:***  ***"مفي مكان انام وياه فوق السرير.***  ***المفابلة: وياه فوق السرير؟***  ***اي مفي مكان يعني انتين شفتين الحين الحجرة يعني حده المفروض يعني ادا هدا يحطون الينا كرسي يعني هو يتعب و اني اتعب "***  ***Quote_IDI_2:***  ***"المفابلة: هل مثلا صادج مشاكل بدنية بسبب القعدة معاها؟***  ***أي مشاكل في الظهر"***    ***Quote_IDI_5:***  ***"المقابلة: اوك مرافق هل المكان مهيا؟؟***  ***اذا خاص أي مهيا لكن لو عام لا***  ***السرير هو المشكلة وما كان في كراسي أصلا ممرات احط شيت وارقد فترة وهو صغير انام معاه علسرير لكن لما كبر ما ااقدر***  ***الشي الثاني ما نرقد من صريخ الأطفال***  ***الواحد ما يرقد هني!***  ***Quote_IDI_19:***  ***"الليل كنت امبي سرير وكنت تعبانه"***  ***Quote_IDI_4:***  ***"مثلا لو سريير فاضي تقولي نامي عليه واحانا ممرضات ما يرضون ف اضطر يا كرسي او سريرها اذا كان عريض واحيانا الممرضة تجيب لي اسفنج انام عليه"***  ***Quote_IDI_1:***  ***"احتاج سرير انام عليه ما يصير ان اهو يتألم في ظهره و انام معاه على نفس السرير و خصوصا ان كبر اللحين"*** |  |  |
| **Code: Caregivers sleep on the floor in ward**  **Number: 16,17, 7,12** | ***Quote_IDI_17:***  *"****وفي الجناح العام مافي كرسي للمرافق فنقعد على الأرض ونجيب فوط نحط لينه وننبطح.."***  ***Quote_IDI_16:***  ***"في الحجر المشتركة ما في سرير إليش , يعني يا أن تقعدين على كرسي, يعني حتى النومة في بعض الأمهات ينامون على الأرض, يحطون ليهم برنص و ينامون على الأرض , يعني يا دا"***  ***Quote_IDI_7:***  ***"الأرض كنت ارقد عل ارض"***  ***Quote_IDI_12:***  ***"بالذات لأنهم قسم أطفال لازم ولية الأمر وياه تحتاج مكان أن ترتاح وياه سرير أو شي, يعني أحس المكان إذا هو يترقد يترقد فترة الطويلة , في الفترة اللي طافت أترقد 18 يوم, 18 يوم أني أنام وياه على الأرض"*** |  |  |
| **Code: Uncomfortable ward beds**  **Number: 8, 1** | ***Quote_IDI_8:***  *“****الأسرة بشكل عام يعني مال المرضى مو مريحين"***  ***Quote_IDI_1:***  ***لاسر مالت المريض مو مريح او يكون على صوب نازل تحسين انج بتطيحين و كذا مره يشتكيلي من عدم راحه السرير*** | **Beds for patients in ER and wards are uncomfortable** |  |
| **Code: ER beds are uncomfortable**  **Number:12, 8** | ***Quote_IDI_8:***  *“****اي لأن اني اقولش بتي يعني مثلاً تدخل المستشفى يعني مثلاً ايدها ينتقل لظهرها تقول لي حتى من قعدتها على السرير يسبب ليها لأن ما ترتاح عليه هاي بعد سبب لينقل ليها المرض يعني حتى الجلسات الي مي عدلة المشية الي مي عدلة اليها نومتها الي مو عدلة"***  ***Quote_IDI_12:***  ***"تعرفين سرير الطوارئ مو مثل سرير فوق مريح و هو جسمه يعوره يبغى ليه مكان زين يعني, سرير عدل مو سرير صنجته نازلة"*** |  |  |
| **Code: Waiting in ER (hours -days) until referring to ward**  **Number : 11, 18, 8, 13,14,1, 12, 15, 9 ,19, 16** | ***Quote_IDI_18:***  *“اخر مرة االلعام شهر 3 طلعا من بيتنا ف الشمالية.. نزلني ابوها عشان نسوي الإجراءات وضلينا فترة اسرة ما في ساعتين يمكن بس...وتالي زوجي شاف احد يعرفه طرش لها سرير اسعاف وخلوها تنبطح عليه……..فطرشوني لرئيس الأطباء قال لي مو ايدي كانت تتالم واني صوبها ما بيدي شي، وتالي الدكتورة يوم ثاني جت الصبح تقول ما حصلو لش مكان؟ قلت لهم ما حصلوا سرير حق يحصلون مكان*  *فتالي عقب شوي جو قالو حصلنا غرفة خاصة في السادس الطابق السادس"*  ***Quote_IDI_13:***  *"أخر مرة لا اللي قبلها ظلينا يوم ونص في الطوارئ على ما يحصلون سرير"*  ***Quote_IDI_14:***  *" لين ما يحصلون له سرير فمثلا هالمرة من عشر وياه إلى العصر تقريبا الساعة 2 -3 اربع خمس ساعات في الطوارئ*  *"*  ***Quote_IDI_15:***  *المقابلة: كم ساعة أستغرق؟*  *يعني قولي اهناك من 10 يعني 3 و نص جت السستر قالت يلا عشان أنروح فوق , بس جابونا الجناح و تيسرت أمورنا الحمدالله*  ***Quote_IDI_8:***  *"****من ندخل الطوارئ نضل تقريباً 6 أيام تقريباً هالرة 8 أيام احنا في الطوارئ بسبب ان مافي سريرإليها"***  ***Quote_IDI_11:***  ***“تضل ليوم ثاني في الطوارئ مادري يعني ما يودونها الجناح ساعات يومين ثلاثة بعد اتم قبل ما كان جدي الوضع الوضع مأساوي صاير كلش"***  ***Quote_IDI_19:***  ***": هل الاسرة متوفرة إذا تجين***  ***والده المريض : لا احيانا نظل يوم كامل تحت***  ***"***  ***Quote_IDI_1:***  ***"الصراحة الصراحة اخر قرار اخذه أنى اجي المستشفى لان يكون تعب وارهاق مو طبيعي الي و اله مثلا في الطوارئ واجد يتاخر***  ***تقريبا اشقد تنتظرون؟***  ***وايد يتاخرون لدرجه ان اخر مره عطيته جرعه اكبر من البيت لان اعرف انهم يتأخرون فاحنا جينا"***  ***Quote_IDI_12:***  ***"أي عادي نقعد ثلاث ساعات بيرقدونه. عادي أنتظر ساعتين على ما يجيبونه فوق "***  ***Quote_IDI_16:***  ***"فاحنا ساعات أنتم طول اليوم من 5 إلى 6 ساعات ننطر نتيجة الدم, بعدها نرد ننطر في حدود 5 إلى 6 ساعات عشان يحصلون سرير أو يحصلون لينا private room"***  ***Quote_IDI_9:***  ***يعني دكاترة مال سكلر كلهم مو موجودين أصلا .. صايرين كلا في العيادة له ؟ مالت السكلر فما يعرفون ليه عدل و ما يجون بشكل سريع أصلا. يعني هو أتنقع ليلة كاملة في الطوارئ*** | **Long waiting hours in ER although there are no chairs for caregivers** |  |
| **Code: Waiting in ER till medication intervention**  **Number: 11, 2, 18, 10, 17, 8** | ***Quote_IDI_18:***  *“وهي تتالم سرير مافي ودكتور مافي نضل تقريبا ساعة ساعتين"*  ***Quote_IDI_17:***  *"****واجد ننتظر عادي نضل ساعة ونصف لأنه ما يدخلونه على انه حالة طارئة"***  ***Quote_IDI_8:***  *"****احنا دائماً مال التمريض مال السكلر قسم دي و ادا تتصل هني في سرير اوكي اذا مافي سرير خلاص يعني انتظري من تقريباً أقلها أقلها من 4 ساعات إلى 6 ساعات هذا احنا ننتظره، من بعد ال 6 ساعات يشوفها الدكتور حصلنا سرير خلاص"***  ***Quote_IDI_11:***  ***"اييي (نبرة حادة) تمت ساعتين بدون دوا ادوية مافي قالت ليي قمت اصارخ "تعالوا تعالوا تعبانو تعالوا ليي تعبانة" و هي يعني مو اني الي جبتها جابتها امي و اخويي و تنادي و تنادي و متى يقول لش جووا ليها عقب ساعتين جووا ليها ركبوا عليها السلان (سكوت)"***  ***Quote_IDI_10:***  ***"ننتظر سوع و لمن ندخل بعد الهدي الي يدخلونه حجرة الاطفال بعد بالسوع ننتظر علمن يجون اليه يعني مو سيدة يجون يعطونه الادوية"***  ***Quote_IDI_2:***  ***"يعني اذا تبي ابرة انتظري الى الساعة اللي عطوج إياها احنا امس من 11 الى 4 اليوم توهوم يعطونها الابرة اهي تعبت تعبت اني ما هقيتها تعيش هقيتها بتموت يعني خلاص*** |  |  |
| **Code: No voltaren in hospital**  **Number: 11, 10, 1** | ***Quote_IDI_11:***  *“****الادوية بعد نشتري ليها الحين (تضحك بصوت عالي) الحين نفس الفولترين ما عندهم في المستشفى مجمع وش كبره ما عندهم ادوية فولترين ما عندهم فاشتريت ليها فولترين و جبته ليها كل مرة جدي يعني"***  ***Quote_IDI_10:***  ***"يعطونا ادوية بس هاي الفولترين يعني ساعات هاي على طول احنا نشتريه هاي مال المسوح"***  ***Quote_IDI_1:***  ***"أي بس مو دايما نحصله بعض الاحبان يكون مو متوفر"*** | **Shortage of medications** |  |
| **Code: Drugs not always available**  **Number: 13 , 1, 7** | ***Quote_IDI_13:***  *"الأدوية ساعات موجودة وساعات نجيبها من البيت...في بعضهم نشتريها وفي بعض مثلا التحميلات ساعات يعطونها وساعات ما عندهم يكونون نشتريها بس الادوية الباقي كله من عندي"*  ***Quote_IDI_1:***  *"في عندي مشكله اللحين كبيره 3 مرات خلال سنتين يعطوني اسم دوا و ان يبغوني اوفره و الدوا امو متوفر في البحرينursodcoxycholic acid*  *"*  ***Quote_IDI_7:***  *"كان قبل متوفر بس صرلنا فتره ما كان متوفر"* |  |  |
| **Code: Crowded ER rooms**  **Number: 8** | ***Quote_IDI_8:***  *“****تصوري الحجرة الي هم قاعدين في غرفة دي روحوا شوفوها مأساة تقريباً 14 مريض"*** | **Small places for SCD with high number of patients** |  |
| **Code: tiny place for SCD patients in ER**  **Number: 8** | ***Quote_IDI_8:***  *“****بالنسبة إلى مكان المرضى مال السكلر جداً جداً يعني صغير ضيق عليهم ما يستوعب العدد الي هم فيه يعني كثر ما هم يترددون على الطوارئ"*** |  |  |
| **Code: crowded ward rooms**  **Number:1 , 7** | ***Quote_IDI_1:***  *“مثلا تخيلي ان واحد عنده نوبة قويه و مو نايم كذا يوم و الغرفة فيها 6 مرضى يعني ولو كان مريضين او 3 بالكثير لانهم بعد أطفال و يصيجون في وقت مختلف ف 6 اشخاص وايد و م في أي راحه"*  ***Quote_IDI_7:***  *"المكان واجد فضوى يعني في 6 اشخاص في الغرفه غير الزوار و لدي يكون تعبان و مو قادر يرتاح"* |  |  |
| **Code: Patient cannot sleep quietly in public room**  **Number: 16** | ***Quote_IDI_16:***  *“احنا فيها 6 مرضى و أكثرهم يكونون سكلر, فتلاقي طول اليوم عندهم نوبات ألم, صعب النوم, صعب .."* |  |  |
| **Code: Child does not like hospital food**  **Number: 11, 8, 15,1,4 ,19 ,7** | ***Quote_IDI_8:***    *“****اكلها ما تقدر تاكل اكل من المستشفى تقطه و لا نقدر مثلاً اني ان اكلم الادارة هنيها ان لا ييبون ليها اكل تعرفين اكل المستشفى مو الكل يرغب فيه و على ما يوصل يكون بارد انزين و في بارسلات و نوعيته ما يعني هي عموماً ما تاكل اكل المستشفى"***  ***Quote_IDI_11:***  ***"هي ما تبي من اكلهم عشيات ما يعجبها اكلهم ما يعجبها اقول ليها هدا انظف حتى من المطاعم الي بتاكلينها من برا على الاقل هدا نضيف مستشفى و انتين تاكلين اكل هنود مال برا ما تبي اكلهم يعني نفس البارحة ما اكلت منهم"***  ***Quote_IDI_19:***  ***" 11 يوم بيض وجبن بيض وجبن واله اني لاعت جبدي"***  ***Quote_IDI_7:***  ***"ما كنت اكل و لا انا و لا ولدي من اكل المستشفى و كان زوجي يوفر الاكل لنا "***  ***Quote_IDI_1:***  ***1"الصراحه الاكل وايد مكلف بالنسبه لي لان ما يتقبل اكل المستشفى***  ***و شنو السبب ؟***  ***لان طعمه مو حلو و بعد ما في تنوع "***  ***Quote_IDI_4:***  ***"الام تقول ماصخ ومو مساوي عدل ف اضطر اني اجيب ليها"***  ***Quote_IDI_15:***  ***"الريوق ! أيجيبون لينا بيض مفيوح يسدون نفسنا من الصبح ههه (ضحكة) محمد ما يحب , ما يحب حتى في البيت يعني ما يعني قليل أني ما أتقبله"*** | **SCD patients do not like Hospital food that served during admissions** |  |
| **code: no chairs for accompanying person in ER**  **number: 11** | ***Quote_IDI_11:***  *"****في الطوارئ مافي حتى كراسي تقعدين عليها تميت اني كل وافقة و اني ما اقدر اوقف واجد قلت ليها ما اقديرتاح و بس في كرسي واحد للمرافق لما كان صغير كنت ارقد جمبه لان يكون مثلا مون ايمه أسبوع اة 10 أيام و أكون جدا مرهقهر اوقف ماما بمشي عنش قبل في كراسي الحين حتى كراسي مافي في الطوارئ"*** | **lack of comfortable chairs** |  |
| **code: chairs uncomfortable for caregiver in ER**  **number: 16** | ***Quote_IDI_16:***  ***: يكفي الجاهل يعني تنتظرين 6 7 ساعات و أنتين قاعدة على كرسي بلاستيك جدي سيدة واقفة ظهرش يعني ما أطلعين إلا ظهرش خلاص أنكسر من كثر القعدة*** |  |  |
| **code: no chairs for accompanying person in wards**  **number: 4,7, 17** | ***Quote_IDI_7:***  *"يرتاح و بس في كرسي واحد للمرافق لما كان صغير كنت ارقد جمبه لان يكون مثلا مون ايمه أسبوع اة 10 أيام و أكون جدا مرهقه*  ***Quote_IDI_4:***  *"اذا تبين توقفين مافي كرسي تضلين واقفة "*  ***Quote_IDI_17:***  *وفي الجناح العام مافي كرسي للمرافق فنقعد على الأرض ونجيب فوط نحط لينه وننبحط..* |  |  |
| **code: more chairs must be added to ward**  **number: 13 , 7** | ***Quote_IDI_13:***  *"يعني والكراسي*  *متوفرة شوية يعني علشان لو بيجون زوار انه في كراسي زيادة "*  ***Quote_IDI_7:***  *“يرتاح و بس في كرسي واحد للمرافق لما كان صغير كنت ارقد جمبه لان يكون مثلا مون ايمه أسبوع اة 10 أيام و أكون جدا مرهقه "* |  |  |

| **Themes** | **Category** | **Transcript Excerpt in Arabic** | **Code** |
| --- | --- | --- | --- |
| **Lack of satisfaction with services provided during visit** | **Caregivers prefer health centers services than hospitals** | ***Quote_IDI_18:***  *"والحين تحولنه على مركز جابر الصباح واجد احسن أوسع واشرح"*  ***Quote_IDI_17:***  *"المقابلة: وين أول مكان تروحونه اذا تعب؟*  ***المركز***  *المقابلة: ليش تفضلون المركز؟*  ***من تجربة لاحظت أنه .. من المشكال الي صادتني مع ولدي الكبير فما ابغي انه اعيد التجربة"***  ***Quote_IDI_12:***  *: , فلو وديته للمركز الصحي , في مركز الصحي أحس إهتمامه أحسن من أهني, أن من أوصل سيدة يدخلونه , علا طول يجيبون له دكتور و يعالجه أهني أجي أنتظر بالثلاث ساعات* | **Code: Health Center is better than hospital**  **Number: 18, 12, 17** |
|  |  | ***Quote_IDI_12:***  *: , فلو وديته للمركز الصحي , في مركز الصحي أحس إهتمامه أحسن من أهني, أن من أوصل سيدة يدخلونه , علا طول يجيبون له دكتور و يعالجه أهني أجي أنتظر بالثلاث ساعات* | **Code: Health center respond faster than hospital**  **Number: 12** |
|  | **Good attitude towards caregivers** | ***Quote_IDI_14:***  *المقابلة: شلون معاملة الطاقة الطبي الدكاترة الممرضات*  *ام المريضة: معاملتهم زينة يعني اني ارتاح صراحة"*  ***Quote_IDI_15:***  *المقابلة: أوكي و يوم رحتي الطوارئ طبعا شلون كانت التعامل مع الممرضات و الدكاترة؟*  *أم المريضة: لا لا معاملة ممتازة يعني ما يحتقرون لو يعاملون معاملة لأ , يعني يستقبلون أوكيه*  ***Quote_IDI_16:***  *:بس كرعاية صحية للأمانة لا في إهتمام في متابعة, النرسات 31 ما قصرو يعني, دائما يكونون بشوشين دائما إذا اتقولين ليهم شي ما يقصرون, الدكاترة نفس الشي"*  ***Quote_IDI_7:***  *: أي الصراحع دكتوره عال العال أوقف لها احترام يعني انا دخلت المستشفلى بس هذي غير و متفانبه في خدمتها حتى لو اطلع بره ما احصل نفسها الدكتوره (اسم الدكتورة)"*  ***Quote_IDI_1:***  *"الصراحة ما في شيء ما عليهم شيء بالعكس دايما يتوددون له و يعتبرونه ابنهم حتى اذا رفض يأخذون منه دم يتمون يتحايلون عليهم "*  ***Quote_IDI_3:***  *لاا بالعكس يعني مرضى السكلر الهم عناية خاصة.. يعني.. بسرعة نتلقى العلاج مافي تأخير* | **Code: Hospital staff are respectful and have good behavior**  **Number:15,1, 3, 14,16,7** |
|  | **Hospital staff are few and not always available** | ***Quote_IDI_11:***  *"****هي صارت في قسم ال يعني صارت وي الكبار بس ويش الي صار كلما اسأل دكتور قال انا مو مسؤول انا مو مال اطفال فهي مي معتبرينها انزين ادا انتون معتبرينها من الاطفال انزين ليش مخلينها وي الكبار؟ ليش مو مودينها وي جناح مال اطفال؟ يعني كلما اسأل دكتور قاللا هدي مو من صوبي هدي مو من صوبي كلما أسأل دكتور قال مو من صوبي بجي دكتور اطفال"***  ***Quote_IDI_19:***  ***"سرير حتى العلاج وهم كبار ما يحصلون اني اتمنى الدكتورة (اسم الدكتورة) حتى للكبار انه هي تواصل وياهم لحتى وهم كبار اما دكاترة للكبار مافيه احس انهم ضايعين "*** | **Code: pediatrics doctors not available at adult ward**  **Number: 11 , 19** |
|  |  | ***Quote_IDI_9:***  *المشكلة ما تقدرين تحكيم على الستاف مال الطوارئ يعني بجيف ممرضتين على ألف مئة , أني مو من النوع اللي أحكم على الشيء بعد .. يعني الخدمات جديه , مو لأن هي جت عاملت مثلا ولدي أو لأن ما سوت له خدمة لأن المشكلة كذا مريض, هذي الممرضة أدور لمن و تخلي من و كل واحد أخس من الثاني و كل .. أني ما أحكم عليهم بس هو كعدد ستاف هو قليل أكيد* ***..*** *دكاترة و على ما يجون يطلعون*  ***Quote_IDI_11:***  ***هو طوارئ لازم يكون فيه ممرضات اكثر و فيه دكاترة اكثر دكتور واحد او دكتورين يعني انتين لو دشيتين و شفتين الطابور الي واقفين الي يعني حق يسألون عن ولادهم و هو دكتور واحد الحين المفروض على الاقل 4 دكاترة حق قسم وش كبره فيه كل مريض يعني المفروض في عناية اكثر من جدي ما احس مافي عناية في الطوارئ كلش"***  ***Quote_IDI_12:***  *المقابلة:يعني أتحسي أن في الطوارئ هناك ما في مثلا , هني ..هناك نقص في الممرضات?*  *أم المريضة: أي نقص في الممرضات*  ***Quote_IDI_19:***  *"ثلاثة طاقم ما تستوعب وهو مساعدتهم للمرضات شوي وإذا تسألينهم انتظري شوي"* | **Code: shortage in doctors and nurses in ER**  **Number : 11, 12, 9(nurses),19(nurses))** |
|  | **Lack of experienced nurses and ER doctors** | ***Quote_IDI_9:***  *:يعني حليوين الممرضات و يحبون سلمان و بالعكس يمزحون وياه و يتقشمرون وياه بس ما يعرفون يسحبون دم منه , ذبجوه ذباح مسكين و هو يعني ساعات أول مرة أجوفه يصرخ*  ***Quote_IDI_8:***  *"****الحين تعرفين من بعد كثرة السيلانات و هاي العروق نفسها نعاني منها إن هي ما تنوجد تكون كلش ضامرة ضعيفة غصب إن يعني ينحط فيها السيلان يعني عدهم البنفسجي و الأصفر و المادري شلون الأضعف شي كلش ياخذونه الظاهر البنفسجي فهني يعني مو كل ممرضة تقدر تحط إليها يضطرون إن ينادون على دكتور، هذا طبعاً مو من البداية من البداية يمكن يحصلون إليها عرق "*** | **Code: Nurses less experienced in finding veins in child**  **Number:9, 8** |
|  |  | ***Quote_IDI_9:***  *المشكلة أهني الحين لحد عمر 18 سنة يستقبولونه في الطوارئ. و خبرش الطوارئ مأسى ما يعرفون أولا للحين يعني . يعني دكاترة مال سكلر كلهم مو موجودين أصلا .. صايرين كلا في العيادة له ؟ مالت السكلر فما يعرفون ليه عدل و ما يجون بشكل سريع أصلا.* | **Code: ER doctors are less experienced with sickle cell patients**  **Number: 9** |
|  | **Variable opinions regarding private sector** | ***Quote_IDI_16:***  *:يعني ساعات أني ألجأ إلى المستشفيات الخاصة قبل ما أجيبه علشان أتأكد من نتيجة دمه و أتأكد من نسبة تكسر الدم , ليش لأن في مستشفى الحكومة واجد يأخذ وقت و يمكن هو ما يحتاج ترقيد, نسبة تكسر الدم مو واجد و ال Hb مو واجد الهيموجلوبين نازل فما يحتاج ترقيد, فأني أوفر على نفسي إنتظار ال 6 لو 7 ساعات أروح مستشفى خاص,* | **Code: Laboratory test results are faster in Private hospitals**  **Number:16** |
|  |  | ***Quote_IDI_9:***  *: يعبترها الظاهر إستشارة بس من مجرد أزيد السؤال يزيد المبلغ بس يعني أحس مبالغة أن أحنا أنجي أهني و أورلدي سلمانية أتسوي جدي و أورلدي المراكز أتسوي جدي ليش أنخسر* | **Code: Private clinic is expensive and not different in quality than public hospitals**  **Number: 9** |
|  |  | ***Quote_IDI_16:***  *:المدرسة ما قاعدين يراعونه يقللون كمية الأشياء اللي المفروض يدرسها, ما يعطونه عناية خاصة أنهو سكلر يحتاج بيكاب, يحتاج تخفيف ال الmaterial مالت الدراسة فهذي المشكلة اللي واجهتني* | **Code: No special care for the child’s circumstances in private school**  **Number: 16** |

### 2- To assess the influence of being a caregiver on their own social, emotional, and financial lives.

### 3- To understand caregivers’ perspectives regarding the challenges and problems of caregiving.

| **Codes** | **Transcript Excerpt in Arabic** | **Category** | **Themes** |
| --- | --- | --- | --- |
| **Code: relationship with husband affected due to child disease**  **Number:8, 4, 5** | ***Quote_IDI_8:***  *"*  ***هو راح يكلم اهلي ان اني تغيرت بمجرد ما اني يبت (اسم المريضة) و حملت بها و بعديت تغيرت بزود لما عرفت ان هي مرضة سكلر فهني هو تغير عليي"***  ***Quote_IDI_4:***  ***"اي وقت اللي اهي تمرض اعصابي تفلت على ابوها واختها وهم يعرفوني ويعذروني اجواء البيت تصير كريهه كلش***  ***"***  ***Quote_IDI_5:***  ***"الحمدلله زوجي كان متفهم ومتعاونين لكن في البداية اخذنا اللوم مع بعض"*** | **The effect of caregiving on relationship with husband** | **The impact of caregiving on social life** |
| **Code: Relationship with husband not affected due to child disease**  **Number: 15, 10, 16, 7** | ***Quote_IDI_10:***  *"* ***المقابلة: انزين الحين علاقتش مع زوجش***  ***تحسين ان هي خنقول تدهورت او اختربت بسبب المرض؟***  ***أم المريض: لا الحمدلله مافي شي تغير يعني هو متقبل الوضع و اني متقبلة الوضع و الحمدلله"***  ***Quote_IDI_15:***  ***:المقابلة: أوكي في قط مرة مثلا مرض مال محمد أثر مع علاقتش مع زوجش أو مع عائلتش أو أصدقائش؟***  ***الأم: لا لا لا لا***  ***Quote_IDI_16:***  ***:المقابلة: شلون أثرت اللهو حالته المرضية مع علاقتش مع عائلتش مع زوجش مع أصدقائش هل أثر يعني؟***  ***الأم: (حركت رأسها بلأ)***  ***Quote_IDI_7:***  ***لا ما اثر عليها في دعم منه و متفهم هالشي*** |  |  |
| **Code: Husband not helping in caring of child**  **Number: 11, 8, 1, 4** | ***Quote_IDI_8:***  ***ابوها شقولش يعني (سكوت) ما عنده اهتمام في هالسالفة كل دايماً يتحجج "***  ***Quote_IDI_11:***  ***"لا بروحي هو ما يساعدني ما يساعدني بروحي يعني مو من النوع الي هو يعني يعني يجي يجي اليها واجد نفسي يعني هو يجي ليها مرة وحدة بس دقيقتين و مشى الاخ"***  ***Quote_IDI_4:***  ***"اي اكير واني اكثر من ابوها هو يشتغل احس مرات فلسلمانية هلعبئ مو اني اللي اتحمله يبيله رجال بس ابوها مايقدر يقعر فترات طويلة غيري اني مستحيل ارجع انتي بعد تشوفي دايما فلسلمانية دايما الام العبئ على الام"***  ***Quote_IDI_1:***  ***"من يشوفه يتالم خلاص ما يقدر يقعد معاه و يروح"*** |  |  |
| **Code: Husband helping in caring of child**  **Number: 10, 17, 16** | ***Quote_IDI_17:***  *"* ***يمكن اني االي مساعدني انه ابوهم زين وياي.. فشوي هذا يعني هذا يساعدني"***  ***Quote_IDI_16:***  ***:بيني و بين أبوه لأن أبوه دوامه شفتات فأبوه يعني ما يتواجد في البيت إذا بس يومين نهار فيتمون روحهم لين ما يجي من الشغل, و already هو يكون في المدرسة للساعة 3 بس يعني***  ***Quote_IDI_10:***  ***"لا نتعاون يعني هو في الترقيد شي طبيعي ان بصير عليي اني بس المواعيد يعني هو يجي ويايي اثنينا نجيبيه"*** |  |  |
| **Code: Siblings jealous of sick child**  **Number: 11, 4, 15, 10 ,7** | ***Quote_IDI_11:***  ***اي ساعات يعني مثلاً عندش علي ولدي الصغير يقول ليي "تخلينا و تروحين ليها؟" يعني تخلينا يعني يعني اروح ليهم جدي تقريباً تسع عشر يقولون "احنا ما تعشينا تخلينا و تروحين لبتش تقعدين وياها و احنا تخلينا بروحنا ما ايانا احد" يعني كأنه مثل الغيران منها ان اني اروح لاخته و اخليه"***  ***Quote_IDI_15:***  ***المقابلة:شنو علاقة الأخو مع الهادا؟***  ***الأم: الحمدالله بس فيه الشوي يعني ..***  ***المقابلة: غيرة؟***  ***الأم: الكبير يقول يعني قلت ليه أنزين هو الحين لأن مو عشان أفرق بينكم , يعني اللي عندي أعطيك أعطيه أذا تبى شي أسوي ليك و .. بس أنت كبير و هو صغير و محتاج عناية, و أنت تعرف مرضه بس هذا هو, و تهتمون فيه أكثر قلت ليه ما نهتم فيه أكثر أحنا أنسوي ليك يعني بس يعني كون مرضه يحكم***  ***Quote_IDI_10:***  ***" المقابلة: تحسين ان مرضه خلاش تميلين له اكثر منهم؟***  ***الأم: هم عندهم جدي يعني اني عندي ان كامل مثل الشي فهم يقولون ليي يعني تفرقين"***  ***Quote_IDI_4:***  ***"اي اختها تقول انتو كله مع شهد وجدي اقولها ماما هي يبي لها عناية خاصة***  ***بس ساعات ابوها ما يعصب على شهد يعصب على زينب اقولها انتي تعاندين خصوصا وقت الدراسة تقول كله علي اني اني***  ***تحس في مقارنه نفضلها عليها"***  ***Quote_IDI_7:***  ***"تخاف من ان اخوها يمرض لان تدري اني بهدها و حالتها واجد تذبل حتى للحين متاثه"***  ***"تقولي بنفسها كل الأمهات يجون يحضنون عيالهم في الروضه بس انا انتظرج و انتي ما تيين و كان هذا الشي واجد يأثر فيني"*** | **The effect of caregiving on relationship with other sibling** |  |
| **Code: Caregiver attending less to family and friends’ gatherings**  **Number: 5,2, 10,12,1, 9** | ***Quote_IDI_5:***  *'هههه أي وايد سفرات مناسبات اجازات ما طلعت يوم مفتوح بنتي العودة وتكريمها ان اهي متفوقة وايد اشيا يعني تحز في خاطري (عينها دمعت) كنت أتمنى.. وسكتت''*  ***Quote_IDI_9:***  *المقابلة: عام , أتحسين مرضه أثر على علاقتش مع محيطش يعني عائلتش , على زوجش , أولادش, أصدقائش , إلتقاءاتش وياهم يعني كل شي بالتفصيل*  *الأم: أي يعني من بعض الأحيان, مثلا مثلا المناسبات الإجتماعية*  ***Quote_IDI_1:***  *"من الناحيه السلبيه انقطعت اجتماعيا يعني اذا في حفله زواج او شي ما اقدر اروح أكون تعبانه و مرهقه و ما الي مزاج كلج "*  ***Quote_IDI_10:***  *"* ***اي قبل كنت اني على طول اطلع وي يعني اهل زوجي اروح اقعد اياهم وي ربعي الحين محد يشوفني زين يعني اختفيت تقريباً اختفيت عنهم"***  ***Quote_IDI_12:***  ***أهلي بس مرة في الأسبوع, أصدقائي بس في الدوام , طلعات ما عمري أطلع من يوم جبت (اسم المريض) الحين***  ***Quote_IDI_2:***  ***أتمنى اطلع واروح ويا البنات عندي واجد امنيات امنياتي كلها ما صارت*** | **The effect of caregiving on time spent with family and**  **friends** |  |
| **Code: Mother afraid of having a child with SCD**  **Number: 18 ,19 ,4,7** | ***Quote_IDI_18:***  *عقب 5 أشهر حملت بهذي(اسم البنت).. وكنت خايفة واجد أنه تكون*  *سكلر.."*  ***Quote_IDI_4:***  *في عندكم خوف من الانجاب؟ المقابلة: الام: اي لان لازم طفل انابيب ومرتين رحنا ماصار نصيب"*  ***Quote_IDI_7:***  *"يقولي أتمنى يكون غندي اخ بس كنت أقوله اذا جبت اخ ما بقدر اعطيك هالاهتمام و هذا فعلا اللي بيصير و خاطري بعض الأحيان بس خايفه يطلع مرسض و هالشي مسؤوليه ثانيه و انا وصلت عمر مغين امتى بكون معاه انا قدرت اعطي اهتمام"*  ***Quote_IDI_19:***  *حاليا طاقتي ما تستوعب اجيب شي يزيد ضغطي اخاف انه اجيب بنت واجيبها نفس (اسم المريض) خلاص احس اني انفجر ما اقدر اواصل إذا اجيب بنت او شي ما اقدر* | **Feeling Afraid** | **The impact of caregiving on emotional and physical wellbeing** |
| **Code: caregiver scared to loss child**  **Number: 1, 2 , 4** | ***Quote_IDI_4:***  *4"انا وقت اللي تمرض كله اقول بتروح عني من كثر ما اشمع اموات سكلر على الرغم نوباتها كلش بسيطة بس من احستجيها نوبة عندي خوف افقدها هي ضناي ويقولج هذا المرض مايعرف صغير ولا كبير"*  ***Quote_IDI_1:***  *"أخاف افقدهم خصوصا (اسم المريض)"*  ***Quote_IDI_2:***  *يعني اذا تبي ابرة انتظري الى الساعة اللي عطوج إياها احنا امس من 11 الى 4 اليوم توهوم يعطونها الابرة اهي تعبت تعبت اني ما هقيتها تعيش هقيتها بتموت يعني خلاص"* |  |  |
| **Code: No regrets marrying carrier husband**  **Number: 11, 10** | ***Quote_IDI_10:***  *”****ما حسيت بالندم لان مكان في وقتنا احنا في تحاليل و مثل ما قلت لش بعد ماخدين عليي تحاليل فأني لو عندي ان الزمن يرجع ورى اكيد ما باخد ان واحد حامل سكلر"***  ***Quote_IDI_11:***  ***"لا ما عندي شعور لو اني يعني اخدته غصباً عن هدي و اني ادري ان اني مريضة و اخدته اي بحس بالذنب بس اني ما احس بالذنب لان اني يعني ويش اقول ليش يعني شي مو بإرادتي اني صار شي يعني مو بإيرادتي"*** | **Regret feeling** |  |
| **Code: feeling regrets marrying carrier husband**  **Number: 8 (twice), 19, 18** | ***Quote_IDI_8:***  *"****اي ندمانة على زواجي من واحد حامل، يعني اني اقول لش موتتي الأولاد عرفتي يعني موتتي اليهال فإن لو رجع بي الزمن مستحيل"***  ***Quote_IDI_8:***  ***"يعني ألحين لو أقولش ان مجنون (ترفع نبرة الصوت) الي ياخذ وش اسمها يعني اثنين سليم و مصاب اوكي اما حامل و حامل لا مصاب و مصاب لا لأن بتكون حياتهم جحيم"***  ***Quote_IDI_19:***  ***"لا للحين المفروض اني ما اخذ زوجي من البداية يعني ماخذه لازم اقتنع انه لو اصريت على رايي اني ما اخذ زوجي ما كان صار كل هذا لو ماخذه"***  ***Quote_IDI_18:***  *" اصيح على طول اصيح والوم روحي وأقول لنفسي اني السبب لو ادري لو ادري جان سويت فحص قبل الزواج"* |  |  |
| **Code: caregiver always cry due feeling regrets**  **Number: 1, 4** | ***Quote_IDI_1:***  *تشوفيني كل يوم بالليل اصيح وأي وقت لاني احس بألم و ندم واجد الوم نفسي بطريقه ما تتخيلينها"*  ***Quote_IDI_4:***  *اقولها مو اني السبب الله كاتب.. اني ماكنت ادري بس*  *حتى ساعات اقعد اصيح..* |  |  |
| **Code: feeling regret being the reason of child disease**  **Number:1, 13, 16** | ***Quote_IDI_13:***  *اذا شفتها تتألم اقول يارتني ما جبتها"*  ***Quote_IDI_1:***  *"تشوفيني كل يوم بالليل اصيح وأي وقت لاني احس بألم و ندم واجد الوم نفسي بطريقه ما تتخيلينها"*  ***Quote_IDI_16:***  *: لا, يعني أكي ساعات أصير مواقف أقول ليه, أحس بالندم و جدي جدي , بس يعني عموما ما أتكلم ويا أحد* |  |  |
| **Code: Caregiver shocked after the child diagnosed with SCD**  **Number: 11, 14,10,9, 19** | ***Quote_IDI_14:***  *"اول ما صادها ما عرفت انصدمت "*  ***Quote_IDI_11:***  *"****مادري يعني انصعقت اول في البداية حتى ما نمت ما نمت يعني حسيت ان شي خبر يعني مفاجئ بالنسبة اليي تفاجأت (سكوت) يعني كأن مو حلم شفتين كأن واحد قايل يش حلم و صار في شي يعني تبين تنتبهين منه؟ جدي كأنه حلم و ابي انتبه منه"***  ***Quote_IDI_10:***  ***"يعني اكيد انصدمت يعني انهرت (انخفاض حدة الصوت) يعني صدمة بالنسبة ليي ان احنا نسمع عن هالمرض بس مااكان موجود عندنا"***  ***Quote_IDI_19:***  ***"يعني صدمه اول ما تعرفين انه ولدش انه مصاب سكلر"***  ***Quote_IDI_9:***  ***فقالو ليي فانصدمت. بداية الصدمات خوب يعني بس الحمد الله*** | **feeling after diagnosis** |  |
| **Code: After SCD diagnosis, father is more affected**  **Number:8, 14** | *"* ***Quote_IDI_14:***  *ما يتقبل يعني لدرجة لين يسألونه يقول حامل يعني مو مصابة بعدين رضا بالامر صار بس تميت اني اتعامل وياهم "*  ***Quote_IDI_8:***  *"****في البداية يعني اني اول مما يبتها ريم ابوها هو الوحيد الي تأثر تأثرت حياته ويايي"*** |  |  |
| **Code: Caregiver felt sorry for child.**  **Number: 9** | ***Quote_IDI_9:***  *:مسكين (اسم المريض)* | **Feeling Sorry** |  |
| **Code: Caregiver apologize to child for having SCD**  **Number: 8, 14** | *"* ***Quote_IDI_14:***  *حتى لين قلت ليه ماما سامحني ما قصدت اني اسوي فيك جدي بس دائما اطلب من الله انه الله يرأف بحال ولدي ويخليه ليي"*  ***Quote_IDI_8:***  *"****في البداية قلت ليها مثلاً سماحيني اني اني جبتش على هالدنيا و انتين الوحيدة في البيت الي مريضة يعني ما اخدتين حياتش الطبيعية مثلاً في الطفولة مثلاً بعد ما كبرتين ما ماراست حياتها انها لعبت نفس باقي الأطفال الي في سنها"*** |  |  |
| **Code: Caregiver faced psychological stress from being in hospital**  **Number: 18,2, 16 ,7 ,19,4** | ***Quote_IDI_18:***  *"بس أكون تعبانة ابغي انام بس اقوي نفسي"*  ***Quote_IDI_16:***  *مو تصيرين عصبية و إنما أتصيرين under stress, فاهمة؟ أنش أتصيحين مضايقة جدي يعني*  ***Quote_IDI_2:***  *اني هني ما انام يجيني خوف مرات اشوفها ما تتنفس واخاف عليها اروح ادور في الممرات شوي اهدي نفسي مافي الا انا وياها اذا صادها شي بيقولون مني*  ***Quote_IDI_19:***  *" (اسم المريض)خلاص اني ما اقدر اللحين صار ليي اليوم 12 حتى نفسيتي الممرضات يقولون لي لو رايحه مسافرة رجعتين ما اقدر خلاص"*  ***Quote_IDI_7:***  *"انا كل هالفتره نفسيتي اقل من الصفر"*  ***Quote_IDI_4:***  *الام نفسيا تعبت تعبت "* | **Psychological stress** |  |
| **Code: Psychological stress affected caregiver’s health**  **Number:12, 2, 8, 4** | ***Quote_IDI_8:***  *"****حتى اني اعاني بعد من بعض المشاكل أمراض يعني عندي القولون و المعدة يعني زادت بزيادة توتري وياها، ما أحسسها بهذا الشي بس تعرفين الأم يعني ، القولون يزيد في حالة التوتر و العصبية الي عندي و الأكل طبعا نوعية الأكل، نوعية الأكل اني خب من أرتبط بها في المستشفى ما أقدر آكل من لبيت على طول فأضطر إن أني آكل ن برا و أكل المطاعم بالنسبة إلي مأساة يعني يسبب ليي إسهال تعرفين يعني القولون و هالسوالف"***  ***Quote_IDI_4:***  *المقابلة: تحسين ياثر ع صحتج؟؟****"***  *الام: اي اكيد اني ما احصل راحة احس بالم فلراس والظهر وبعد مرضها امرض****"***  ***Quote_IDI_12:***  *يعني واجد واجد ينشغل بالي , لدرجة أن قاعد يصيدني ضغط , سكر من كثر ما أن أني أفكر في الموضوع, أرق يصيدني*  ***Quote_IDI_2:***  *بنت العم: أي ساعات أقول ابي انتتحر*  *المقابلة:شنو!! شلون!!*  *بنت العم: ههه ساعات أقول ابي انتحرّ*  *المقابلة: يعني من التوتر ولا انتي حزينة عليها*  *بنت العم: ساعات لما ما اشوفها تتنفس انا بروحي تبوني استجنّ قبل كنت اطلع اخفف على روحي"* |  |  |
| **Code: Caregivers feels their condition is better than others**  **Number: 12, 15, 18,17,11, 16,4** | ***Quote_IDI_18:***  *ولين اشوف الحالات الثانية في قسم الأطفال اتصبرأقول احنه اهون"*  ***Quote_IDI_17:***  *"* ***دشينا اول أسبوع جناح الأطفال.. تشوفين الي وياه اله لوكيمبا واله مو عارفين وش فيه واله اله فيعني سيده جيت قلت لزوجي نحمد ربنا الله عطانا ولد سكلرنعرف مرضه ونعرف علاجه ونعرف نتجنب النوبات ونقدر نحافظ عليهم ولا الامراض الي شفناهاالصعبة الي ماليها علاجات..***  ***قمن جذيها خذينا شوية رضا انه احنه عنده سكلر مو الامراض الي شفناها***  ***ما بقولش انه ما يأثر امبله ياثر***  ***لكن الحمدلله انه سكلر مو شي غير"***  ***Quote_IDI_16:***  ***:المستشفى يجوف الناس اللي حالاتهم المرضية أخس, أنت جوف الله يحبك الله أبتلاك في ناس جوف ما يقدرون يمشون ما يقدرون يسمعون و ناس اصلا ما عندهم أم ما عندهم أبو , أنت الله عطاك شي و أخد شي فلازم تحمد ربك لازم أه ... تحمد ربك و تشكره على نعمة اللي عندك***  ***Quote_IDI_11:***  ***"كل اقول هي احسن من غيرها دايماً اقول هي احسن من غيرها يعني اني اشوف يعني مصابين السكلر اني عندي ود حميي سكلر كان واجد يترقد فهي صايرة يعني دايماً اقول هي احسن من غيرها"***  ***Quote_IDI_15:***  ***:أحنا ما عندنا معاناة أكثر من غيرنا, معاناتنا بسيطة الحمدالله الحمدالله***  ***أي فيه ناس نتحجى وياهم و بعد يعني ويا الأهل و في ناس يعني ما يحبطونش أو يسدون الباب في وجهش بالعكس يعني تسمعين بلاويهم أتهون مصيبتش يعني الحمدالله***  ***Quote_IDI_4:***  ***لما اروح المستشفى واسوف الاخرين اقول الحمدلله.. لما اوديها واشوف غيرها تقولين الحمدلله حتى هي نفسها تشوف المصابين هناك يصرخون من الالم تقول حمدلله اني مو جذي مو نفسهم ف هذي مشيئة الله"***  ***Quote_IDI_12:***  ***عقب ما جفت أنهو ما يمرض واجد قلت الحمدالله أن أني .. يمكن هو أفضل من غيره , بعد يوم قام الحين يمرض بعد قلت أفضل حتى من أبوه , أفضل من أشخاص ثانيين*** | **Feeling Satisfied as coping mechanism** |  |
| **Code: Caregiver do not talk openly about her issues to people.**  **Number: 9, 16, 7** | ***Quote_IDI_7:***  *بس أحاول ان هالتاثير ما انقله لابني او زوجي لان زوجي واجد يتاثر فلازم أكون انا صامده حتى في المستشفى اهو ينهار اكثرمني"*  ***Quote_IDI_9:***  *:إذا مثلا واجهتش يعني صعوبات أو ضيق عاطفي من هالناحية تتحدثي مع من؟*  *الأم: والله أصيح بروحي*  ***Quote_IDI_16:***  *الأم: بس أصيح بروحي*  *المقابلة: ما تتكلمين مع أحد يعني؟*  *الأم: جك (هزة رأسها)* | **Express feelings** |  |
| **Code: Caregiver do not show her struggle to child**  **Number: 9** | ***Quote_IDI_9:***  *لا بالعكس صوبه كلش غير أني, أوكي يعني ما أبين ليه* |  |  |
| **Code: Fatigue due to lack of sleep**  **Number: 18, 1, 10, 5, 13, 14, 4, 19, 7** | ***Quote_IDI_18:***  *بس أكون تعبانة ابغي انام بس اقوي نفسي"*  ***Quote_IDI_13:***  *13 " تقريباً 5 إلى 6 ساعات ما أنام شي على طول الممرضات يكونون موجودين يعني كل بين ساعه يجون فواجد تعب"*  ***Quote_IDI_4:***  *4"اذا هي مريضة ما تغطي عيوني على طول اروح واجي او ساعات اقعد وياها ف نفس الغرفة"*  ***Quote_IDI_19:***  *19"ظروفي بس يعني 5 ساعات بالكثير"*  *1" ما انام تقريبا'*  ***Quote_IDI_14:***  *14 " بعد ما اواصل انام تقعدني بتروح الحمام يعني لما احصل وقت تغط عيوني نص ساعه ربع ساعة وارد أقوم"*  ***Quote_IDI_5:***  *"المقابلة: شلون اثر النوم عل دراسة والشغل؟*  *التعب مضاعف وبالذات ما كان عندي احد زوجي ما يقدر ينام ف العام لان كل حريم ف انا ما كنت انام اروح مواصلة اقدم امتحان وبسرعة ارجع له ف الدكتور كان يتعاون وبسرعة يرجعني البيت*  ***Quote_IDI_10:***  *“الأم:* ***لا من البداية كنت ما انام كل تفكير فيه و للحين يعني ادا يمرض ما انام.***  ***المقابلة: يعني تحسين جاش ارهاق مثلاً؟***  ***الأم: اكيد اتعب"***  ***Quote_IDI_1:***  *متعب من كل النواحي جسديا لان ما نام خاصا*  ***Quote_IDI_7:***  *وايد أيام اواصل فيها بعض الأحيان 3 ساعات او اقل* | **Fatigue** |  |
| **Code: caregiver is fatigue during child admissions**  **Number: 1, 9, 16** | ***Quote_IDI_1:***  *يعني قبل كان عندي حيويه بس اللحين دايما تعبانة"*  ***Quote_IDI_9:***  *أنزين حسيت روحي ثلاثة أيام أجي ليه صح و عصر, ثلاثة أيام خاصة ثالث يوم كنت شوي منهار جسمي تعبان*  ***Quote_IDI_16:***  *حسيت تكسير في الجسم, تعب* |  |  |
| **Code: no financial problems**  **Number: 15, 13, 9, 16,4** | ***Quote_IDI_13:***  *الدعم اوكيه ما عندي إلا هي بس فمو مهتمة بالفلوس"*  ***Quote_IDI_15:***  *الحمدالله رب العالمين الحمدالله يعني حالتنا لا هي ضعيفة يعني قولي ما بين المتوسطة و الجيدة الحمدالله الحمدالله رب العالمين أحنا ما أنروح نتسلف و ندين يعني من هدا و هدا, أبو (اسم الاخ) ما يأخذ من عند أحد ما ينام, ما ياخد من أحد فلس و ينام يبي ينام و هو مرتاح الحمدالله كل شي موفر لينا كل شي*  ***Quote_IDI_4:***  *"الام حمدلله ميسورة"*  ***Quote_IDI_9:***  *المقابلة: أوكي .. طبعا ما كان عندكم أزمات مالية صح و أثر عليه؟*  *الأم: الحمدالله*  ***Quote_IDI_16:***  *كلش ما أعاني من أزمات مالية* | **financial status** | **The impact of caregiving on financial life** |
| **Code: poor financial state of caregiver**  **Number: 8, 1, 10 ,19** | ***Quote_IDI_10:***  *"*  ***صراحة ما نصفي على شي (سكوت) ما نصفي على شي بس الراتب يروح"***  ***Quote_IDI_8:***  ***اني حالتي المادية اعتبرها ضعيفة انزين شفتين شلون نفقتي 150 و اني معاشي 160"***  ***Quote_IDI_19:***  ***"على راتب زوجي على 150 ما يكفي"***  ***Quote_IDI_1:***  ***"يعني عايشين ب 150 دينار"*** |  |  |
| **Code: Work is tolerant with caregiver**  **Number:12, 1, 16** | ***Quote_IDI_1:***  *“الصراحة شغله وايد متفهمين كل ما يصيده نوبه و يضطر يطلع وايد متفهمين ويعطونه فرصه"*  ***Quote_IDI_12:***  *المقابلة: صار عندش مثل هذا الظرف أشلون أتوفقين بين العمل و بين زيارة الطفل؟*  *الأم: يعني يعرفون أن أني مرقدة.*  *المقابلة: يعني يعطوش سك ليف؟*  *الأم: يعطوني إجازة مرضية .*  ***Quote_IDI_16:***  *قصدي أنهم شوي tough في موضوع الغياب, بس للأمانة كانو flexible with me, يعني أن لحد الأن إني ما داومت بس من خلال المحادثات وياي في الوتساب لأ كانو flexible* | **Work status** |  |
| **Code: Disease has no effect on work life for caregivers**  **Number: 15** | ***Quote_IDI_15:***  *المفابلة: أوكي هل أثر حالة الطفل على بعد العمل؟ هل مثلا غيب مثلا يعني غاب الأبو مثلا عن عمله أو غبتين من العمل بسبته؟*  *الأم: لا لا لا , ما غبت, ما غبت عن العمل* |  |  |
| **Code: caregiver work is affected because of the disease**  **Number: 4, 19, 5** | ***Quote_IDI_19:***  *: اي ما عندي احد فاضطريت اني اقعد في البيت "*  ***Quote_IDI_4:***  *"اجي المدرسة ماني محضرة ولاشي يعني حتى البنات اهملهم بلذات مادة الاجتماعيات يبالها تحضير"*  ***Quote_IDI_4:***  *"اي فترة اللي ارقد وياها اغيب 3 و 4 ايام تعرفين حدش اجازة 3 ايام لو 4 يبيلش تقرير وهلشي صادني وياها تطوري الوظيفي تاثر بس البنت بدات تتحمل مسوولية نفسها"*  ***Quote_IDI_5:***  ***شلون اثر النوم عل دراسة والشغل؟***  ***التعب مضاعف وبالذات ما كان عندي احد زوجي ما يقدر ينام ف العام لان كل حريم ف انا ما كنت انام اروح مواصلة اقدم امتحان وبسرعة ارجع له ف الدكتور كان يتعاون وبسرعة يرجعني البيت"*** |  |  |
| **Code: leaving work to take care of patient**  **Number: 9, 18, 19 ,7** | ***Quote_IDI_18:***  *" كنت أشتغل قبل بس الحين ما أشتغل عشان أعتني ببتي.."*  ***Quote_IDI_7:***  *"قبل كان عندي وظيفه و حياه اجتماعيه"*  ***Quote_IDI_9:***  *ما قعدت في كذا شغلة ما قعدت و السبب أن الغياب*  ***Quote_IDI_19:***  *"قعدت من الشغل لانه كان عندي ولدي ما إليه احد"* |  |  |
| **Code: Child admission caused mother to be absent**  **Number: 11, 4, 5** | ***Quote_IDI_11:***    *"****من قبل لان ما ترضى تباني اني انام اياها يعني كانت صغيرة هي له فأضطر ان اني ما اروح المدرسة "***  ***Quote_IDI_4:***  ***"*** ***اي فترة اللي ارقد وياها اغيب 3 و 4 ايام تعرفين حدش اجازة 3 ايام لو 4 يبيلش تقرير وهلشي صادني وياها تطوري الوظيفي تاثر بس البنت بدات تتحمل مسوولية نفسها"***  ***Quote_IDI_5:***  ***"*** ***والهم ياثر ف شغلي لكن اثر اني اخذ اجازات واثر في التقرير السنوي"*** |  |  |
| **Code: Moving from full time job to part time job**  **Number: 17** | ***Quote_IDI_17:***  *"* ***أشتغل .. بس بسبب ظروف صحتي وظروف أولادي تحولت من فل تايم إلى بارت تايم"*** |  |  |
| **Code: Work was not tolerant with caregiver’s absence**  **Number:9 ,7** | ***Quote_IDI_7:***  *"واجد مشاكل و ما يتقبلون و لا يتفهون ان ابنه مريض و هذا اثر واجد على نفسيه زوجي"*  ***Quote_IDI_9:***  *:الأم: ما يتقبلون الأشغال للأسف يعني*  *المقابلة: ما يتقبلون ؟*  *الأم: أقول أنه ما يراعون يعني بتغيبين أنتين بتغيبين مثلا يوم أوكيه بس إذا مثلا أمرقد أهني و كان صغير لازم أني كنت مرافقة وياه فيلومونش يعني* |  |  |
| **Code: Buying food during admission affecting budget**  **Number: 11, 8, 1** | ***Quote_IDI_8:***  *"*  ***هذا يأثر عليي بعد ان اني اعطيها مثلاً في اليوم مو اقل من دينارين يعني وجبة يوصلونها اليها دينارين و نص تقريباً يعني حسبيها بعد خلال هاي تقريباً 13 يوم 12 يوم كم ضربي دينرين في 12 24 دينار تقريباً هذا بس حق وجبة وحدة يعني الباقي مثلاً اجيب ويايي اغراض من البيت و هذي يأثر عليي"***  ***Quote_IDI_11:***  ***"مادري يعني يمكن من سالفة الاكل يعني هي ما تاكل من عندهم المستشفى فنضطر نشتري ليها يعني نشتري ليها اكل او نجيب ليها من البيت"***  ***Quote_IDI_1:***  ***"الصراحه الاكل وايد مكلف بالنسبه لي لان ما يتقبل اكل المستشفى"*** | **Buying food during hospitalization** |  |
| **Code: Buying healthy food affects budget**  **Number:12** | ***Quote_IDI_12:***  *أنزين و الأكل الصحي أتحسين أن يأثر على ميزانيتش بعد و إلا؟*  *الأم: أي يأثر واجد* |  |  |
| **Code: Buying medicines affects budget**  **Number:12,1** | ***Quote_IDI_1:***  *"*  *الان الدوا من السعوديه و ثمنه11 دينار و صعب علي اتكفل بمصارف هالدوا و مو دايما المبلغ متوفر عندي لان وضعنا صعب"*  ***Quote_IDI_12:***  *الأم: أي أشتري واجد*  *هل يأثر على يعني حالكم الما...؟*  *الأم: واجد يأثر* | **Buying medicine** |  |
| **Code: Adolescent do not want the hospital’s ibuprofen so caregiver buy it**  **Number: 18** | ***Quote_IDI_18:***  *"*  *اشتري ها بس البروفين هم يعطوني ف السلمانية بس هي ما تبغي الا اشتريه"* |  |  |
| **Code: Caregiver have health insurance**  **Number:14** | ***Quote_IDI_14:***  *" زوجي عنده بطاقة انشورنس (تأمين)"* | **Insurance** |  |
| **Code: Caregiver has health insurance (private hospital)**  **Number: 16** | ***Quote_IDI_16:***  *: أنتين قلتين أن أتروحين أبن النفيس هاي ما أثر على الميزانية يعني؟*  *الأم: لا لأن هو already عنده تأمين صحي.* |  |  |
| **Code: financial aid from charities**  **Number: 8** | ***Quote_IDI_8:***  *"****عندي مساعدة مالية من الجمعية الخيرية مال (اسم الجمعية) 50 كان الاول يعطوني 37 بس لان طرشت ليهم رسالة كتبتها شافوا حالتي قالوا لاه تستدعي ان اني اكثر"*** | **Coping with financial crisis** |  |
| **Code: refusal of charity to give financial support**  **Number:10, 3** | ***Quote_IDI_3:***  *" والحين هل تلجأ لهم؟*  *لا بس في البداية بعدين ماحسيت في استجابة منهم و علاج نهائي لمرض السكلر ف توقفت"*  ***Quote_IDI_10:***  *"****ما يعطون يقولون ليش في ناس الزم منش ميساعدون لان اي اول ما صارت عندنا الحالة جدي رحت هناك الصندوق الخيري و لا شي و لا شي و لا شي (التشديد على النبرة) كنت بعد ما رحت اليهم وقت يعني ايام عادية كنت في شهر الله قلت اليهم ابغي بس سلة رمضانية تجيبون للجهال (سكوت) و لا شي (التشديد على النبرة) الي يبونه بس"*** |  |  |
| **Code: caregiver try to balance finance to prevent loaning**  **Number:10** | *"* ***Quote_IDI_10:***  ***الأم: من قبل كنا ناخد يعني نتسلف مو قرض اني عندي خت ساعات اتسلف من عندها لين يصير عندنا اسد ليها.***  ***المقابلة:و الحين؟***  ***الأم: الحين اخلي روحي جدي اضغط روحي تعبنا يعني من السلف"*** |  |  |
| **Code: Caregiver receives financial aids from relatives**  **Number:2, 8, 12, 18** | ***Quote_IDI_ 18:***  *" نضغط على روحنه ونتسلف من وحدة من خواته لكن هو ما يحب يطلب كريه الشي خصوصا ع الرجال.."*  ***Quote_IDI_2:***  *"اكيد ابوي وابوها يتعاونون احنا مو بس هادي المريضة"*  ***Quote_IDI_12:***  *: أذا مثلا أزمات مالية و إو شي شلون تتعاملين معاهم؟*  *يعني ويا بيت أبويي يتحملون وياي*  *يعني مثلا تتسلفين منهم؟*  *أم (هززت رأسها)*  ***Quote_IDI_8:***  *"****عندي وحدة الله راسلنها اليي تقريباً هاي السنة الثانية تطرش لي مبلغ تقريباً 60 دينار يعني كمساعدة مساعدة مديرة هي من صوبنا يعني معرفة فتعرف حالتي فقامت تطرش ليي,الحمدلله خواتي فيهم المدرسات فيهم المتقاعدين يساعدوني"*** |  |  |
| **Code: Loaning to cover private room expenses**  **Number: 18** | ***Quote_IDI_18:***  *نضغط على روحنه ونتسلف من وحدة من خواته لكن هو ما يحب يطلب كريه الشي خصوصا ع الرجال.."* |  |  |
