# Supplementary material for: Caregivers’ experience of seeking care for adolescents with sickle cell disease in a tertiary care hospital in Bahrain
Source: PLoS One. 2022 Apr 7;17(4):e0266501. doi: 10.1371/journal.pone.0266501 (PMC8989311; doi:10.1371/journal.pone.0266501)
Supplement: S1 Appendix — (DOCX) [file pone.0266501.s004.docx]

## **Appendix 1**

**Quantitative Questionnaire**

|  |  |  |
| --- | --- | --- |

**I.D Number**

**The challenges and the impact of being a caregiver of Sickle cell disease adolescent patients in Bahrain**

**Instructions:**

1. **Please answer all the questions.**
2. **Write the answers directly on the questionnaire.**
3. **The questionnaire contains different type of questions viz.:**

**(a)  Some questions require specific information about the caregiver or the patient e.g. Age, Date of birth etc.**

**(b)  Some questions are of Yes/No category, where only one option can be selected.**

**(c)  Some questions allow the selection of more than one option.**

| **Demographic Data of the caregiver** | | | | |
| --- | --- | --- | --- | --- |
|  | **Questions** | **Options** | **Response** | **Remarks** |
| 1. | How old are you? | years |  |  |
| 2. | What is your date of Birth? | / / |  |  |
| 3. | Gender | 1. Male 2. Female |  |  |
| 4. | What is your current marital status? | 1. Married 2. Single 3. Divorced 4. Widowed |  |  |
| 5. | If Divorced, is the current marital status because of the patient? | ----------------------------- |  |  |
| 6. | What is your relationship to the patient? | 1. Mother 2. Father 3. Sister 4. Other? ----------------- |  |  |
| 7. | What is your current occupation? | 1. Student 2. Looking for job 3. Free work 4. Public sector employee 5. Private sector employee 6. Retired 7. Unemployed |  |  |
| 8. | What is your current educational level? | 1. Illiterate 2. Primary school 3. Intermediate school 4. High school 5. Diploma 6. B.S.C 7. Master 8. PhD |  |  |

| **Demographic Data of the patient** | | | | |
| --- | --- | --- | --- | --- |
|  | **Questions** | **Options** | **Response** | **Remarks** |
| 1. | Gender | 1. Male 2. Female |  |  |
| 2. | How old is the patient? | years |  |  |
| 3. | What is the patient’s date of birth? | / / |  |  |

| **Health Assessment: To understand the healthcare accessing problems faced by caregivers.** | | | | |
| --- | --- | --- | --- | --- |
|  | **Questions** | **Options** | **Response** | **Remarks** |
| 1. | Where is your first destination when the patient has a sickle cell crisis? | 1. Healthcare center 2. SMC 3. Private clinic 4. Traditional Medicine |  |  |
| 2. | Why did you choose it as a first destination? | 1. Distance 2. Quality of services 3. Availably of doctors 4. Other? ------------------ |  | Multiple Options Allowed |
| 3. | What are the challenges you face on your way to the hospital? | 1. Lack of transportation 2. Road traffic 3. Insufficient Parking 4. Other? ----------------- |  | Multiple Options Allowed |
| 4. | How long does it take you to reach there? |  |  |  |
| 5. | Do you have one regular doctor for your child? | 1. Yes 2. No |  |  |
| 6. | If yes, did you choose that one doctor? | 1. Yes 2. No |  |  |
| 7. | If you chose one regular doctor, what is your reason? | 1. Professionalism 2. Knowledge 3. Confidence 4. Humility 5. Other? ------------------ |  | Multiple Options Allowed |
| 8. | How many times the patient has visited the emergency room in the last six months? | No. of visits |  |  |
| 9. | How many times the patient has been admitted to the hospital in the last year? | No. of admission |  |  |
| 10. | How many times the patient has visited the doctor for follow up in the last year? | No. of visits |  |  |

| **General Assessment: To assess the influence of being a caregiver on social, emotional and financial life.** | | | | |
| --- | --- | --- | --- | --- |
|  | **Questions** | **Options** | **Response** | **Remarks** |
| 1. | On a scale of 1 to 5, how do you personally evaluate the support you get from your family? | 1. 1 2. 2 3. 3 4. 4 5. 5 |  |  |
| 2. | Specify the type of the support that you receive: | 1. Emotional support 2. Financial support 3. Informational support 4. Others? ---------------------- |  | Multiple Options Allowed |
| 3. | How does the amount of time spent in hospital with the patient affect the time for yourself? | 1. It doesn’t affect at all 2. It affects sometimes 3. I barely have time for myself |  |  |
| 4. | How does the condition of your child affect your relationship with each of the following: | Partners |  |  |
|  |  | Family member |  |  |
|  |  | Other children |  |  |
|  |  | Friends |  |  |
|  |  | Co-workers |  |  |
| 5. | On an average how much do you spend on the patient’s treatment monthly? | BHD |  |  |
| 6. | What’s the monthly average household income from all sources? | BHD |  |  |
| 7. | How many people in the household work? |  |  |  |
| 8. | What’s the main source of bearing the cost of the treatment? | 1. Salary/money from job 2. Financial support from family 3. Remittance from people 4. Business/financial investments 5. Others? ---------------------- |  | Multiple Options Allowed |
| 9. | What kind of health insurance do you have? | 1. Private health insurance 2. Public or government health insurance 3. Emergency insurance 4. I don’t have an insurance 5. Others? ---------------------- |  |  |
| 10. | Do you have a health insurance for your child’s condition? | 1. Yes 2. No |  |  |
| 11. | What is the impact of the patient’s chronic health condition on the family’s finances? | 1. It affects the kitchen expenses 2. It affects the educational expenses 3. It affects the rent 4. It doesn’t affect at all 5. Others? ---------------------- |  | Multiple Options Allowed |
| 12. | What is your own strategy to cope with the health expenditure in time of financial crisis? | 1. Sell household items 2. Look for a side job 3. Take a loan 4. I don’t have strategy 5. Others? ---------------------- |  | Multiple Options Allowed |
