# Supplementary material for: Caregivers’ experience of seeking care for adolescents with sickle cell disease in a tertiary care hospital in Bahrain
Source: PLoS One. 2022 Apr 7;17(4):e0266501. doi: 10.1371/journal.pone.0266501 (PMC8989311; doi:10.1371/journal.pone.0266501)
Supplement: S2 Appendix — (DOCX) [file pone.0266501.s005.docx]

## **Appendix 2**

Start time ______: ______am\pm

Finish time _____: ______am\pm

Participant ID:

Date: ______\________\_________

**Qualitative interview guide**

**Section A: introduction and Opening statement:**

My name is ------------ I am a third year medical student at Arabian Gulf University. We request you to participate in this study about caregiver’s different aspects of life and experience with a sickle cell disease adolescent and how dealing with sickle cell disease adolescent affects your life, emotions, social and family income and to know more about your journey challenges while taking care of adolescent of sickle cell disease.

Interviewer note: please read out the information sheet to the participant and record their answers. next, read out the consent form and ask the participant to sign if they are agreed to proceed after explaining the harms, participant’s rights and consent aspects. Warning this questionnaire will recall some unpleasant events and strong emotional involvement. Please use the guide below and answer any question by caregivers regarding the research.

Thank you for agreeing to be a part of this study. Please feel free to open to us as your identity will be hidden and all personal data are highly confidential. we expect you to answer the questions with full honesty, yes or no answers are not accepted. In the beginning,

Can you tell me about yourself?

**Probes:**

- Can you tell me your name and age?
- What is your educational level?
- What do you do for living?
- What is your marital state?
- What is your relation to the child?

**Can you tell me about your child’s with sickle cell disease?**

**Probes:**

- Can you tell me your child’s name and age?

Interviewer note: if the caregiver has more than one sickled disease child, ask her to talk about only one.

**Section B: Healthcare Accessing Challenges**

Now, we would like to know about your **experience using health care.** health care accessing starts from getting an appointment or during having crisis in emergency department until receiving full health care. **Can you tell me about your experience using health care?**

**Probes:**

1. Where do you go to receive medical care?
2. How do you get there?

**What are the struggles and difficulties you face while trying to get health care access?**

**Probes:**

1. Time needed to reach the hospital.

**Section C: Emotional aspect**

Interviewer note: pay attention to the terms used by the caregiver while describing their feelings. Pay attention to body language and let the caregiver talk freely about their feelings without judgment.

**Please reflect how you are feeling about caring of adolescent with sickle cell disease?**

**Probes:**

1. How do you cope with this feeling?
2. Who do you talk to about feeling like this?
3. Do you have access to any support services e.g. websites/counseling? If so, what do you use and why?

Many caregivers experience Burnout feelings. Burnout is a state of emotional, physical, and mental exhaustion caused by excessive and prolonged stress**. Tell me more about your experience with such things.**

**Probes:**

1. How often you worry at night, have trouble falling asleep or staying asleep?
2. How often you feel less competent\ effective than before or work harder yet accomplish less?
3. Have you ever felt overwhelmed?
4. How often you see friends and family?
5. Have often you feel angry, irritated, annoyed or disappointed in people around you?
6. Have you ever suffered from physical complaints?

**Section D: Social aspect**

Interviewer note: focus on the changes that have had occur after having a child with sickle cell disease.

**Can you tell me how caring for your child’s condition affect your social life? Social life is about your relationships with your surroundings, daily activities and family gatherings.**

**Probes:**

1. How your patient’s condition affects your relationships with your family, partner and friendships?
2. Can you tell me how do you feel about your current relationships with your family members and friends?

**Section E: Financial aspect**

**We understand that there are a lot of expenses when you have a child with sickle cell disease. Can you tell me how does your child’s disease affect your financial status?**

**Probes:**

1. What are the financial problems that you face?
2. How does your child condition affect your job?
3. How do you manage taking care of your children in addition to other expenses?

**Closure**

**I am thankful to you for your participation in our study and if you have any questions please feel free to ask.**

Interviewer note: ask the participant if they would like to add anything.
