# Supplementary material for: Caregivers’ experience of seeking care for adolescents with sickle cell disease in a tertiary care hospital in Bahrain
Source: PLoS One. 2022 Apr 7;17(4):e0266501. doi: 10.1371/journal.pone.0266501 (PMC8989311; doi:10.1371/journal.pone.0266501)
Supplement: S3 Appendix — (DOCX) [file pone.0266501.s006.docx]

**استبيان**

|  |  |  |
| --- | --- | --- |

**الرقم الشخصي:**

**الاسم:**

**المعوقات التي تواجه مقدمي الرعاية لمرضى فقر الدم المنجلي من فئة المراهقين في البحرين**

**التعليمات:**

1. **الرجاء الاجابة على جميع الاسئلة**
2. **الرجاء كتابة الإجابة على ورقة الإستبيان**
3. **الاستبيان يحتوي على أنواع مختلفة من الإسئلة:**
   1. **بعض الأسئلة تتطلب معلومات معينة عن الراعي أو المريض (مثال: العمر , تاريخ الولادة .. الخ)**
   2. **بعض الأسئلة عبارة عن اختيار نعم/لا كإجابة , فقط إجابة واحدة مسموحة**
   3. **بعض الأسئلة ممكن إجابتها بأكثر من خيار**

| **البيانات الخاصة لمقدمي الرعاية** | | | | |
| --- | --- | --- | --- | --- |
| **الملاحظات** | **الاجابة** | **الخيارات** | **السؤال** |  |
|  |  | سنوات | **العمر:** | 1 |
|  |  |  | **تاريخ الميلاد:** | 2 |
|  |  | 1. ذكر  2. أنثى | **الجنس:** | 3 |
|  |  | 1. متزوج/ة  2. أعزب/ آنسة  3. مطلق/ة  4. مترمل\أرملة | **الحالة الاجتماعية:** | 4 |
|  |  | ------------------------------ | **في حال عدم الزواج \ الطلاق , هل الحالة الاجتماعية لها علاقة بالمريض :** | 5 |
|  |  | 1.الام  2.الاب  3.الاخت  4. أخرى.... | **صلة القرابة مع المريض** | 6 |
|  |  | 1. طالب  2. باحث عن العمل  3.اعمال حرة  4.موظف حكومي  5.موظف في القطاع الخاص  6.متقاعد  7.عاطل عن العمل | **الحالة الوظيفية:** | 7 |
|  |  | 1.أمي  2.ابتدائي  3.إعدادي  4.ثانوي  5.دبلوم  6.بكالوريوس  7.ماجستير  8.دكتوراه | **المستوى التعليمي** | 8 |

| **المعلومات الديموغرافية الخاصة بالمريض:** | | | | |
| --- | --- | --- | --- | --- |
| **الملاحظات** | **الاجابة** | **الخيارات** | **السؤال** |  |
|  |  | 1. ذكر  2. أنثى | **الجنس:** | 1 |
|  |  | سنوات | **العمر:** | 2 |
|  |  |  | **تاريخ الميلاد:** | 3 |

| 1. **التقييم الصحي: لفهم مشاكل الحصول على الرعاية الصحية التي يواجهونها مقدمو الرعاية لمرضى فقر الدم المنجلي:** | | | | | |
| --- | --- | --- | --- | --- | --- |
| **الملاحظات** | **الاجابة** | **الخيارات** | **السؤال** | |  |
| . |  | 1. المركز الصحي  2.مجمع السلمانية الطبي  3.عيادة خاصة  4.أدوية تقليدية | **ما هي وجهتك الاولى عندما يعاني مريضك من نوبة السكلر:** | | 1 |
|  |  | 1.المسافة  2.جودة الخدمات  3.وفرة الاطباء  4.أسباب أخرى؟...... | **ما هو السبب الرئيسي لجعل هذا المكان وجهتك الرئيسية (وجهتك الاولى)؟** | | 2 |
|  |  | 1. عدم توافر وسائل المواصلات  2. الازدحام المروري  3. عدم وجود مواقف كافية للسيارات | **ما هي المشاكل التي تواجهك للوصول الى المستشفى؟** | | 3 |
|  |  | ------------------------------------ | **كم تستغرق من الوقت للوصول الى المستشفى؟** | | 4 |
|  |  | نعم / لا | **هل يوجد طبيب معين يتابع الحالة الصحية بشكل منتظم؟** | | 5 |
|  |  | نعم / لا | **إذا كانت الإجابة نعم، هل أخترت هذا الطبيب؟** | | 6 |
|  |  | 1. ذو خبرة إحترافية 2. ذو علم و معرفة 3. موثوق به 4. متواضع 5. أسباب أخرى؟.............. | **ما هو سبب إختيارك لهذا الطبيب؟** | | 7 |
|  |  |  | **خلال الاشهر الستة الماضية، كم عدد المرات التي تم فيها ادخال المريض الى الطوارئ؟** | | 8 |
|  |  | ------------------------------------ | **خلال السنة الماضية، كم عدد المرات التي تم فيها ادخال المريض الى المستشفى؟** | | 9 |
|  |  | ------------------------------------ | **خلال السنة الماضية، كم عدد المرات التي تم فيها متابعة حالة المريض من قبل الدكتور؟** | 10 | |

| **التقييم العام: لتقييم تأثير كونهم مقدمي رعاية لمرضى فقر الرم المنجلي على حياتهم الاجتماعية، العاطفية و المالية** | | | | |
| --- | --- | --- | --- | --- |
| **ملاحظات** | **الاجابة** | **الخيارات** | **السؤال** |  |
|  |  | 1 .1  2 .2  3 .3  4 .4  5 .5 | **على مقياس من 5، كيف تقيم شخصياً الدعم العائلي الذي تحصل عليه من عائلتك؟** | 1 |
| يسمح بأكثر من خيار |  | 1. دعم عاطفي  2. دعم مادي  3. دعم معلوماتي  4. أخرى؟ ----------- | **حدد نوع الدعم الذي تحصل عليه:** | 2 |
|  |  | 1. لا يؤثر على الإطلاق  2. يوثر أحياناً  3. بالكاد أحصل على وقت لنفسي | **كيف يؤثر حجم الوقت الذي تقضيه مع المريض في المستشفى على وقتك لنفسك؟** | 3 |
|  |  | 1. الشريك | **كيف تؤثر حالة المريض على علاقتك بكل من:** | 4 |
|  |  | 2. أفراد العائلة |  |  |
|  |  | 3. الأبناء الآخرون |  |  |
|  |  | 4. الأصدقاء |  |  |
|  |  | 5. زملاء العمل |  |  |
|  |  | د.ب | **كم متوسط صرفك الشهري على علاج المريض؟** | 5 |
|  |  | د.ب | **كم متوسط مدخول الأسرة الشهري من جميع الموارد؟** | 6 |
|  |  |  | **كم عدد الاشخاص العاملين في الأسرة؟** | 7 |
|  |  | 1. الراتب الشهري  2. دعم مالي من العائلة  3. حوالات مالية من الناس  4. استثمال مالي او تجارة  5. أخرى؟ ------------ | **ماهو المصدر الأساسي للإنفاق على علاج المريض؟** | 8 |
|  |  | 1. تأمين صحي خاص  2. تأمين صحي عام أو حكومي  3. تأمين طوارئ  4. ليس لدي تأمين  5. أخرى؟ ------- | **ما نوع التأمين الذي لديك؟** | 9 |
|  |  | 1. نعم  2. لا | **هل لديك تأمين صحي لحالة المريض؟** | 10 |
|  |  | 1. يؤثر على نفقات المطبخ  2. يؤثر على النفقات الدراسية  3 .يؤثر على الإيجار  4. لا يؤثر على الإطلاق  5. أخرى؟ ----------- | **ما تأثير الحالة المزمنة للمريض على الحالة المادية للأسرة؟** | 11 |
|  |  | 1. بيع أغراض المنزل  2. البحث عن عمل جانبي  3. أخذ فرض  4. ليس لدي إستيراتيجية  5. أخرى؟ ----------- | **ماهي إستراتيجيتك للتأقلم مع الإنفاق الصحي في أوقات الأزمات المالية؟** | 12 |
