# Supplementary material for: Caregivers’ experience of seeking care for adolescents with sickle cell disease in a tertiary care hospital in Bahrain
Source: PLoS One. 2022 Apr 7;17(4):e0266501. doi: 10.1371/journal.pone.0266501 (PMC8989311; doi:10.1371/journal.pone.0266501)
Supplement: S4 Appendix — (DOCX) [file pone.0266501.s007.docx]

**دليل المقابلة النوعية :**

الرقم الشخصي للمشارك :

صباحاً/مساءاُ______: ______وقت البدء

صباحاً/مساءاُ _____: ______وقت الانتهاء

______/________/_________التاريخ

القسم أ: المقدمة :

**اسمي (.......)، طالبة طب - السنة الثالثة – في جامعة الخليج العربي، نود من حضرتكم الموافقة للمشاركة في هذه الدراسة التي تهدف الى معرفة الجوانب المختلفة للتحديات التي تواجه مقدمي الرعاية لمرضى فقر الدم المنجلي من فئة المراهقين، ومعرفة ما تأثير العناية بمراهق مصاب بفقر الدم المنجلي على حياتكم الاجتماعية والعائلية والنفسية ودخلكم المالي؛ للوصول إلى فهم دقيق عن المشاكل التي تواجهكم خلال رحلة تقديم الرعاية لمريض فقر الدم المنجلي.**

ملاحظة للباحث**: يرجى قراءة ورقة المعلومات للمشارك وتسجيل إجاباته. بعد ذلك، اقرأ وثيقة الموافقة للمشاركة واطلب من المشارك التوقيع في حال موافقته، و تتم اعتماد الموافقة بعد شرح الأضرار وحقوق المشاركين في هذه الدراسة. يرجى تنبيه المشارك بأن في هذه المقابلة سوف يتم التعرض لبعض الأحداث غير السارة و بعض الأمور العاطفية. يرجى استخدام الدليل أدناه والإجابة على أي سؤال يتعلق بالبحث.**

**شكرًا لك على موافقتك بأن تكون جزءًا من هذه الدراسة. كن واثقاً بأن هويتك وجميع البيانات الشخصية ستعامل بسرية تامة. نتوقع منك الإجابة على جميع الأسئلة بدقة وصدق، يفضل الابتعاد عن الإجابة بــ( نعم أو لا) .**

**في البداية،
هل تستطيع ان تحدثني عن نفسك؟**

أسئلة استقصائية :

**هل يمكن أن تخبرني باسمك وعمرك؟**

**ما هو مستواك التعليمي؟**

**ما هي مهنتك؟**

**هل أنت متروج؟**

**ماذا يقربك المريض؟**

هل يمكن أن تحدثني عن طفلك المصاب بمرض فقر الدم المنجلي؟

أسئلة استقصائية :

**هل يمكن أن تخبرني باسم طفلك وعمره؟**

ملاحظة للباحث**: إذا كان لدى مقدم الرعاية أكثر من طفل مرض مصاب بمرض فقر الدم المنجلي ، اطلب منه التحدث عن طفل واحد فقط.**

القسم ب: تحديات الوصول إلى الرعاية الصحية

الآن، نود أن نعرف عن **تجربتك في استخدام الخدمات الصحي والعناية الطبية المقدمة**، والتي تبدأ منذ لحظة الوصول إلى مركز تقديم العناية الطبية؛ من قبيل الحصول على موعد أو أثناء نوبات السكلر في قسم الطوارئ وحتى تتلقى الرعاية الصحية الكاملة. **فإذًا هل يمكنك أن تخبرني عن تجربتك في استخدام الرعاية الصحية؟**

أسئلة استقصائية :

ما هو الخيار الأول الذي تختاره لتلقي الرعاية الطبية أثناء نوبة طفلك؟

كيف تصل إلى هناك؟

**ما هي الصعوبات التي تواجهك أثناء محاولة الحصول على الرعاية الصحية؟**

أسئلة استقصائية :

الوقت اللازم للوصول إلى المستشفى.

القسم ج: الجانب العاطفي

**ملاحظة للباحث:** انتبه إلى المصطلحات المستخدمة من قبل ولي الأمر المشارك أثناء وصف مشاعرهم. وانتبه إلى لغة الجسد ودع المشارك يتحدث بحرية عن مشاعره دون إصدار أحكام.

**يرجى التعبير عن ما تشعر به اتجاه رعاية مراهق مصاب بمرض فقر الدم المنجلي؟**

أسئلة استقصائية :

كيف تتعامل مع هذا الشعور؟

مع من تتحدث عن ما تشعر به؟

هل لديك صلاحية الوصول إلى أي خدمات الدعم على سبيل المثال مواقع/ مستشارين /توعية ؟ إذا كان الأمر كذلك ، ماذا تستخدم ولماذا؟

العديد من أولياء أ/ور مراهقي السكلر يشعرون بالإرهاق. وأقصد الإرهاق العاطفي والبدني والنفسي الناجم عن الإجهاد المفرط على المدى الطويل. **أخبرني المزيد عن تجربتك مع مثل هذه الأمور.**

أسئلة استقصائية :

كم مرة قلقت في الليل ، أو واجهت مشكلة في النوم أو البقاء نائماً؟

كم مرة شعرت أنك أقل كفاءة / فعالية من ذي قبل أو تعمل بجد أكبر ولكنك تحقق أقل؟

هل شعرت يومًا بالإرهاق؟

كم مرة ترى الأصدقاء والعائلة؟

هل تشعر غالبًا بالغضب أو الانزعاج أو الإزعاج أو الإحباط اتجاه الأشخاص من حولك؟

هل عانيت في أي وقت مضى من المشاكل البدنية؟

القسم د: الجانب الاجتماعي

**ملاحظة للباحث:** ركز على التغيرات التي حدثت في حياة ولي الأمر المشارك بعد إنجاب طفل مصاب بفقر الدم المنجلي
**هل يمكنك أن تخبرني كيف تؤثر رعاية حالة طفلك على حياتك الاجتماعية؟ وأقصد بالحياة الاجتماعية علاقاتك مع محيطك والأنشطة اليومية والتجمعات العائلية.**

أسئلة استقصائية :

كيف تؤثر حالة مريضك على علاقاتك مع عائلتك وزوجك وصداقاتك؟

هل يمكنك أن تخبرني ما هو شعورك حيال علاقاتك الحالية مع أفراد عائلتك وأصدقائك؟

القسم هـ : الجانب المالي

**نحن نتفهم أن هناك الكثير من الأعباء المالية تتعلق بعلاج الطفل المصاب بفقر الدم المنجلي والعناية به، هل يمكنك أن تخبرنا كيف يؤثر الاعتناء به على حالتكم المادية؟**

أسئلة استقصائية :

ماهي المشاكل المالية التي تواجهها؟

كيف تؤثر حالة الطفل على عملكم؟

كيف تستطيع التوفيق بين الاعتناء وعلاج الطفل بالإضافة إلى المتطلبات المادية الأخرى؟

كيف تتعامل مع الأزمات المالية؟

الخاتمة

**نشكركم على مشاركتكم في هذه الدراسة، إن كان لديكم أية إضافات تودون إضافتها أو أسئلة لا تترددوا في طرحها.**

**ملاحظة للباحث:** اسأل المشارك عن إذا ما كان يود إضافة أية معلومات اخرى.
