# Supplementary material for: Caregivers’ experience of seeking care for adolescents with sickle cell disease in a tertiary care hospital in Bahrain
Source: PLoS One. 2022 Apr 7;17(4):e0266501. doi: 10.1371/journal.pone.0266501 (PMC8989311; doi:10.1371/journal.pone.0266501)
Supplement: Attachment — Submitted filename: Manuscript.docx [file pone.0266501.s008.docx]

**The Impact of Being a Caregiver, and Barriers to Seeking Care for Adolescents with Sickle Cell Disease in Bahrain**

Khadija Al Saif^1^, Fatema Mohamed Abdulla^1^, Anwaar Alrahim^1^, Sara Abduljawad^1^, Zainab Matrook^1^, Jenan Jaafar Abdulla^1^, Fatima Bughamar^1^, Fatema Alasfoor^1^, Rana Taqi^1^, Amna Almarzooq^1^, Jamil Ahmed^2^*

Author names and affiliations

Khadija Al Saif	([khadijaaaa@agu.edu.bh](mailto:khadijaaaa@agu.edu.bh))

[orcid.org/0000-0002-1427-880X](https://orcid.org/0000-0002-1427-880X)

Fatema Mohamed Abdulla	([fatemamaa@agu.edu.bh](mailto:fatemamaa@agu.edu.bh))

Anwaar Alrahim	([anwaarhar@agu.edu.bh](mailto:anwaarhar@agu.edu.bh))

Sara Abduljawad	([saraoma@agu.edu.bh](mailto:saraoma@agu.edu.bh)**(**

Zainab Matrook	([zainabm@agu.edu.bh](mailto:zainabm@agu.edu.bh))

Jenan Jaafar Abdulla	([Jenanjaz@agu.edu.bh](mailto:Jenanjaz@agu.edu.bh))

Fatima Bughamar	([fatimakae@agu.edu.bh](mailto:fatimakae@agu.edu.bh))

Fatema Alasfoor	([Fatemamsa@agu.edu.bh](mailto:Fatemamsa@agu.edu.bh))

Rana Taqi	([ranasst@agu.edu.bh](mailto:ranasst@agu.edu.bh))

Amna Almarzooq	([amnaemm@agu.edu.bh](mailto:amnaemm@agu.edu.bh))

Jamil Ahmed MBBS^2^ MPH, FCPS, PhD ([jamilmga@agu.edu.bh](mailto:jamilmga@agu.edu.bh))

[orcid.org/0000-0002-3635-7912](http://orcid.org/0000-0002-3635-7912)

^1^College of Medicine and Medical Sciences, Arabian Gulf University, Manama, Kingdom of Bahrain

^2^Department of Family and Community Medicine, College of Medicine and Medical Sciences, Arabian Gulf University, Manama, Kingdom of Bahrain

Corresponding author’s contact information:

Dr. Jamil Ahmed

Assistant Professor

Department of Family and Community Medicine, College of Medicine and Medical Sciences, Arabian Gulf University, Manama, Kingdom of Bahrain

Email: [jamilmga@agu.edu.bh](mailto:jamilmga@agu.edu.bh), jamil.ahmed.dr@gmail.com

**Conflict of interest:**

None Declared

**Ethical Approval:**

The study was approved by the Research and Ethics Committee of the Arabian Gulf University (No. E008-PI-4/19) and the Health Research Committee of Ministry of Health in Bahrain (No AURS/276/2019). All participants gave written consent after the research details were read out to them by the interviewer.

**The Impact of Being a Caregiver, and Barriers to Seeking Care for Adolescents with Sickle Cell Disease in Bahrain**

**Abstract:**

Objective: We aimed to determine the caregivers’ perspective difficulties in seeking care for their patients with sickle cell disease (SCD). We also explored how caring for SCD patients had social, emotional, and financial impact on the caregivers’ lives.

Study design: We conducted a mixed-method study in a major tertiary care hospital in Bahrain between June and August 2019 with Bahraini caregivers with patients with SCD between aged 10-18 years. In the quantitative part the study design used was cross- sectional and a questionnaire was used to interview 101 caregivers. The qualitative study component was conducted using in-depth interviews with 18 caregivers at Salmaniya Medical Complex. Transcripts of the interviews were analyzed using thematic content analysis.

Results: A lack of parking lots at the hospital (52.5%), traffic jams on the way to the hospital (27%) were identified as the most intriguing challenges to seek care for the SCD patients. These difficulties including prolonged waiting in the emergency room discouraged more than half of the caregivers who preferred to seek care from smaller healthcare centers. Most caregivers reported a high degree of support by their families who emotionally encouraged them to facilitate patient care (73.3%); therefore, their relationship with their friends, colleagues and other family members was not much affected. About 14.8%. of the caregivers suffered catastrophic health expenditures while seeking care for their patients. Three themes emerged from the qualitative analysis: the impact of caring for SCD patients, dissatisfaction with the facilities at the hospitals, services provided during hospital visits were not enough.

Conclusion: Caregivers of SCD patients experienced several challenges that had a negative impact on their lives. These challenges included problems in accessing healthcare and in receiving medical services, in addition to other influences on their emotional, financial, and social aspects of their lives.

# Introduction

Sickle cell disease (SCD) is a group of inherited disorders of red blood cells associated with several health conditions, which typically begin around the age of 5 to 6 months. About 300, 000 to 400, 000 babies are affected with SCD every year, mostly in Sub- Saharan Africa where the prevalence of children with SCD in 2010 was about 230, 000 (1). It is also relatively more prevalent in the Middle Eastern region due to high rates of consanguineous marriages and large family size (2). The prevalence of newborns with SCD in Bahrain has been reported to be about 2.1% to 0.4% from 1985 to 2010 (3, 4). SCD is one of the commonest reasons for hospital admissions in Bahrain and has the highest rate for multiple readmissions. The mortality rate for Bahraini SCD patients in intensive care can be as high as 12.7% (5).

Patients with SCD frequently experience attacks of pain called “Sickle Cell Crisis”, anemia, acute chest syndrome, swelling in the hands and feet, bacterial infections, and even stroke (1). Over a patient’s lifetime, SCD can damage the patient’s organs including spleen, brain, eyes, lungs, liver, heart, kidneys, penis, joints, bones, or skin (6-9). Vaso-occlusive pain is the most common complaint which has an impact upon a patients physical and psychosocial life, and often requires intensive intravenous therapy (10). As the children with SCD move into adolescence, sickle cell pain becomes recurrent, acute, and unpredictable (11). The Sickle Cell Crisis, a sudden appearance of body pain lasting for several hours resulting from vaso-occlusive complications, is a main challenge for both patients and their caregivers leading to poor psychological wellbeing, and health of patients and their caregivers (12-14).

Access to healthcare services can be a challenge for SCD patients’ and their families leading to poor quality of life of patients. Patients also complain about waiting for a long time before receiving care in the emergency department; therefore, it is common that they prefer to treat the symptoms of SCD outside of the health care setting (15). It is also noted that healthcare workers stigmatize patients as drug addicts, and complaints by patients with SCD may not be taken seriously (15). This lack of access to quality healthcare affects patients’ mental and physical health (16).

Caring for an adolescent with a chronic illness adds significant stress and responsibility on the shoulders of caregivers, affecting them emotionally, and mentally (17). Anxiety, depression, exhaustion, poor sleep, and sexual health are the most common ailments reported in caregivers (18). Studies have also reported a higher incidence of depression accompanied with significant guilt, and fear of having another child with SCD (19). Apart from this heavy emotional and mental toll, the caregivers also suffer from an impact on their social lives (20).

Caring for a person with SCD can also impose a huge financial burden on their caregivers. Catastrophic health expenditures can account as high as 20.7% in households having a patient with SCD. The chances of reaching catastrophic health expenditure are higher among parents with poor socioeconomic status who take loans to pay off the hospital fees. In addition, about 2.7% of parents lose their jobs for taking care of their affected children and are unable to commit to work. Also, the indirect cost of missing working days could be huge (21). As many as 63.1% of caregivers can experience financial difficulties (20).

Studies have sparsely focused upon how caring for children and adolescents with chronic disorders affects caregivers. In the Middle Eastern regional countries like Bahrain, where the burden of genetic and inherited disorders requiring long term care is high, there is limited evidence on this topic. Therefore, an evaluation of the impact of being a caregiver of an adolescent with SCD was considered a valuable research area. We targeted this age group because adolescence is a sensitive and critical period of change in the patients’ life where they experience rapid physical, psychological, social, and behavioral changes, requiring a high level of parental guidance and support (22). Adolescent age group is also more prone to adverse symptoms of the SCD (23). The aim of this study was to understand the challenges experienced by caregivers in accessing healthcare for their adolescents with SCD, and to assess the impact of the disease on the caregivers’ own lives.

# Methods

We used a mixed-method study design to meet the objectives of the study. Qualitative research method was included to complement the quantitative design for an in-depth understanding of the research topic.

## Study Setting

The study was conducted at the Salmaniya Medical Complex (SMC), the largest tertiary care hospital in Bahrain from June to August 2019. It is the major public hospital that provides comprehensive medical services including emergency, secondary and tertiary, and outpatient healthcare services. Healthcare services in public hospitals in Bahrain are provided free of charge to all Bahrainis and Gulf Cooperation Council residents. It has a capacity of approximately 1200 beds. The average number of patients received per day is 900 to 1000 (20). A premarital screening program was introduced in Bahrain in 1985 and was expanded in 1992 with an aim to reduce the incidence of genetic diseases. By law, Bahraini couples intending to marry must undergo a compulsory premarital screening. Later, premarital counselling was included as a part of the primary health care services in all healthcare centers throughout Bahrain. In addition to that, a newborn screening program for the detection of blood disorders was started in 2007.

## Selection of Participants

The study participants were primary caregivers of SCD patients aged 10-18 years who were admitted at SMC during the study period. A caregiver was defined as the person who took care of SCD patients and carried most of the responsibility for receiving the patient’s treatment. Participants were identified and recruited with the help of nurse supervisors of the wards and outpatient clinics using a non-probability consecutive sampling technique. We excluded the participants who did not speak Arabic to avoid language barrier. Caregivers were excluded if a SCD patient was less than 10, or older than 18 years. Participants were selected with a ratio of one caregiver to one SCD patient.

## Quantitative methods

### Study Design and Sample

A cross-sectional study was conducted to enroll study participants from the in-patient medical wards and the pediatric outpatient clinic. In January 2019, a pediatric hematology consultant in SMC assumed that the total number of patients under 18 living with SCD in Bahrain were 800, and patients aged 10-18 were 400. The caregivers of 101 patients, or a quarter of all the adolescents with SCD were interviewed in our study. Participants were selected from a list of registered patients provided by nurses. In addition to this sampling technique, some of the participants were added by snow-balled in which we asked caregivers if they knew other caregivers who met our inclusion criteria.

### Data Collection

A structured questionnaire was developed according to the objectives of our study using review of literature. During questionnaire development, researchers made a draft questionnaire, then performed brief qualitative interviews with caregivers to modify the questions. Questionnaire was reviewed by the senior author to ensure that content was appropriate and relevant. A pilot study was conducted to test the validity of the questionnaire. The pretested sample of ten participants suggested no difficulties in comprehending or answering the questions. Using Cronbach’s alpha test, the set of questions had a reliability score of 0.771.

The data collection tool had items on the socio-demographic characteristics of caregivers and the patients, evaluation of healthcare access during Sickle Cell Crisis. We asked questions such as “what is your first destination when the patient has a Sickle Cell Crisis?”, “Why did you choose it as a first destination?”, and “What are the challenges you face on your way to the hospital?”. To assess the influence of being a caregiver on their social, emotional and financial life, we asked questions like “On a scale of 1 to 5, how do you personally evaluate the support you receive from your family?”, “How does the condition of your child affect your relationship with each of the following people?” “On an average how much do you spend on the patient’s treatment in a month?”. Furthermore, the degree of support was measured using a scale of one to five. Family support was recorded as 1-2 (low), 3 (intermediate) and 4-5 (sufficient). Caregivers’ relationship was divided into five categories: relationship with the partner, family members, other children, friends and coworkers. The impact of caregivers’ relationship was considered low if less than two categories were affected. On the contrary, if more than 2 categories were affected then the impact was considered high. The final general assessment section was the calculation of the “catastrophic health expenditure”: [Percentage income spent as health expenditure(*T*_HE/FI_)=(HE_T_/*T*_FI_) × 100 where HE_T_ was the total health expenditure during the month and *T*_FI_ is the total household (family) income during the month (21). Any household with health expenditure proportion (*T*_HE/FI_) ≥10% was designated as having suffered a catastrophic health expenditure.

Data were collected through interviews by five Bahraini year 4 Medical Students from Arabian Gulf University who received training on data collection from the senior author. The purpose of the investigation was explained in person to the participants. Next, the interviewers filled the questionnaires in Arabic for the participants in a room provided by the hospital to ensure confidentiality during data collection. Each interviewer completed 20 questionnaires and each interview lasted for about 10 to 20 minutes. The data collectors checked the questionnaires for incomplete data at the end of interviews.

### Data analysis

Data were translated and entered to Microsoft Excel and then double-checked for accuracy. Later, data were imported to Statistical Package for the Social Sciences version 20, where they were coded and analyzed. In descriptive statistics, quantitative variables such as mean and standard deviation were calculated to describe the age of caregivers and patients, and for other variables proportions or absolute values are presented.

## Qualitative methods

### Study design and sample

An exploratory qualitative approach was used to understand the challenges faced by caregivers of Bahraini SCD adolescent patients in accessing healthcare, and to understand how caring for patients with SCD had an impact on their own lives. We expected that a qualitative approach would allow caregivers to reveal their experiences in dealing with the difficulties faced as caregivers and expressing their views of how their children’s conditions affected their own lives. A total of 18 caregivers participated in our study, 15 of them were selected from medical wards and three from outpatient clinics. We interviewed 11 caregivers from the younger patients' age group (10-14 years), and 7 of adolescent (15-18 years).

### Data collection

The interviews were conducted by five Bahraini year 4 Medical Students from Arabian Gulf University who received training from the senior author, an experienced qualitative researcher, to conduct interactive in-depth interviews using a semi-structured in-depth interview guide in Arabic. Each interview lasted approximately 40 minutes. Qualitative interviews were also conducted in a room provided by the hospital to ensure confidentiality of data collection. Before interviewing, the purpose of the research was explained to the participants. Caregivers were informed that discussions would be audio tapped and can be stopped if discomfort occurs during recording. The interviews included open-ended questions such as: “Can you tell me about the difficulties you face while trying to seek healthcare for your child?” “How do you feel about taking care of your child/adolescent with SCD?” “How does it affect your relationship with your family and friends?”, “How did your child’s condition affect your financial status?” probes like: “Can you tell me more about that” were used. By the time we interviewed 18 primary caregivers, we reached data saturation and the study team agreed to discontinue any further interviews.

### Data analysis

We analyzed the qualitative data through thematic content analysis. Audio recorded interviews were transcribed verbatim by the interviewers themselves. Manual coding of the Arabic interviews was performed in a table format and codes were applied to text in an adjacent column. After a consensus between the study team was reached, the codes were revised and finalized. The generation of codes depended mainly on the importance of the data and/or repetition of the same data in several interviews. During analysis, codes were translated into English and reviewed sequentially with the qualitative research expert for better accuracy and rationality of patterns. Codes led to the generation of categories and themes. Representative quotes under the codes were translated from Arabic to English.

### Ethical Consideration

The study was approved by the Research and Ethics Committee of the Arabian Gulf University (No E008-PI-4/19) the Health Research Committee of Ministry of Health in Bahrain (No AURS/276/2019). Administrative permission was also obtained from the SMC. A written informed consent was signed by the interviewees. Participants were informed that they had the freedom to withdraw at any time of the study, and their agreement or refusal would not interfere with their healthcare in the hospital. Names were replaced with their initials and other personal information about participants was concealed to maintain privacy. We assured participants that they had adequate time during the interview to ease stress, reveal their fears, and eliminate hesitation while taking into consideration their body language and facial expressions. We expressed empathy and gave them complete attention during the interviews.

# Results

## Quantitative results:

We excluded six questionnaires with significant missing data. Only data from complete questionnaires were analyzed. Approximately 67% of the caregivers were Bahrainis aged 40 to 50 years old with a mean age of 44.24 years (SD± 6.25), 77.2% were females, 92.1% were married, and 73.3% were mothers of the patients. About 48.5% of the caregivers were unemployed, and 41.6% were high school graduates. Among the SCD patients, 41.6% were 13 to 15 years old, while 32.7% of them were 10 to 12 years old with a mean age of 13.73 years (SD ± 2.37) (Table 1).

About half (52.5%) of the caregivers complained about lack of parking space at the hospital, while 26.7% stated that they had traffic jam problems on their way to the hospital or health center. In case of a Sickle Cell Crisis, 56.4% of the caregivers preferred to take their patients to the health centers compared to 25.7% who preferred to visit SMC. About 15.8% used traditional medicine or simple analgesics and 6.9% went to private clinics to manage the painful Sickle Cell Crisis. When we asked the caregivers to choose a reason for selecting a particular medical facility as the first destination, most of the caregivers who preferred going to the health centers agreed that this was due to its proximity to their houses and also due to the good quality of the services. On the other hand, those who preferred going to SMC were more satisfied with the quality of services. In addition, some participants complained about the delay in the accidents and emergency department of SMC which was the reason they preferred other facilities which had a faster response than SMC (Fig. 1).

**Table 1. Socio-demographic characteristics of the caregivers and patients with SCD in Bahrain (n= 101).**

|  | | N |
| --- | --- | --- |
| ***Caregivers*** | |  |
| Age (Years) | < 40 | 18 |
|  | 40-50 | 67 |
|  | > 50 | 15 |
| Gender | Male | 23 |
|  | Female | 78 |
| Marital status | Single | 2 |
|  | Married | 93 |
|  | Divorced | 2 |
|  | Widowed | 4 |
| Relationship with patient | Mother | 74 |
|  | Father | 23 |
|  | Other relatives | 4 |
| Current occupation | Employed | 52 |
|  | Unemployed | 49 |
| Educational level | Illiterate | 1 |
|  | Primary | 3 |
|  | Intermediate | 15 |
|  | High school | 41 |
|  | Diploma | 13 |
|  | Bachelor | 20 |
|  | Master | 7 |
| ***Patients*** |  |  |
| Age (Years) | 10-12 | 33 |
|  | 13-15 | 42 |
|  | 16-18 | 26 |
| Gender | Male | 55 |
|  | Female | 46 |

**Fig 1. Caregivers’ reasons for selecting various types of health facilities as a first destination for sickle cell crisis.** (* other reasons: to avoid admission, mild crisis).

Among the caregivers of SCD patients, 73.3% had a high family support and about 81.2% of the support was an emotional support which they described as listening and showing empathy. Other types of support the participants said they received was financial support by 18.8%, informational support by 23.8%, and 4% of caregivers had received any other form of support from their families. Most caregivers’, 73%, had a low impact on their relationship with others. Although, 48.5% of caregivers stated that the time for themselves was moderately affected because of the time they have spent with their patients, 39.6% complained that their own time was extremely affected. Regarding the financial aspect, we found that 14.8% of the caregivers had catastrophic health expenditures as they spent more than 10% of total family income on their patients’ health. More than half of the caregivers who suffered catastrophic health expenditures reported that other family expenses were affected too. The mean out of pocket expenditures on SCD care was 16.72 Bahraini Dinar (1 Bahraini Dinar=2.65 USD) per month with a range from 0 to 150 Bahraini Dinars. The mean monthly household was 776 Bahraini Dinar with a median of 500 Bahraini Dinar.

## Qualitative results

The caregivers were all Bahraini aged between 30 years to 50 years, 17 out of 18 interviews were with females. Three distinct but interrelated themes were identified in our analysis: the impact of caring for SCD patients, dissatisfaction with the facilities at the hospitals, services provided during hospital visits were not enough. (Fig 2) shows a summary of the coding process, subthemes, and themes.

**Fig 2. Summary of the coding process, subthemes, and themes.**

**1. Impact of caring for SCD patients**

***Subtheme A: feelings of fear, regret, and guilt***

Caregiver’s lives were most affected emotionally and physically. Several caregivers discussed their fear that they wanted to conceive but they were afraid of having another child with SCD. Caregivers also expressed fear of losing their child with SCD due to the disease or its complications especially when Sickle Cell Crisis when the child suffers the most.

“I became pregnant with my second daughter; I was too scared that she may also be born with the disease.” (mother of 16 year old female,47, IDI-18)

“Once she (the child) gets sick I always say I will lose her, because of the stories I have heard about death among SCD patients, although her attacks are mild, but once she gets an attack I get too scared that I might lose her.“(mother of 14 years old female,38, IDI-4)

Many caregivers showed intense feelings of guilt and regret because of marrying a carrier husband or not performing the premarital test. This feeling was also strong during the crises. They often blamed themselves for being the reason for their children’s pain. However, some mothers had no regrets because it was something that they did not select, and the pre- marital test was not mandatory.

“I cry a lot, because I am in pain and regret, you cannot even imagine how much I blame myself.” (mother of 10 years old male,38, IDI-1)

“I regret getting married to a sickle cell carrier, these kids are killing me inside if I can go back in time I would not get married to the same person.” (mother of 18 years old female,43, IDI-8)

Most caregivers were shocked the moment their children were diagnosed with SCD. Many of them collapsed and spent sleepless nights just watching their children to make sure they were fine and thinking about their fate with the disease. Many had at least one relative with SCD; therefore, they knew what these patients went through.

“It is a shock to know that your child is sick with sickle cell disease.” (mother of 19 years old male, IDI-19)

Out of this feeling of guilt, some caregivers always apologized to their children for having SCD; they felt that they were the reason for the misery and not enjoying a normal childhood.

“In the beginning I told her, I apologize for bringing you to this life, and you are the only one who is sick in this house, you did not have a normal life or normal childhood. And when she got older, she did not get the chance to have a life and play like the other kids at her age.” (mother of 18 years old female,43, IDI-8)

***Subtheme B: Emotional and health burden***

Most caregivers faced psychological stress from prolonged hospital stay during their children’s admissions. They discussed how they would spend sleepless nights in hospitals, and at home, and were always under stress. They felt chronically fatigued, anxious, and sad which led them to desire to cry and breakdown. Although caregivers had a huge emotional burden, they tried to cope with it by believing that their situation was better than others.

“I am psychologically in pain. I am tired, I am tired.” (mother of a 14-year-old female, 38, IDI-4)

“In hospital he meets a lot of patients with worse medical conditions. Allah loves you and that is why he gifted you with this disease, look at the people around you who cannot walk, hear and some of them have even lost their parents, Allah gave you something and took something from you, you should thank Allah for all the blessings that you have.” (mother of 10 years old male,34, IDI-16)

Some of the caregivers said they would not talk with other people openly about their emotional burden. They always act as everything was fine in front of their families to avoid creating a sad environment.

“I get emotionally affected but I try not to make my husband and my son aware of this because my husband gets affected a lot and collapses so I should stay strong.” (mother of 14 years old male, IDI-7)

This emotional burden, psychological stress, overthinking, and insomnia often led to physical problems in caregivers. They complained of several health issues that they suffered from ranging from back pain to gastrointestinal, and cardiovascular symptoms.

“I think a lot, to the point that my blood pressure and blood sugar levels increase from overthinking, I get anxious a lot.” (mother of 12 years old male, 39, IDI-2)

“I suffer from some health issues, I suffer from spasms, and stomach pain and it increases with my anxiety when I care for her, I make sure that I will not make her aware of this, the spasms increase a lot while I am worried and angry.” (mother of 18 years old female,43, IDI-8)

Although most mothers stated that their relationship with their husbands was not affected as they supported each other in caring for the child. Some mothers, however, stated that their relationship with their husbands was affected even since they knew that their child suffered from SCD. A reason for this was that mothers were often angry with their husbands and families because of the psychological stress. Some mothers also indicated that their husbands would not bear to see their children in pain, or because they were busy with their work.

“He (the husband) told my family that I have changed since I became pregnant with my daughter, and I changed more once I knew that she is sick with sickle cell disease, since then he has changed too.” (mother of 18 years old female, 43, IDI-8)

Tired and out of the mood, the caregivers believed they needed to socialize more with their families and friends, however, they were always worried about leaving the child alone without their supervision. That is the reason most caregivers indicated that they missed family and friends’ gatherings and special events.

“I used to go out with my husband’s family, and my friends but now (after having the affected child) I have disappeared, no one sees me!” (mother of 10 years old male,40, IDI-10)

***Subtheme C: cost of care and burden on families***

Some caregivers indicated that they had often been unable to meet some of the needs of the families. Caregivers who had a poor financial status have their own strategies to face the financial crises which can occur specially when their children are hospitalized. Some of them stated that they borrow money from their relatives, while others receive monthly support from the charities. At least two caregivers mentioned that despite their urgent needs, a charity refused to give them stipend anymore, under the pretext that there were more deserving families. Caregivers discussed how embarrassing it was for them and their husbands to take loans.

“My husband’s salary is only 150 BD (Bahraini Dinar), so it’s not enough.” (mother of 10 years old male, IDI-19)

“We put so much pressure on ourselves and lend money from my husband’s sister, but he does not like to ask her, it is so hard on men to do such a thing.” (mother of 16 years old female,47, IDI-18)

One factor which could have financially burdened the caregivers was that their jobs were affected while they were busy caring for their affected children. Some of them had to leave their jobs while others’ jobs were affected adversely as they cared for the children, and others had numerous abstinence leaves because of the frequent hospitalizations.

“I used to work, but now I resigned to take care of my girl.” (mother of 16 years old female,47, IDI-18)

Another factor that seemed to have worsened the financial state of our participants was that their expenses increased when their children were hospitalized. Most Caregivers said that buying food and medications during hospital admissions significantly affected their budget.

“To be honest the food is extremely costly for me, because he does not like the hospital’s food.” (mother of 10 years old male, 38, IDI-1)

**2. Dissatisfaction with the facilities at the hospital**

Majority of caregivers discussed experiencing delays in the emergency room. They must wait for several hours before receiving the treatment. In addition, they must wait for several hours and sometimes days in the emergency room until their patients are shifted to the appropriate wards.

“It takes us hours until we are referred to the emergency room and then we also wait hours until they come to give him (the child) the medicine.” (mother of 10 years old male,40, IDI-10)

“my daughter waited for the next day in the emergency room, they did not refer her to the ward for two days.” (mother of 17 years old female,42, IDI-11)

Most of the participants indicated that they suffered from fatigue and back pain because there was no place for the accompanying person to sleep in the wards. Only mothers can stay with their children during hospitalization in the pediatric wards. Often, they sleep on the floor during the stay in the hospital which is sometimes prolonged from about two to five weeks.

“Just imagine, the mother sleeps on the chair or on the floor and not for a day or two, they stay like this for about three to five weeks. It is extremely hard, with all this, it is a not surprising to get physically fatigued.” (mother of 10 years old male,34, IDI-16)

Also, some caregivers stated that the patient’s beds in wards and emergency rooms were uncomfortable which caused worsening of pain or even radiation of pain to other parts of the body.

“my daughter enters the hospital for example due to pain in her arm, but the pain radiates to her back, she (my daughter) said because of the beds, they are uncomfortable.” (mother of 18 years old female, IDI-8)

According to many caregivers, the space designated for patients with SCD in the main tertiary care hospital was scarce and hardly accommodated the large number of patients. Therefore, wards and emergency room were usually crowded with patients and their accompanying relatives creating an uncomfortable atmosphere.

“The place for patients with SCD in the hospital is very, very small, it does not accommodate all this number (of patients).” (mother of 18 years old female,43, IDI-8)

Another problem which was identified by most caregivers is a lack of a separate ward in the hospital dedicated for patients with SCD. Also, some of them wished for a separate building for females with SCD and older than 15 years; because currently they were scattered within different wards, while male SCD patients have their own separate building.

“Males with sickle cell disease have a separate building but females do not, until when female’s patients will suffer from this?” (mother of 18 years old female,43, IDI-8)

**3. Services provided during hospital visits were not enough**

Caregivers preferred to visit health centers than hospitals, because of the previous unpleasant experience of other family members in the large hospital on several occasions. For example, the availability of medication and response to patients is much better in health centers than hospitals.

“In the health center the care is much better. They let us in as we reach, bring the doctor, and treat him (the patient), but in hospitals I have to wait for 3 hours (before we receive the treatment).” (mother of 12 years old male ,38, IDI-12)

Many caregivers mentioned that hospital staff are respectful, have a good behavior, and they are always welcoming to the patients. They also mentioned that doctors as well as nurses were humble and treated their patients as if they are part of their own families.

“Honestly, I cannot say anything bad about them, they try their best with my son as if they're his parent.” (mother of 10 years old male,38, IDI-1)

However, many caregivers reported that one of the main issues that their children faced was the need to be followed up by a pediatrician. However, currently, they are transferred to an adult ward after they turn fifteen. Thus, unfortunately, pediatricians are not always available for them in those wards. Moreover, caregivers stated the existing number of doctors and nurses in the emergency room did not meet the patient needs.

“It's emergency room! There should have been more doctors and nurses, one or two will not be enough for a huge waiting line.” (mother of 17 years old female, 42, IDI-11)

Caregivers believed that there are some health workers who required to more experience in perform minor procedures. They discussed how some nurses were less experienced in finding veins to inject medicines, leading to irritation, pain, and anxiety in their children. Also, they claimed that the emergency room physicians need to be more skillful in treating SCD patients.

“Nurses were joyful with my child, they were nice but while taking blood, my child was screaming." (mother of 17 years old male, 40, IDI-9)

Caregivers also indicated that even though most of the prescribed medications were provided by the hospital, some of them, such as Diclofenac Sodium, were usually not available. They must buy it out of pocket most of the time. This was not the only problem regarding medications; in addition, caregivers were concerned about using Hydroxyurea as the drug of choice for their patients because of a wide range of side effects the drug caused. They feared that their children may become infertile after using Hydroxyurea. This fear originated from observing close relatives with SCD using Hydroxyurea and the conversations with caregivers of other patients in the hospital. However, the caregivers also acknowledged that medical staff counselled them to alleviate their doubts and concerns.

“I was scared once I gave her the (Hydroxyurea) capsules; people would tell me that it will lead to hair loss and problems in getting pregnant. “(mother of 10 years old female,50, IDI-13).

Some of the caregivers stated that the laboratory tests in the private hospitals were faster than in public hospitals. Caregivers preferred to go to private hospitals for laboratory tests before their visits to the public hospitals. Other caregivers stated that private clinics did not differ much from public hospitals and that the treatment was equal in both type of hospitals.

“Sometimes I prefer to go to private hospitals to get the blood tests done, because the public hospitals take time and maybe he won't need to stay in hospital so I just skip the 7 or 8 hours waiting (in the emergency room) and (directly) go to a private hospital.” (mother of 10 years old male, 34, IDI-16)

# Discussion

This mixed-method study aimed to investigate the health care access problems faced by caregivers of SCD patients and the impact of being a caregiver on various aspects of their lives. Difficulties with parking in the hospital, and prolonged waiting in the emergency room discouraged more than half of the caregivers who preferred to seek care from smaller healthcare centers. A lack of separate ward for the patients with SCD and dissatisfaction with some of the facilities and services at the major tertiary care hospital were some of the major challenges these caregivers experienced while seeking care for their children. Significant emotional and financial burden was accompanied with fear and regrets about their children, which could be associate with stress and physical symptoms among caregivers. The most pertinent of the results are discussed below.

The caregiving had a massive impact upon the caregivers. Many were afraid of losing their children due to the complications of the disease and worried about having another child born with SCD. It was also common that caregivers had to stay in the hospital for long periods of time while their children were hospitalized, which affected their psychological health negatively. More than one-thirds of the participants in our qualitative study complained of psychological stress which sometimes deteriorated their physiological and mental wellbeing. Our results are consistent with a cross-sectional study from Saudi Arabia which showed that emotional health is one of the most affected aspects of the caregivers’ quality of life (24). Our findings are also consistent with at least four studies from African region, where the burden of SCD is also the highest in the world, which also show a high psychological burden on the caregivers of patients with SCD (18, 25-27).

Regret was another common feeling in our participants. Some caregivers regretted to marry a carrier husband, whom they believed as the reason for their children’s pain. Other caregivers had no regret because the premarital test was not mandatory at the time they were married. Despite the huge emotional burden on caregivers, the quantitative analysis showed that, 81.2% of them received emotional support from their families. This emotional support is necessary for the primary caregivers whose quality of life is often affected by long term care that they provide to their sick children (19).

Although our quantitative analysis showed that 73.3% of caregivers had a low impact of caring for their sick children on their relationships with partners, children, relatives, friends, and coworkers, the qualitative findings were different from this. Our qualitative results showed that social lives of caregivers seemed to have drastically affected as they were scared to leave their children without their attention. The qualitative findings are different perhaps because the caregivers freely talked about their challenges in an in-depth discussion. Many socially active caregivers had stopped participating in their family and friends’ gathering to look after their children. Even other siblings of the patients felt jealous or neglected unintentionally because of the extra-attention the caregivers gave to the affected child. Our findings are consistent with a study from Nigeria that used a SCD burden interview where caregivers of SCD reported neglecting other family members because of caring for their affected children (12).

Financial burden was variable among caregivers. Many of them had no financial problems, while others indicated their poor financial status which worsened when the child experienced Sickle Cell Crisis and was hospitalized. Caregivers gave several reasons for their worsening financial status. First, the expenses increase during admissions as they must buy food because most children would not like hospital food, while others buy some medications which are not usually provided by the hospital. Second, some caregivers must be absent several times or even leave their jobs to take care of their children which impacted their earnings (28). Our results are consistent with a study from Nigeria which showed that 56.8% of SCD caregivers lost between 1 and 48 working days due to taking care of their affected child and 3 caregivers lost their jobs due to the same reason (21). The quantitative analysis of our data also showed that 14.8% of caregivers suffered catastrophic health expenditures, which is considered relatively high based on the free of cost nature of healthcare service in Bahrain. Catastrophic health expenditures on caring for SCD patients can be as high as 20.7% in some countries (21).

Patients with SCD require frequent visits to emergency department or outpatient department because of the persistent symptoms which may be sometimes difficult to treat (29). However, the visits to emergency departments may not be smooth. Long waiting hours in the emergency room was a major problem faced by most of the caregivers, supported by our qualitative analysis. As a result, the caregivers often preferred home management with different analgesics for pain relief during Sickle Cell Crisis before considering seeking healthcare service. The delay in the emergency room also determined their preference for either home management or going to primary healthcare centers during the Sickle Cell Crises. Some caregivers stated that there was a shortage of the staff in the emergency room which we believe might be associated with delays in providing the treatment. Studies even from USA have shown that SCD patients experience significant delays in receiving treatment for SCD patients in the emergency departments (15).

Another major problem in accessing health care was that SCD patients, especially females older than fifteen years, did not have their own dedicated ward. They were scattered among different adult wards assigned to diseases other than SCD even though they are still treated by pediatricians during their hospitalization who are not always available in adult wards, which makes it difficult for the caregivers to know about their children’s condition. In addition, many of the patients disliked going to the hospital during crises because they cannot get along with adult patients, unlike when they were in pediatric wards where most of the patients are around their age. An innovative and culturally appropriate solution may be implemented with involvement of multidisciplinary team to enhance satisfaction, and health outcomes in SCD patients (30).

## Strengths and limitations:

The main strength of our study was a mixed-method study design consisting of qualitative and quantitative components with complemented each other. Limitations of our study included that the participants in the qualitative part were mainly caregivers of hospitalized patients, while most of the participants in the quantitative part were caregivers of patients from out-patient clinics. The hospitalized patients usually have moderate-to-severe disease and those in out-patient clinics have mostly mild disease. This may also clarify why the results from some aspects of this study are not consistent with others; such as the impact on the relationship of caregivers where the qualitative analysis revealed high impact, while the quantitative analysis showed a low impact on relationships with their family and friends. Another limitation is that the qualitative and the quantitative studies were conducted at the same time; therefore, many unexpected new findings were revealed by the qualitative analysis which were not identified by the quantitative questionnaire. This can be explored in future research where the items from our qualitative analysis could be included in a more robust quantitative study identifying the gaps in service delivery to meet the expectations of the caregivers and their patients. The perspective of the providers and administrators in the hospital was not sought in this study, which may be different than those of the patients. There could be operational reasons for delays in the emergency room, which also need to be explored further.

## Conclusion:

Caregiver’s lives were mainly affected by their children’s disease where they were found to be living under a huge emotional burden of fear, regrets, and guilt. This can affect the health of caregivers and affect the care for the individuals with SCD, resulting in a heavy burden on the healthcare system. Caregivers’ experience of care in the major tertiary care hospital can be improved by overcoming the barriers to their physical access to the facilities and improving the service delivery issues like long waiting time in the emergency room and providing a dedicate hospitalization space for female adolescents with SCD.

## Acknowledgements:

We are grateful to the parents of SCD patients who participated in our research. We are also grateful to the nurses in the pediatric ward and outpatient department of the SMC who helped us in finding the participants. We also thank Mr. Amer AlMarabeh, biostatistician at the Arabian Gulf University, for his help with the quantitative data analysis.

# References

1.	Kato GJ, Piel FB, Reid CD, Gaston MH, Ohene-Frempong K, Krishnamurti L, et al. Sickle cell disease. Nat Rev Dis Primers. 2018;4:18010.

2.	El-Hazmi MA, Al-Hazmi AM, Warsy AS. Sickle cell disease in Middle East Arab countries. Indian J Med Res. 2011;134(5):597-610.

3.	Al Arrayed S, Al Hajeri A. Public awareness of sickle cell disease in Bahrain. Ann Saudi Med. 2010;30(4):284-8.

4.	Al Arrayed S, Al Hajeri A. Newborn Screening Services in Bahrain between 1985 and 2010. Adv Hematol. 2012;2012:903219.

5.	Al Khawaja SA, Ateya ZM, Al Hammam RA. Predictors of mortality in adults with sickle cell disease admitted to intensive care unit in Bahrain. J Crit Care. 2018;43:378-9.

6.	Shah N, Bhor M, Xie L, Paulose J, Yuce H. Sickle cell disease complications: Prevalence and resource utilization. PLoS One. 2019;14(7):e0214355.

7.	AlRyalat SA, Nawaiseh M, Aladwan B, Roto A, Alessa Z, Al-Omar A. Ocular Manifestations of Sickle Cell Disease: Signs, Symptoms and Complications. Ophthalmic Epidemiol. 2020;27(4):259-64.

8.	Ochocinski D, Dalal M, Black LV, Carr S, Lew J, Sullivan K, et al. Life-Threatening Infectious Complications in Sickle Cell Disease: A Concise Narrative Review. Front Pediatr. 2020;8:38.

9.	Sachdev V, Rosing DR, Thein SL. Cardiovascular complications of sickle cell disease. Trends Cardiovasc Med. 2020.

10.	LeBlanc Z, Vance C, Payne J, Zhang J, Hilliard L, Lebensburger JD, et al. Management of severe chronic pain with methadone in pediatric patients with sickle cell disease. Pediatr Blood Cancer. 2018;65(8):e27084.

11.	Sil S, Cohen LL, Bakshi N, Watt A, Hathaway M, Abudulai F, et al. Changes in Pain and Psychosocial Functioning and Transition to Chronic Pain in Pediatric Sickle Cell Disease: A Cohort Follow-up Study. Clin J Pain. 2020;36(6):463-71.

12.	Kumar G, Al Muwaijei AA, Sohal APS. Sickle cell crisis: A crisis of a different sort? Arch Dis Child Educ Pract Ed. 2018;103(6):290-336.

13.	Telfer P, Bahal N, Lo A, Challands J. Management of the acute painful crisis in sickle cell disease- a re-evaluation of the use of opioids in adult patients. Br J Haematol. 2014;166(2):157-64.

14.	Wright J, Ahmedzai SH. The management of painful crisis in sickle cell disease. Curr Opin Support Palliat Care. 2010;4(2):97-106.

15.	Evensen CT, Treadwell MJ, Keller S, Levine R, Hassell KL, Werner EM, et al. Quality of care in sickle cell disease: Cross-sectional study and development of a measure for adults reporting on ambulatory and emergency department care. Medicine (Baltimore). 2016;95(35):e4528.

16.	McGann S. A Caregiver's Perspective: Internist, Active Duty Service Member, and Mother of a Child Living With Sickle Cell Disease. Ann Emerg Med. 2020;76(3S):S78-S9.

17.	Josset-Raffet E, Duparc-Alegria N, Thiollier AF, Dugue S, Faye A, Ithier G, et al. [Perception and assessment of pain by caregiver and adolescent with sickle cell disease: The impact of the patient's anxiety]. Arch Pediatr. 2016;23(2):143-9.

18.	Bioku AA, Ohaeri JU, Oluwaniyi SO, Olagunju TO, Chaimowitz GA, Olagunju AT. Emotional distress among parent caregivers of adolescents with sickle cell disease: Association with patients and caregivers variables. J Health Psychol. 2020:1359105320935986.

19.	van den Tweel XW, Hatzmann J, Ensink E, van der Lee JH, Peters M, Fijnvandraat K, et al. Quality of life of female caregivers of children with sickle cell disease: a survey. Haematologica. 2008;93(4):588-93.

20.	Karadag G, Gungormus Z, Olcar Z. Experiences and Problems Encountered by Families of Children with Sickle Cell Anemia. J Caring Sci. 2018;7(3):125-9.

21.	Olatunya OS, Ogundare EO, Fadare JO, Oluwayemi IO, Agaja OT, Adeyefa BS, et al. The financial burden of sickle cell disease on households in Ekiti, Southwest Nigeria. Clinicoecon Outcomes Res. 2015;7:545-53.

22.	Oyedeji C, Strouse JJ. Improving the Quality of Care for Adolescents and Adults With Sickle Cell Disease-It's a Long Road. JAMA Netw Open. 2020;3(5):e206377.

23.	Esezobor CI, Akintan P, Nwaogazie U, Akinwunmi E, Temiye E, Akinsulie A, et al. Enuresis in children and adolescents with sickle cell anaemia is more frequent and substantially different from the general population. PLoS One. 2018;13(8):e0201860.

24.	Madani BM, Al Raddadi R, Al Jaouni S, Omer M, Al Awa MI. Quality of life among caregivers of sickle cell disease patients: a cross sectional study. Health Qual Life Outcomes. 2018;16(1):176.

25.	Kuerten BG, Brotkin S, Bonner MJ, Ayuku DO, Njuguna F, Taylor SM, et al. Psychosocial Burden of Childhood Sickle Cell Disease on Caregivers in Kenya. J Pediatr Psychol. 2020;45(5):561-72.

26.	Tubman VN. Mother knows best: The caregiver's perspective on clinical trials for sickle cell disease highlights the challenges of providing care in Africa. Pediatr Blood Cancer. 2020;67(2):e28054.

27.	Ohaeri JU, Shokunbi WA. Psychosocial burden of sickle cell disease on caregivers in a Nigerian setting. J Natl Med Assoc. 2002;94(12):1058-70.

28.	Brown BJ, Okereke JO, Lagunju IA, Orimadegun AE, Ohaeri JU, Akinyinka OO. Burden of health-care of carers of children with sickle cell disease in Nigeria. Health Soc Care Community. 2010;18(3):289-95.

29.	Brown SE, Weisberg DF, Sledge WH. Family caregiving for adults with sickle cell disease and extremely high hospital use. J Health Psychol. 2016;21(12):2893-902.

30.	Kingsley RA. A Healthcare Improvement Initiative to Increase Multidisciplinary Pain Management Referrals for Youth with Sickle Cell Disease. Pain Manag Nurs. 2020.

## Supporting Information:

S1 Data. Software Data. (SAV)

S1 Appendix. Survey Questionnaire. (DOCX)

S2 Appendix. In-depth Interview Guide. (DOCX)

S1 Fig. Caregivers’ reasons for selecting various types of health facilities as a first destination for sickle cell crisis. (* other reasons: to avoid admission, mild crisis). (TIFF)

S2 Fig. Summary of the coding process, subthemes, and themes. (TIFF)
