# Supplementary material for: Caregivers’ experience of seeking care for adolescents with sickle cell disease in a tertiary care hospital in Bahrain
Source: PLoS One. 2022 Apr 7;17(4):e0266501. doi: 10.1371/journal.pone.0266501 (PMC8989311; doi:10.1371/journal.pone.0266501)
Supplement: Attachment — Submitted filename: Response to reviewers.docx [file pone.0266501.s009.docx]

***Reference number: PONE-D-20-34450***

**Dear Editor Rachel A. Annunziato,**

**Thank you for giving us the opportunity to submit a revised draft of our manuscript titled [Barriers to Seeking Care for Adolescents with Sickle Cell Disease in a Tertiary Care Hospital in Bahrain: Caregiver Experiences] to *PLOS ONE*. We appreciate the time and effort that you and the reviewers have dedicated to providing your valuable feedback on our manuscript. We are grateful to the reviewers for their generous comments on our paper. We have been able to incorporate changes to reflect most of the suggestions provided by the reviewers and revised the manuscript accordingly. We have highlighted the changes within the manuscript. Our responses to these comments are given under each of your comment in italics.**

1. Please ensure that your manuscript meets PLOS ONE's style requirements, including those for file naming. The PLOS ONE style templates can be found at

<https://journals.plos.org/plosone/s/file?id=wjVg/PLOSOne_formatting_sample_main_body.pdf>

and

<https://journals.plos.org/plosone/s/file?id=ba62/PLOSOne_formatting_sample_title_authors_affiliations.pdf>

**Response to editor:** *Thank you for reminding us about it. We have made sure to follow the journal guidelines. We reviewed the style requirements and made the necessary changes.*

2. Thank you for providing the English version of the questionnaires. Please also include a copy of both questionnaires in the original language, as Supporting Information, or include a citation if it has been published previously.

**Response to editor:** *We added both questionnaires in the original language as supporting information.*

3. For the quantitative study, please provide further details on sample size and power calculations.

**Response to editor:** *We provided details on the sample size and power calculations in the methods section with citations.*

4. We note that you have indicated that data from this study are available upon request. PLOS only allows data to be available upon request if there are legal or ethical restrictions on sharing data publicly. For information on unacceptable data access restrictions, please see <http://journals.plos.org/plosone/s/data-availability#loc-unacceptable-data-access-restrictions>.


In your revised cover letter, please address the following prompts:


a) If there are ethical or legal restrictions on sharing a de-identified data set, please explain them in detail (e.g., data contain potentially identifying or sensitive patient information) and who has imposed them (e.g., an ethics committee). Please also provide contact information for a data access committee, ethics committee, or other institutional body to which data requests may be sent.


b) If there are no restrictions, please upload the minimal anonymized data set necessary to replicate your study findings as either Supporting Information files or to a stable, public repository and provide us with the relevant URLs, DOIs, or accession numbers. Please see <http://www.bmj.com/content/340/bmj.c181.long> for guidelines on how to de-identify and prepare clinical data for publication. For a list of acceptable repositories, please see <http://journals.plos.org/plosone/s/data-availability#loc-recommended-repositories>.


We will update your Data Availability statement on your behalf to reflect the information you provide.

**Response to editor:** *Given your concern, we provided the analysis process for both the quantitative and qualitive methods in the supporting information files. This is the minimal anonymized data set required to replicate our study findings.*

5. Your ethics statement should only appear in the Methods section of your manuscript. If your ethics statement is written in any section besides the Methods, please delete it from any other section.

**Response to editor:** *We revised the full manuscript to ensure that the ethics statements appear only in the Methods section.*

6. Please upload a copy of Figure 1 and 2, to which you refer in your text on page 12 and 13. If the figure is no longer to be included as part of the submission please remove all reference to it within the text. We note that Figure 1 and 2 may be duplicate of your Supplementary Figures.

**Response to editor:** *We uploaded a copy of Figures 1 and 2 from our text.*

7. Please include captions for ALL your Supporting Information files at the end of your manuscript, and update any in-text citations to match accordingly. Please see our Supporting Information guidelines for more information: <http://journals.plos.org/plosone/s/supporting-information>

**Response to editor:** *We included captions for all our supporting information files at the end of the manuscript. We also updated the in-text citations to match the supporting information file.*

**Here is a point-by-point response to the reviewers’ comments and concerns.**

**Comments from Reviewer #1:**

- **Comment 1:** This is in an important study with the aim of determining the caregivers’ difficulties in seeking care for their patients with sickle cell disease. The view of the caregiver is often ignored when research is conducted into chronic diseases/disorders like sickle cell disease. The authors need to thoroughly edit their work, few spelling errors are highlighted in the manuscript.
- **Response to reviewer*:*** *Thank you for your comments. We reread the paper carefully and hired a professional to correct all the grammatical errors and provide English editing.*

Other comments are included in the manuscript:

- **Comment 2:** The authors should consider rephrasing this sentence, it currently read as if it’s the care giver who is receiving treatment.
- **Response to reviewer:** *We rephrased the sentence to clarify the meaning.*
- **Comment 3:** This should be cited appropriately either as personal communication or unpublished research.
- **Response to reviewer:** *We agree with this comment. We do not have any statistics available that give us the exact number of adolescents with SCD aged 10-18 years in Bahrain during 2019. However, we cited the number of our target population by the recorded number of newborns with SCD in published research. For the rest of our target population, we mentioned the published number of students with SCD in each academic school year in newspapers.*
- **Comment 4:** In the event that there were incomplete data, what did the data collectors do?
- **Response to reviewer:** *We did not use questionnaires that did not fulfill the purpose and objectives of the study. Questions that caregivers could not fill out because the answers differed at various times and questions that were sensitive to the caregiver were omitted from the calculations, though we did include these caregivers in the study.*
- **Comment 5:** Translated by who? Double checked by who?
- **Response to reviewer:** *It was translated and double checked by the researchers. We included their initials in the manuscript.*
- **Comment 6:** How were these participants selected from the larger group of 101 caregivers?
- **Response to reviewer:** *Out of 101 caregivers in the quantitative study, only two caregivers were included in the qualitative study randomly. We conducted the two studies separately and we did not select participants from other part of the study.*
- **Comment 7:** According to WHO adolescence starts from the age of 10. Why did the authors choose to label this group adolescent?
- **Response to reviewer:** *Thank you for noticing the typo. We meant to separate adolescence into early adolescence (10-14 years of age) and late adolescence (15-18 years of age).*
- **Comment 8:** The age of some caregivers is not given, this should be corrected to make it uniform.
- **Response to reviewer:** *We provided the quotes with the caregivers’ ages.*
- **Comment 9:** ‘absence’?
- **Response to reviewer:** *The sentence has been changed to absence.*
- **Comment 10:** This was not a finding in this particular study.
- **Response to reviewer:** *Thank you for noticing this statement. We modified it with the proper studies.*

**Comments from Reviewer #2:**

- **Comment 1:** This study describes the impacts of caregiving among relatives of adolescents with SCD and the barriers to care in a Bahraini tertiary hospital. Importantly, the study provides important perspectives on the experience of caregivers of adolescents with SCD in Bahrain. However, the paper needs substantial improvement in the style of writing and correction of grammatical/typo errors.
- **Response to reviewer:** *Thank you for your time and effort in reviewing our article. This review helped us improve the quality and value of the article. We edited our work and corrected the errors.*

Title:

- **Comment 2:** Given that the study was conducted in a single tertiary hospital, it is much better to indicate this in the title unless there is clear justification that the study sample is representative of national population of Bahrain.
- **Response to reviewer** *Thank you, we have updated the title. We revised the title to be more concise.*
- **Comment 3:** The paper needs English Language editing and revisions of typo errors
- **Response to reviewer:** *We hired a professional editor to review and improve the language of the article.*

Abstract:

- **Comment 4:** Typographical errors should be corrected. For instance, the authors are encouraged to revise the first sentence in the abstract:
  ” We aim to determine the perspective of caregivers on the difficulties encountered while seeking care for their adolescents with sickle cell disease (SCD)”.
- **Response to reviewer:** *We revised the abstract and changed this sentence.*
- **Comment 5:** The authors are encouraged to use first person language throughout the paper. For example, the phrase: “SCD patients” should be changed to “adolescents with SCD”
- **Response to reviewer:** *We changed the wording “SCD patients” to “adolescents with SCD” when appropriate throughout the paper.*
- **Comment 6:** The sentence: “In the quantitative part of the study, we used……...” needs revision to improve clarity.
- **Response to reviewer:** *We revised this sentence.*
- **Comment 7:** The rationale for an emphasis on “Salmaniya Medical Complex” here is not entirely clear.
- **Response to reviewer:** *Thank you for pointing this out. We removed this sentence from the abstract.*
- **Comment 8:** The authors are encouraged to revise the sentence: “the impact of caring for SCD patients, dissatisfaction with the facilities at the hospitals, services provided during hospital visits were not enough”. This sentence is not clear.
- **Response to reviewer:** *We modified the themes and explained them in the abstract.*

Introduction:

- **Comment 9:** The second sentence in the introduction alluded that the prevalence of SCD in the Middle Eastern region is driven by “large family size”. It is important for the author to provide the scientific basis for making this conclusion.
- **Response to reviewer:** *We removed this text as we did not find a scientific basis for this conclusion.*
- **Comment 10:** The introduction can be improved by operationalizing the main study outcome(s), and describing the study objectives/postulations/or hypotheses.
- **Response to reviewer:** *We revised the Introduction. We tried our best to find literature related to the main study outcomes. We expanded the last paragraph of the Introduction to provide more clarity on the objectives/rationale of the study.*

Methods:

- **Comment 11:** The rationale for the sample size should be included with citations, if available. If the estimation of the sample size or power calculation is not feasible, this should be included in the study limitations.
- **Response to reviewer:** *Thank you for this thoughtful suggestion. We have provided the sample size estimation and power calculations, with citations.*
- **Comment 12:** The authors are encouraged to confirm informed consent/assent from caregivers versus adolescents.
- **Response to reviewer:** *Thank you for pointing this out. We clarified this statement.*
- **Comment 13:** It will be nice to include information on the study response rate.
- **Response to reviewer:** *Unfortunately, this data is not available.*
- **Comment 14:** A copy of the study questionnaire should be provided as supplementary material.
- **Response to reviewer:** *We provided the study questionnaire as supplementary material (S1 Appendix). We categorized the answers into different sections during the analysis process to clarify the results.*
- **Comment 15:** The layout of the methods needs improvement to ensure clarity. For example, the quantitative and qualitative components of the study can be described side by side in a logical and clear fashion. This will help avoid the creation of two subsections on data-analysis
- **Response to reviewer:** *Thank you for your suggestion. We combined the subheadings accordingly and explained both components side by side when appropriate. For clarity, we split the heading of the data collection to help readers understand the data collection process in a more simplified way.*

Results:

- **Comment 16:** Revisions to correct typos and all grammatical errors
- **Response to reviewer:** *Thank you for your comment. We had the manuscript reviewed for appropriate language use and to correct all grammatical errors.*
- **Comment 17:** The sentence: “Three distinct but ……………….” should have “and’’ connecting the third reasons listed.
- **Response to reviewer:** *We modified this sentence.*
- **Comment 18:** The authors are encouraged to revise the presentation of the data collected from the qualitative assessment. The results of the qualitative data are currently difficult to follow and to lengthy
- **Response to reviewer:** *Thank you for these valuable suggestions. We revised the results as suggested to clarify the data and minimize the word count.*
- **Comment 19:** Table legend should be provided to describe all abbreviations used in the table
- **Response to reviewer:** *Thank you for pointing this out. We described and removed all abbreviations in the table.*

Discussion:

- **Comment 20:** The use of the phrase, “catastrophic health expenditures” sounds somewhat awkward.
- **Response to reviewer:** *Thank you for your feedback. There are a lot of research studies conducted using this equation and it is discussed on the WHO website. We believe replacing the phrase CHE can change the interpretation of the study. We used the definition of CHE in the discussion.*
- **Comment 21:** In the absence of a clearer presentation of the shortcomings associated with receiving care from health centers that are closer to the residence of the study participants residence, the caregiver’s preference in attending these centers for treatment compared to the tertiary center seems reasonable.
- **Response to reviewer:** *We did not address the shortcomings associated with each health facility in our quantitative study. This will be included in our limitations, as we believe that this topic can be explored in-depth in the future.*
- **Comment 22:** The cross-sectional study design, a lack of hypothesis-driven sample size estimations or power calculation, and data was collected in one tertiary hospital limit the generalization of the study findings. This should be included the study shortcomings.
- **Response to reviewer:** *We included this discussion in the limitations paragraph and elaborated further on this study’s limitations.*

**In addition to the above comments, all spelling and grammatical errors pointed out by the reviewers have been corrected. We look forward to hearing from you in due time regarding our submission and to respond to any further questions and comments you may have.**

**Kind regards**
