# Supplementary material for: Caregivers’ experience of seeking care for adolescents with sickle cell disease in a tertiary care hospital in Bahrain
Source: PLoS One. 2022 Apr 7;17(4):e0266501. doi: 10.1371/journal.pone.0266501 (PMC8989311; doi:10.1371/journal.pone.0266501)
Supplement: Attachment — Submitted filename: Response to Reviewers.docx [file pone.0266501.s010.docx]

***Reference number: PONE-D-20-34450***

***Dear Editor,***

***Thank you for allowing us to re-submit a revised draft of our manuscript titled [Barriers to Seeking Care for Adolescents with Sickle Cell Disease in a Tertiary Care Hospital in Bahrain: Caregiver Experiences] to PLOS ONE. We appreciate the time and effort that you and the reviewers have dedicated to providing your valuable feedback on our manuscript. We are grateful to the reviewers for their generous comments on our paper. We have been able to incorporate changes to reflect most of the suggestions provided by the respectful reviewers and revised the manuscript accordingly. We have highlighted these changes within the manuscript. Our responses to these comments are given under each of your comment in italics.***

Journal Requirements:

Please review your reference list to ensure that it is complete and correct. If you have cited papers that have been retracted, please include the rationale for doing so in the manuscript text, or remove these references and replace them with relevant current references. Any changes to the reference list should be mentioned in the rebuttal letter that accompanies your revised manuscript. If you need to cite a retracted article, indicate the article’s retracted status in the References list and also include a citation and full reference for the retraction notice.

***Response to Editor: Thank you for your concern. We have ensured that all our cited papers are not retracted. We have added new references in our revised manuscript as required by the reviewers.***

[Note: HTML markup is below. Please do not edit.] Reviewers' comments:

Reviewer's Responses to Questions

**Comments to the Author**

1. If the authors have adequately addressed your comments raised in a previous round of review and you feel that this manuscript is now acceptable for publication, you may indicate that here to bypass the “Comments to the Author” section, enter your conflict of interest statement in the “Confidential to Editor” section, and submit your "Accept" recommendation.

Reviewer #2: All comments have been addressed Reviewer #3: (No Response)

2. Is the manuscript technically sound, and do the data support the conclusions?

The manuscript must describe a technically sound piece of scientific research with data that supports the conclusions. Experiments must have been conducted rigorously, with appropriate controls, replication, and sample sizes. The conclusions must be drawn appropriately based on the data presented.

Reviewer #2: Yes

Reviewer #3: Yes

3. Has the statistical analysis been performed appropriately and rigorously?

Reviewer #2: Yes

Reviewer #3: Yes

4. Have the authors made all data underlying the findings in their manuscript fully available?

The [PLOS Data policy](http://www.plosone.org/static/policies.action#sharing) requires authors to make all data underlying the findings described in their manuscript fully available without restriction, with rare exception (please refer to the Data Availability Statement in the manuscript PDF file). The data should be provided as part of the manuscript or its supporting information, or deposited to a public repository. For example, in addition to summary statistics, the data points behind means, medians and variance measures should be available. If there are restrictions on publicly sharing data—e.g. participant privacy or use of data from a third party—those must be specified.

Reviewer #2: Yes

Reviewer #3: Yes

5. Is the manuscript presented in an intelligible fashion and written in standard English?

PLOS ONE does not copyedit accepted manuscripts, so the language in submitted articles must be clear, correct, and unambiguous. Any typographical or grammatical errors should be corrected at revision, so please note any specific errors here.

Reviewer #2: Yes

Reviewer #3: Yes

6. Review Comments to the Author

Please use the space provided to explain your answers to the questions above. You may also include additional comments for the author, including concerns about dual publication, research ethics, or publication ethics. (Please upload your review as an attachment if it exceeds 20,000 characters)

Reviewer #2: This is to thank the authors for the revisions conducted.

I wonder if a reference (a primary citation if available) can be provided for the phrase, “catastrophic health expenditures” where it was fist used in the main text to help readers understand that it is a common construct.

***Response to Reviewer: Thank you for this suggestion. We have provided references of catastrophic health expenditures definition in the introduction.***

Reviewer #3: This study aims to evaluate the caregivers experiences of caring for an adolescent child with SCD in terms of access to health care, as well as the impact on the caregiver’s own emotional, psychological, financial and physical well-being.

The study is clear, with conclusions supported by data generated through this research. There could be more emphasis on implications of the findings on policy change (i.e. in the hospital) or suggested improvements in terms of support that can be provided for caregivers since the negative impact on caregivers’ psychosocial and physical wellbeing is evident in the findings. Some suggestions are highlighted briefly in the conclusion section but this could be expanded on. For example, is there scientific evidence that suggests that formal support options are beneficial to caregivers? Such as support groups, other forms of psychosocial support/counseling? There are a few typographical errors that need to be addressed.

***Response to Reviewer: Thank you for your suggestion. We have expanded the suggestions in the conclusion section. We have provided as well scientific evidence that suggest formal support can be of beneficial to caregivers. We have addressed typographical errors in the conclusion section.***

Please take note of the comments and questions below for minor revisions:

1. Authors are encouraged to review referencing of statements:
   a. Line 36-38 – Can you reference the statistic of SCD prevalence globally?

***Response to Reviewer: Thank you for this suggestion. Quoted from the first study referenced (keto et al) “The number of all-age individuals affected by SCA globally is currently unknown and cannot be estimated reliably owing to the paucity of epidemiological data, in particular mortality data, in areas of high prevalence.”***

b. Line 42-44 – Can you reference the statistic concerning contribution of SCD to hospital admissions in Bahrain?

***Response to Reviewer: Thank you for this suggestion. We have added the references concerning the contribution of SCD to hospital admissions in Bahrain.***

c. Line 58 – Can you reference the studies that note that caregivers experience shock?

***Response to Reviewer: Thank you for this suggestion. It was only one study in our references.***

2. Authors are encouraged to revise text that duplicates, is unclear or contradictory:
   a. Compare lines 50-52 with lines 53-57. The former is captured in the latter. The latter would be sufficient. The authors are encouraged to revise these lines.

***Response to Reviewer: Thank you for this suggestion. We have removed this sentence as the latter is sufficient.***

b. Line 59 – States that premarital testing for SCD is non-mandatory. Is this contradicted in lines 124-125? The authors are encouraged to review these statements and revise as needed.

***Response to Reviewer: In 2004, premarital SCD testing became mandatory in Bahrain. However, the results of the premarital test does not their decision to marry by law. A new citation was added in the methods sections. Line 59 involves another country. We have removed this sentence.***

c. Line 89-90 – The study is about the caregivers’ experiences, but this statement refers to the patients only. Either omit, or include caregivers. Please review.

***Response to Reviewer: Thank you for this suggestion. We have removed this sentence.***

d. Line 147 – Please clarify the years stated on this line: ‘in 2006 – 2001’ – should this read 2001 – 2006?

***Response to Reviewer: Thank you for this suggestion. We have clarified the years to read like this 2001-2006.***

e. Line 177 – Please review. Suggest to change ‘with’ to ‘by’ or ‘conducted by’ to make it clear that it was the students collecting the data

***Response to Reviewer: Thank you for this suggestion. We have made the sentence clear as the students were data collectors.***

f. Line 236 – Please review this line as it lacks clarity. …‘interviewers the researchers…’ – is there a comma missing or should the word interviewers be omitted?

***Response to Reviewer: Thank you for this suggestion. We have removed the word interviewers.***

g. Line 289 – change ‘w’ to ‘with’

***Response to Reviewer: Thank you for this suggestion. We have changed this word.***

h. Line 300-301 – ‘and 4% of caregivers received any other form of support from their families’ – lacks clarity – what is ‘any other form of support’

***Response to Reviewer: Thank you for your suggestion. We have clarified that sentence as most caregivers mentioned accompanying their affected child as other form of support from their families.***

i. Line 301-302 – ‘Most caregivers (73%) had a low impact on their relationships with others.’ Incomplete statement – what had a low impact? Should this read ‘For most caregivers (73%), caring for an adolescent with SCD had a low impact on their relationships with others’?

***Response to Reviewer: Thank you for your suggestion. Yes. We have clarified that sentence.***

j. Line 304 = ‘their patients’ – suggest to change this to ‘their children with SCD’ or ‘their affected children’

***Response to Reviewer: Thank you for your suggestion. We have changed this to “their affected children***

3. Authors are encouraged to include definitions to terms such as adolescents and catastrophic health expenditure, with appropriate referencing:
   a. Line 70 – The authors are encouraged to define ‘Catastrophic health expenditures’ in this section. If using an official WHO definition, this should include a reference.

***Response to Reviewer:*** ***Thank you for this suggestion. We have provided references of catastrophic health expenditures definition in the introduction.***

b. Line 96 – The study refers to adolescents. Authors are encouraged to review the need to include a definition of the adolescent age group in this section, providing a reference.

***Response to Reviewer: Thank you for this suggestion. We have added the definition of adolescent age group according to WHO with a reference from the official Brtannica web page.***

The authors are encouraged to review the final statements of the introductory section. Is there a broader goal/impact that this study might have, which can be stated here? For example, to provide additional support to caregivers, improve access to care etc. What do the authors plan to address or achieve through this study? This can then be expanded on in the conclusion section based on findings from the study.

***Response to Reviewer: Thank you for this suggestion. We have added the impact that the authors aimed to achieve in the final introduction statement.***

Selection of participants – The authors are encouraged to review this section to address how the participants for the qualitative study were selected? This is referred to later under ‘Study Design and Sample’ but should be included in the ‘selection of participants’ section.

***Response to Reviewer: Thank you for your inquiry. We have moved the additional sampling technique from study design and sample section to the selection of participants’ section. The selection of qualitative and quantitative were both consecutive sampling except some quantitative participants were added by snowball sampling as mentioned in the selection of participant section.***
